# Supplementary material for: Metallacarborane Synthons for Molecular Construction—Oligofunctionalization of Cobalt Bis(1,2-dicarbollide) on Boron and Carbon Atoms with Extendable Ligands
Source: Molecules. 2023 May 16;28(10):4118. doi: 10.3390/molecules28104118 (PMC10221555; doi:10.3390/molecules28104118)
Supplement: Supplementary file 1 [file molecules-28-04118-s001.zip › molecules-2372264-supplementary.pdf]

Supporting Information

Metallacarborane Synthons for Molecular Construction—Oligofunctionalization of Cobalt Bis(1,2-dicarbollide) on Boron and Carbon Atoms with Extendable Ligands

Krzysztof Śmiałkowski [1,2], Carla Sardo [1,3] and Zbigniew J. Leśnikowski [1,*]

1 Laboratory of Medicinal Chemistry, Institute of Medical Biology Polish Academy of Sciences, Lodowa 106, 93-232 Lodz, Poland; ksmialkowski@cbm.pan.pl (K.Ś.); csardo@unisa.it (C.S.)

2 Lodz Institutes of the Polish Academy of Science, The Bio-Med-Chem Doctoral School, University of Lodz, 90-237 Lodz, Poland

3 Department of Pharmacy, University of Salerno, via Giovanni Paolo II, 132, 84084 Fisciano, SA, Italy

* *Correspondence: zlesnikowski@cbm.pan.pl; Tel.: +48-42-209-33-80*

Figure S1. MS (ESI) spectrum of 3,3'-Co{[(8-O(CH2)4OCPh3]-1,2-C2B9H10}(8'-OH-1',2'-C2B9H10)(CH3)2N[(CH2)4OPh3]2 **(5)** 6
Figure S2. ^{1}H NMR spectrum of 3,3'-Co[(8-O(CH2)4OCPh3-1,2-C2B9H10)]2 (CH3)2N[(CH2)4OPh3]2 **(7)** 6
Figure S3. ^{13}C{^{1}H} NMR spectrum of 3,3'-Co[(8-O(CH2)4OCPh3-1,2-C2B9H10)]2 (CH3)2N[(CH2)4OPh3]2 **(7)** 7
Figure S4. ^{11}B{^{1}H} NMR spectrum of 3,3'-Co[(8-O(CH2)4OCPh3-1,2-C2B9H10)]2 (CH3)2N[(CH2)4OPh3]2 **(7)** 7
Figure S5. ^{11}B NMR spectrum of 3,3'-Co[(8-O(CH2)4OCPh3-1,2-C2B9H10)]2 (CH3)2N[(CH2)4OPh3]2 **(7)** 8
Figure S6. MS (ESI) spectrum of 3,3'-Co[(8-O(CH2)4OCPh3-1,2-C2B9H10)]2 (CH3)2N[(CH2)4OPh3]2 **(7)** 8
Figure S7. ^{1}H NMR spectrum of 3,3'-Co[8-O(CH2)3OTBDMS]-1,2-C2B9H10)(8'-OH-1,2-C2B9H10) (CH3)2N[(CH2)3OTBDMS)]2 **(6)** 9
Figure S8. ^{13}C{^{1}H} NMR spectrum of 3,3'-Co[8-O(CH2)3OTBDMS]-1,2-C2B9H10)(8'-OH-1,2-C2B9H10) (CH3)2N[(CH2)3OTBDMS)]2 **(6)** 9
Figure S9. ^{11}B{^{1}H} NMR spectrum of 3,3'-Co[8-O(CH2)3OTBDMS]-1,2-C2B9H10)(8'-OH-1,2-C2B9H10) (CH3)2N[(CH2)3OTBDMS)]2 **(6)** 10
Figure S10. ^{11}B NMR spectrum of 3,3'-Co[8-O(CH2)3OTBDMS]-1,2-C2B9H10)(8'-OH-1,2-C2B9H10) (CH3)2N[(CH2)3OTBDMS)]2 **(6)** 10
Figure S11. MS(ESI) spectrum of 3,3'-Co[8-O(CH2)3OTBDMS]-1,2-C2B9H10)(8'-OH-1,2-C2B9H10) (CH3)2N[(CH2)3OTBDMS)]2 **(6)** 11
Figure S12. ^{1}H NMR spectrum 3,3'-Co[8-O(CH2)3OTBDMS-1,2-C2B9H10)]2 (CH3)2N[(CH2)3OTBDMS)]2 **(8)**. 11
Figure S13. ^{13}C{^{1}H} NMR spectrum of 3,3'-Co[8-O(CH2)3OTBDMS-1,2-C2B9H10)]2 (CH3)2N[(CH2)3OTBDMS)]2 **(8).** 12
Figure S14. ^{11}B{^{1}H} NMR spectrum of 3,3'-Co[8-O(CH2)3OTBDMS-1,2-C2B9H10)]2 (CH3)2N[(CH2)3OTBDMS)]2 **(8).** 12
Figure S15. ^{11}B NMR spectrum of 3,3'-Co[8-O(CH2)3OTBDMS-1,2-C2B9H10)]2 (CH3)2N[(CH2)3OTBDMS)]2 **(8).** 13
Figure S16. MS(ESI) spectrum of 3,3'-Co[8-O(CH2)3OTBDMS-1,2-C2B9H10)]2 (CH3)2N[(CH2)3OTBDMS)]2 **(8).** 13
Figure S17. ^{1}H NMR spectrum of [3,3'-Co(8-O(CH2)3OH-1,2-C2B9H10)2] TBA **(9).** 14
Figure S18. ^{13}C{^{1}H} NMR spectrum of [3,3'-Co(8-O(CH2)3OH-1,2-C2B9H10)2] TBA **(9).** 14
Figure S19. ^{11}B{^{1}H} NMR spectrum of [3,3'-Co(8-O(CH2)3OH-1,2-C2B9H10)2] TBA **(9).** 15
Figure S20. ^{11}B NMR spectrum of [3,3'-Co(8-O(CH2)3OH-1,2-C2B9H10)2] TBA **(9).** 15
Figure S21. MS(ESI) spectrum of [3,3'-Co(8-O(CH2)3OH-1,2-C2B9H10)2] TBA **(9).** 16
Figure S22. MS (ESI) spectrum of 3,3'-Co{[8-O(CH2)3OTBDMS-1-(CH2)2OH]-1,2-C2B9H9)}[8'-O(CH2)3OTBDMS-1',2'-C2B9H10)]$^-$ **(10)** 16
Figure S23. ^{1}H NMR spectrum of 3,3'-Co[(8-O(CH2)3OTBDMS-1-(CH2)2OH-1,2-C2B9H9)]2$^-$ **(11)** 17
Figure S24. ^{13}C{^{1}H} NMR spectrum of 3,3'-Co[(8-O(CH2)3OTBDMS-1-(CH2)2OH-1,2-C2B9H9)]2$^-$ **(11)** 17
Figure S25. ^{11}B{^{1}H} NMR spectrum of 3,3'-Co[(8-O(CH2)3OTBDMS-1-(CH2)2OH-1,2-C2B9H9)]2$^-$ **(11)** 18
Figure S26. ^{11}B NMR spectrum of 3,3'-Co[(8-O(CH2)3OTBDMS-1-(CH2)2OH-1,2-C2B9H9)]2$^-$ **(11)** 18
Figure S27. MS (ESI) spectrum of 3,3'-Co[(8-O(CH2)3OTBDMS-1-(CH2)2OH-1,2-C2B9H9)]2$^-$ **(11)** 19

Figure S28. ^{1}H NMR spectrum of 8,8'-bridged [8,8'-$O_2P(O)H$-3,3'-Co(1,2-$C_2B_9H_{10})_2$] $HNEt_3$ H-phosphonate **(12)** 19
Figure S29. $^{13}C\{^{1}H\}$ NMR spectrum 8,8'-bridged [8,8'-$O_2P(O)H$-3,3'-Co(1,2-$C_2B_9H_{10})_2$] $HNEt_3$ H-phosphonate **(12)** 20
Figure S30. $^{11}B\{^{1}H\}$ NMR spectrum of 8,8'-bridged [8,8'-$O_2P(O)H$-3,3'-Co(1,2-$C_2B_9H_{10})_2$] $HNEt_3$ H-phosphonate **(12)** 20
Figure S31. ^{11}B NMR spectrum of 8,8'-bridged [8,8'-$O_2P(O)H$-3,3'-Co(1,2-$C_2B_9H_{10})_2$] $HNEt_3$ H-phosphonate **(12)** 21
Figure S32. $^{31}P\{^{1}H\}$ NMR spectrum of 8,8'-bridged [8,8'-$O_2P(O)H$-3,3'-Co(1,2-$C_2B_9H_{10})_2$] $HNEt_3$ H-phosphonate **(12)** 21
Figure S33. ^{31}P NMR spectrum of 8,8'-bridged [8,8'-$O_2P(O)H$-3,3'-Co(1,2-$C_2B_9H_{10})_2$] $HNEt_3$ H-phosphonate **(12).** 22
Figure S34. MS (ESI) spectrum of 8,8'-bridged [8,8'-$O_2P(O)H$-3,3'-Co(1,2-$C_2B_9H_{10})_2$] $HNEt_3$ H-phosphonate **(12).** 22
Figure S35. FT-IR spectrum of 8,8'-bridged [8,8'-$O_2P(O)H$-3,3'-Co(1,2-$C_2B_9H_{10})_2$] $HNEt_3$ H-phosphonate **(12)** 23
Figure S36. UV-VIS spectrum of 8,8'-bridged [8,8'-$O_2P(O)H$-3,3'-Co(1,2-$C_2B_9H_{10})_2$] $HNEt_3$ H-phosphonate **(12)** 23
Figure S37. ^{1}H NMR spectrum of 8,8'-bridged [8,8'-$O_2P(O)SH$-3,3'-Co(1,2-$C_2B_9H_{10})_2$] HDBU phosphorothioate **(13).** 24
Figure S38. $^{13}C\{^{1}H\}$ NMR spectrum of 8,8'-bridged [8,8'-$O_2P(O)SH$-3,3'-Co(1,2-$C_2B_9H_{10})_2$] HDBU phosphorothioate **(13).** 24
Figure S39. $^{11}B\{^{1}H\}$ NMR spectrum of 8,8'-bridged [8,8'-$O_2P(O)SH$-3,3'-Co(1,2-$C_2B_9H_{10})_2$] HDBU phosphorothioate (**13**)**.** 25
Figure S40. ^{11}B NMR spectrum of 8,8'-bridged [8,8'-$O_2P(O)SH$-3,3'-Co(1,2-$C_2B_9H_{10})_2$] HDBU phosphorothioate **(13).** 25
Figure S41. $^{31}P\{^{1}H\}$ NMR spectrum of 8,8'-bridged [8,8'-$O_2P(O)SH$-3,3'-Co(1,2-$C_2B_9H_{10})_2$] HDBU phosphorothioate **(13).** 26
Figure S42. ^{31}P NMR spectrum of 8,8'-bridged [8,8'-$O_2P(O)SH$-3,3'-Co(1,2-$C_2B_9H_{10})_2$] HDBU phosphorothioate **(13).** 26
Figure S43. MS (ESI) spectrum of 8,8'-bridged [8,8'-$O_2P(O)SH$-3,3'-Co(1,2-$C_2B_9H_{10})_2$] HDBU phosphorothioate **(13).** 27
Figure S44. FT-IR spectrum of 8,8'-bridged [8,8'-$O_2P(O)SH$-3,3'-Co(1,2-$C_2B_9H_{10})_2$] HDBU phosphorothioate **(13).** 27
Figure S45. UV-VIS spectrum of spectrum of 8,8'-bridged [8,8'-$O_2P(O)SH$-3,3'-Co(1,2-$C_2B_9H_{10})_2$] HDBU phosphorothioate **(13).** 27
Figure S46. ^{1}H NMR spectrum of [8,8'-$O_2P(O)S(CH_2)_4OCPh_3$-3,3'-Co(1,2-$C_2B_9H_{10})_2$] $HNEt_3$ **(15)** 28
Figure S47. $^{13}C\{^{1}H\}$ NMR spectrum of [8,8'-$O_2P(O)S(CH_2)_4OCPh_3$-3,3'-Co(1,2-$C_2B_9H_{10})_2$] $HNEt_3$ **(15)** 28
Figure S48. $^{11}B\{^{1}H\}$ NMR spectrum of [8,8'-$O_2P(O)S(CH_2)_4OCPh_3$-3,3'-Co(1,2-$C_2B_9H_{10})_2$] $HNEt_3$ **(15)** 29
Figure S49. ^{11}B NMR spectrum of [8,8'-$O_2P(O)S(CH_2)_4OCPh_3$-3,3'-Co(1,2-$C_2B_9H_{10})_2$] $HNEt_3$ **(15)** 29
Figure S50. $^{31}P\{^{1}H\}$ NMR spectrum of [8,8'-$O_2P(O)S(CH_2)_4OCPh_3$-3,3'-Co(1,2-$C_2B_9H_{10})_2$] $HNEt_3$ **(15)** 30
Figure S51. ^{31}P NMR spectrum of [8,8'-$O_2P(O)S(CH_2)_4OCPh_3$-3,3'-Co(1,2-$C_2B_9H_{10})_2$] $HNEt_3$ **(15)** 30
Figure S52. MS (ESI) spectrum of [8,8'-$O_2P(O)S(CH_2)_4OCPh_3$-3,3'-Co(1,2-$C_2B_9H_{10})_2$] $HNEt_3$ **(15)** 31

Figure S53. FT-IR spectrum of [8,8'-$O_2P(O)S(CH_2)_4OCPh_3$-3,3'-Co(1,2-$C_2B_9H_{10})_2$] $HNEt_3$ **(15)** 31
Figure S54. UV-VIS spectrum of [8,8'-$O_2P(O)S(CH_2)_4OCPh_3$-3,3'-Co(1,2-$C_2B_9H_{10})_2$] $HNEt_3$ **(15)** 31
Figure S55. ^{1}H NMR spectrum of [8,8'-$O_2P(O)S(CH_2)_3OCPh_3$-3,3'-Co(1,2-$C_2B_9H_{10})_2$] $HNEt_3$ **(16)** 32
Figure S56. $^{13}C\{^1H\}$ NMR spectrum of [8,8'-$O_2P(O)S(CH_2)_3OCPh_3$-3,3'-Co(1,2-$C_2B_9H_{10})_2$] $HNEt_3$ **(16)** 32
Figure S57. $^{11}B\{^1H\}$ NMR spectrum of [8,8'-$O_2P(O)S(CH_2)_3OCPh_3$-3,3'-Co(1,2-$C_2B_9H_{10})_2$] $HNEt_3$ **(16)** 33
Figure S58. ^{11}B NMR spectrum of [8,8'-$O_2P(O)S(CH_2)_3OCPh_3$-3,3'-Co(1,2-$C_2B_9H_{10})_2$] $HNEt_3$ **(16)** 33
Figure S59. $^{31}P\{^1H\}$ NMR spectrum of [8,8'-$O_2P(O)S(CH_2)_3OCPh_3$-3,3'-Co(1,2-$C_2B_9H_{10})_2$] $HNEt_3$ **(16)** 34
Figure S60. ^{31}P NMR spectrum of [8,8'-$O_2P(O)S(CH_2)_3OCPh_3$-3,3'-Co(1,2-$C_2B_9H_{10})_2$] $HNEt_3$ **(16)** 34
Figure S61. MS (ESI) spectrum of [8,8'-$O_2P(O)S(CH_2)_3OCPh_3$-3,3'-Co(1,2-$C_2B_9H_{10})_2$] $HNEt_3$ **(16)** 35
Figure S62. FT-IR spectrum of [8,8'-$O_2P(O)S(CH_2)_3OCPh_3$-3,3'-Co(1,2-$C_2B_9H_{10})_2$] $HNEt_3$ **(16)** 35
Figure S63. UV-VIS spectrum of [8,8'-$O_2P(O)S(CH_2)_3OCPh_3$-3,3'-Co(1,2-$C_2B_9H_{10})_2$] $HNEt_3$ **(16)** 35
Figure S64. ^{1}H NMR spectrum of 4-(1,3-bis(trityloxy)propan-2-yloxy)butyl4-methylbenzenesulfonate **(20)** 36
Figure S65. $^{13}C\{^1H\}$ NMR spectrum of 4-(1,3-bis(trityloxy)propan-2-yloxy)butyl4-methylbenzenesulfonate **(20)** 36
Figure S66. MS (ESI) spectrum of 4-(1,3-bis(trityloxy)propan-2-yloxy)butyl4-methylbenzenesulfonate **(20)** 37
Figure S67. FT-IR spectrum of spectrum of 4-(1,3-bis(trityloxy)propan-2-yloxy)butyl4-methylbenzenesulfonate **(20)** 37
Figure S68. UV-VIS spectrum of 4-(1,3-bis(trityloxy)propan-2-yloxy)butyl4-methylbenzenesulfonate (20) 37
Figure S69. ^{1}H NMR spectrum of [8,8'-$O_2P(O)S(CH_2)_4OCH(CH_2OTr)_2$-3,3'-Co(1,2-$C_2B_9H_{10})_2$]$HNEt_3$ **(21)** 38
Figure S70. $^{13}C\{^1H\}$ NMR spectrum of [8,8'-$O_2P(O)S(CH_2)_4OCH(CH_2OTr)_2$-3,3'-Co(1,2-$C_2B_9H_{10})_2$]$HNEt_3$ **(21)** 38
Figure S71. $^{11}B\{^1H\}$ NMR spectrum of [8,8'-$O_2P(O)S(CH_2)_4OCH(CH_2OTr)_2$-3,3'-Co(1,2-$C_2B_9H_{10})_2$]$HNEt_3$ **(21)** 39
Figure S72. ^{11}B NMR spectrum of [8,8'-$O_2P(O)S(CH_2)_4OCH(CH_2OTr)_2$-3,3'-Co(1,2-$C_2B_9H_{10})_2$]$HNEt_3$ **(21)** 39
Figure S73. $^{31}P\{^1H\}$ NMR spectrum of [8,8'-$O_2P(O)S(CH_2)_4OCH(CH_2OTr)_2$-3,3'-Co(1,2-$C_2B_9H_{10})_2$]$HNEt_3$ **(21)** 40
Figure S74. ^{31}P NMR spectrum of [8,8'-$O_2P(O)S(CH_2)_4OCH(CH_2OTr)_2$-3,3'-Co(1,2-$C_2B_9H_{10})_2$]$HNEt_3$ **(21)** 40
Figure S75. MS (ESI) spectrum of [8,8'-$O_2P(O)S(CH_2)_4OCH(CH_2OTr)_2$-3,3'-Co(1,2-$C_2B_9H_{10})_2$]$HNEt_3$ **(21)** 41
Figure S76. MS (ESI) spectrum of [8,8'-$O_2P(O)S(CH_2)_4OCH(CH_2OTr)_2$-3,3'-Co(1,2-$C_2B_9H_{10})_2$]$HNEt_3$ **(21)** 41
Figure S77. UV-VIS spectrum of spectrum of [8,8'-$O_2P(O)S(CH_2)_4OCH(CH_2OTr)_2$-3,3'-Co(1,2-$C_2B_9H_{10})_2$]$HNEt_3$ **(21)** 41

Figure S78. ^{1}H NMR spectrum of {8,8'-$O_2P(O)S[(CH_2)_4OCH(CH_2OCPh_3)_2]$-3,3'-Co[1-$(CH_2)_2OH$-1,2-$C_2B_9H_{10}$)](1',2'-$C_2B_9H_{10}$)} **(22)**.. 42
Figure S79. $^{11}B\{^{1}H\}$ NMR spectrum of {8,8'-$O_2P(O)S[(CH_2)_4OCH(CH_2OCPh_3)_2]$-3,3'-Co[1-$(CH_2)_2OH$-1,2-$C_2B_9H_{10}$)](1',2'-$C_2B_9H_{10}$)} **(22)**.. 42
Figure S80. ^{11}B NMR spectrum of {8,8'-$O_2P(O)S[(CH_2)_4OCH(CH_2OCPh_3)_2]$-3,3'-Co[1-$(CH_2)_2OH$-1,2-$C_2B_9H_{10}$)](1',2'-$C_2B_9H_{10}$)} **(22)**.. 43
Figure S81. $^{31}P\{^{1}H\}$ NMR spectrum of {8,8'-$O_2P(O)S[(CH_2)_4OCH(CH_2OCPh_3)_2]$-3,3'-Co[1-$(CH_2)_2OH$-1,2-$C_2B_9H_{10}$)](1',2'-$C_2B_9H_{10}$)} **(22)**.. 43
Figure S82. ^{31}P NMR spectrum of {8,8'-$O_2P(O)S[(CH_2)_4OCH(CH_2OCPh_3)_2]$-3,3'-Co[1-$(CH_2)_2OH$-1,2-$C_2B_9H_{10}$)](1',2'-$C_2B_9H_{10}$)} **(22)**.. 44
Figure S83. MS (ESI) spectrum of {8,8'-$O_2P(O)S[(CH_2)_4OCH(CH_2OCPh_3)_2]$-3,3'-Co[1-$(CH_2)_2OH$-1,2-$C_2B_9H_{10}$)](1',2'-$C_2B_9H_{10}$)} **(22)**.. 44
Figure S84. FT-IR spectrum of {8,8'-$O_2P(O)S[(CH_2)_4OCH(CH_2OCPh_3)_2]$-3,3'-Co[1-$(CH_2)_2OH$-1,2-$C_2B_9H_{10}$)](1',2'-$C_2B_9H_{10}$)} **(22)**.. 44
Figure S85. ^{1}H NMR spectrum of {8,8'-$O_2P(O)S[(CH_2)_4OCH(CH_2OCPh_3)_2]$-3,3'-Co[1-$(CH_2)_2OH$-1,2-$C_2B_9H_{10}$)] [1'-$(CH_2)_2OH$-1',2'-$C_2B_9H_{10}$)]} $HNEt_3$ **(23)**.................................. 45
Figure S86. $^{11}B\{^{1}H\}$ NMR spectrum of {8,8'-$O_2P(O)S[(CH_2)_4OCH(CH_2OCPh_3)_2]$-3,3'-Co[1-$(CH_2)_2OH$-1,2-$C_2B_9H_{10}$)] [1'-$(CH_2)_2OH$-1',2'-$C_2B_9H_{10}$)]} $HNEt_3$ **(23)**.................................. 45
Figure S87. ^{11}B NMR spectrum of {8,8'-$O_2P(O)S[(CH_2)_4OCH(CH_2OCPh_3)_2]$-3,3'-Co[1-$(CH_2)_2OH$-1,2-$C_2B_9H_{10}$)] [1'-$(CH_2)_2OH$-1',2'-$C_2B_9H_{10}$)]} $HNEt_3$ **(23)**.................................. 46
Figure S88. $^{31}P\{^{1}H\}$ NMR spectrum of {8,8'-$O_2P(O)S[(CH_2)_4OCH(CH_2OCPh_3)_2]$-3,3'-Co[1-$(CH_2)_2OH$-1,2-$C_2B_9H_{10}$)] [1'-$(CH_2)_2OH$-1',2'-$C_2B_9H_{10}$)]} $HNEt_3$ **(23)**.................................. 46
Figure S89. ^{31}P NMR spectrum of {8,8'-$O_2P(O)S[(CH_2)_4OCH(CH_2OCPh_3)_2]$-3,3'-Co[1-$(CH_2)_2OH$-1,2-$C_2B_9H_{10}$)] [1'-$(CH_2)_2OH$-1',2'-$C_2B_9H_{10}$)]} **$HNEt_3$ (23)** 47
Figure S90. ESI (MS) spectrum of {8,8'-$O_2P(O)S[(CH_2)_4OCH(CH_2OCPh_3)_2]$-3,3'-Co[1-$(CH_2)_2OH$-1,2-$C_2B_9H_{10}$)] [1'-$(CH_2)_2OH$-1',2'-$C_2B_9H_{10}$)]} $HNEt_3$ **(23)**.................................. 47
Figure S91. FT-IR spectrum of {8,8'-$O_2P(O)S[(CH_2)_4OCH(CH_2OCPh_3)_2]$-3,3'-Co[1-$(CH_2)_2OH$-1,2-$C_2B_9H_{10}$)] [1'-$(CH_2)_2OH$-1',2'-$C_2B_9H_{10}$)]} $HNEt_3$ **(23)**.................................. 47

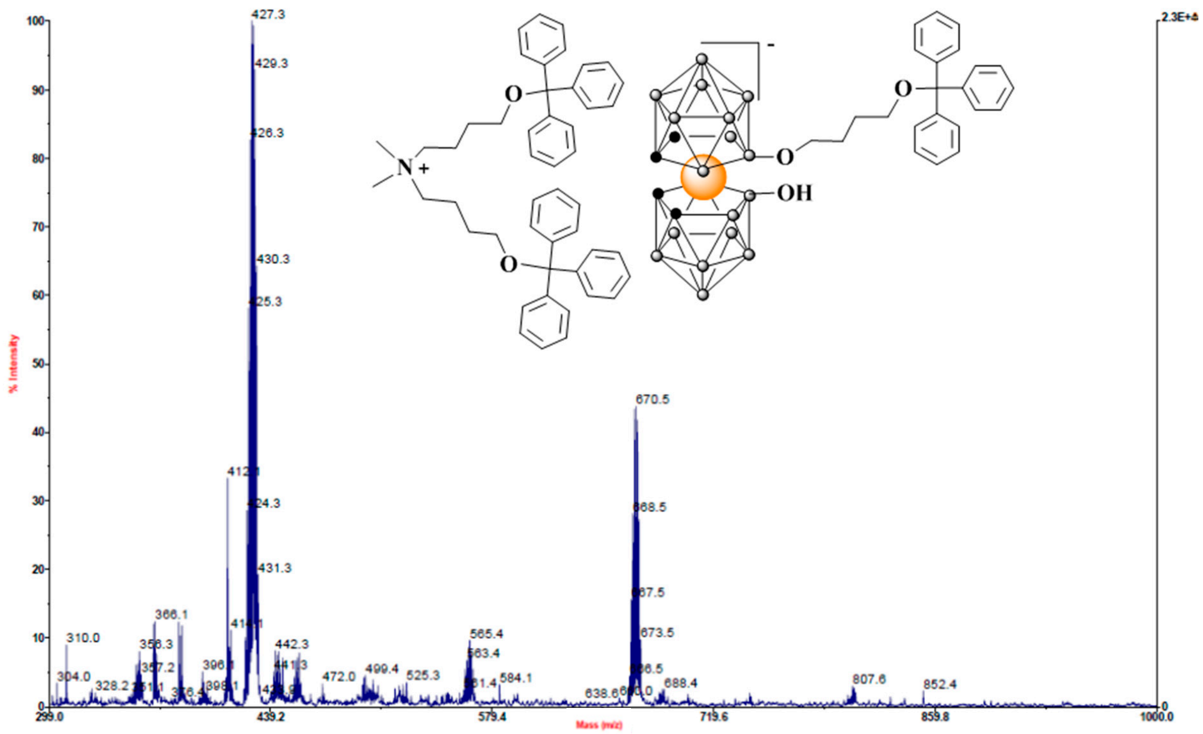

Figure S1. MS (ESI) spectrum of 3,3'-Co{[(8-O$(CH_2)_4$OCPh$_3$]-1,2-$C_2B_9H_{10}$}(8'-OH-1',2'-$C_2B_9H_{10}$)$(CH_3)_2$N[$(CH_2)_4$OPh$_3$]$_2$ **(5)**.

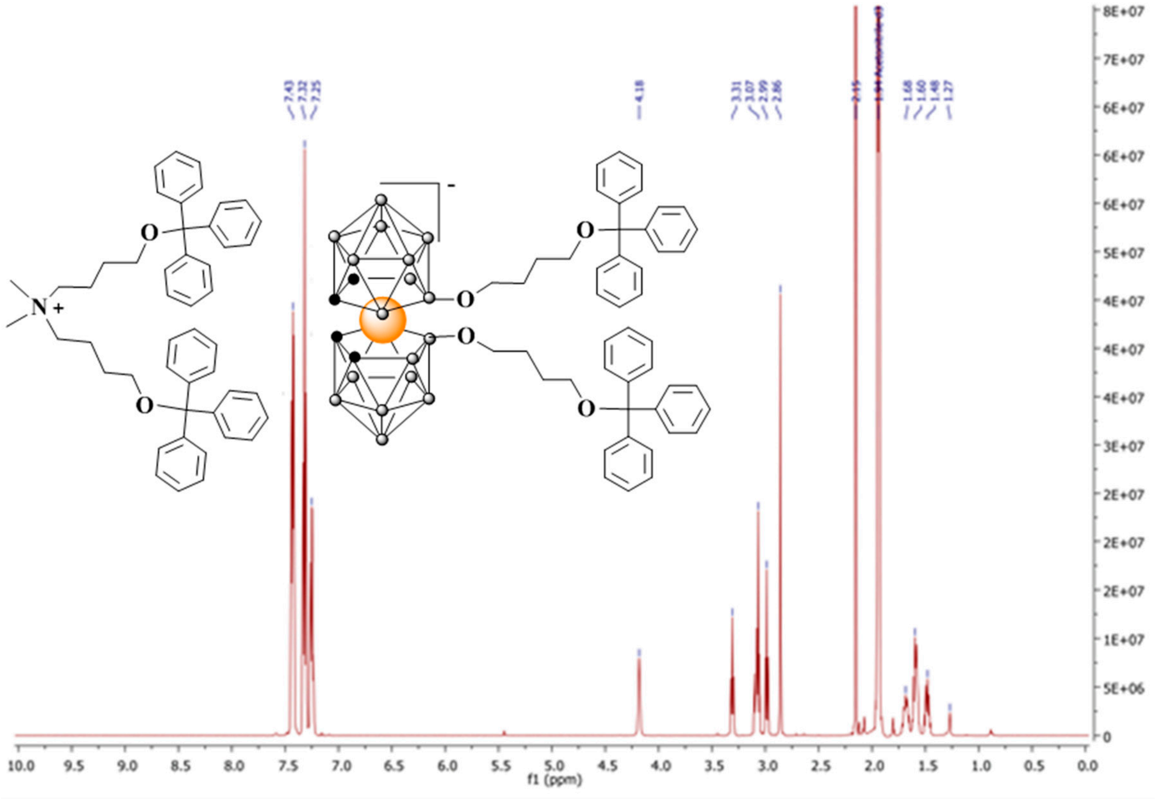

Figure S2. ^{1}H NMR spectrum of 3,3'-Co[(8-O$(CH_2)_4$OCPh$_3$-1,2-$C_2B_9H_{10}$)]$_2$ $(CH_3)_2$N[$(CH_2)_4$OPh$_3$]$_2$ **(7)**.

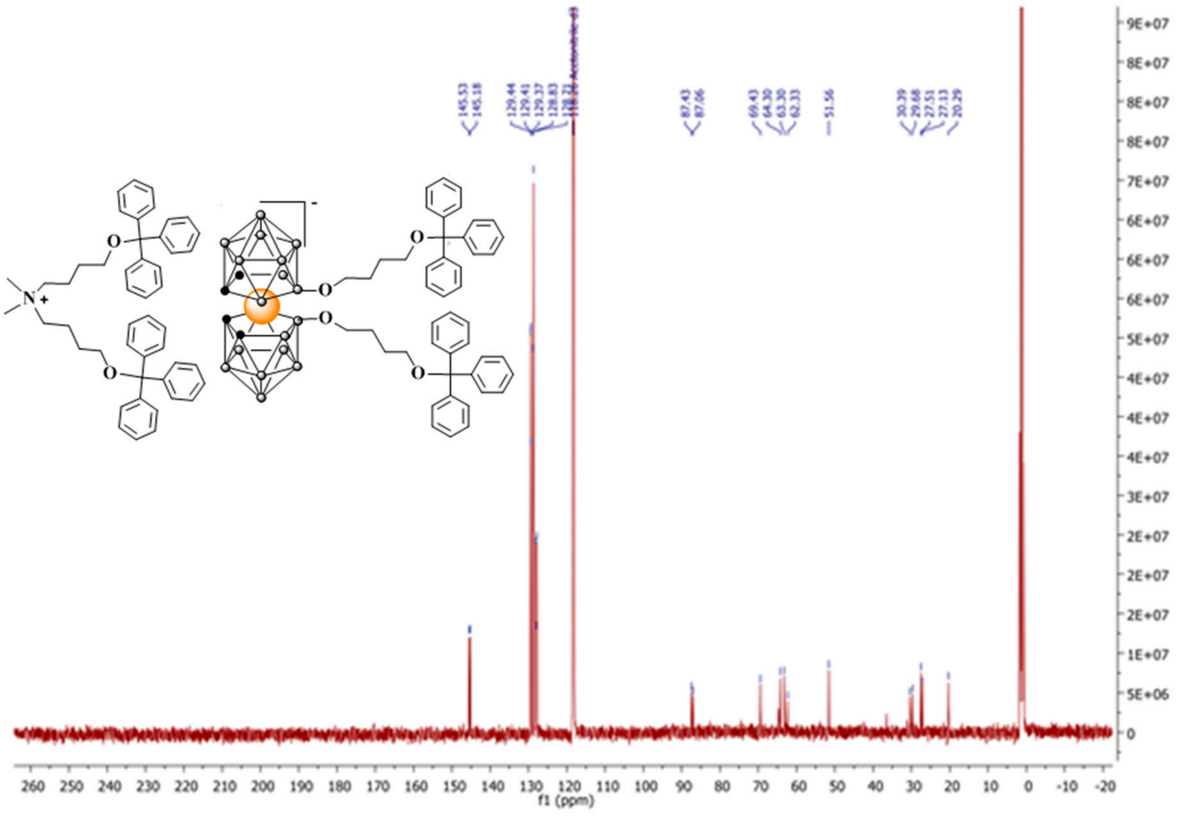

Figure S3. $^{13}C\{^{1}H\}$ NMR spectrum of 3,3'-Co[(8-O$(CH_2)_4OCPh_3$-1,2-$C_2B_9H_{10}$)]$_2$ $(CH_3)_2N[(CH_2)_4OPh_3]_2$ **(7)**.

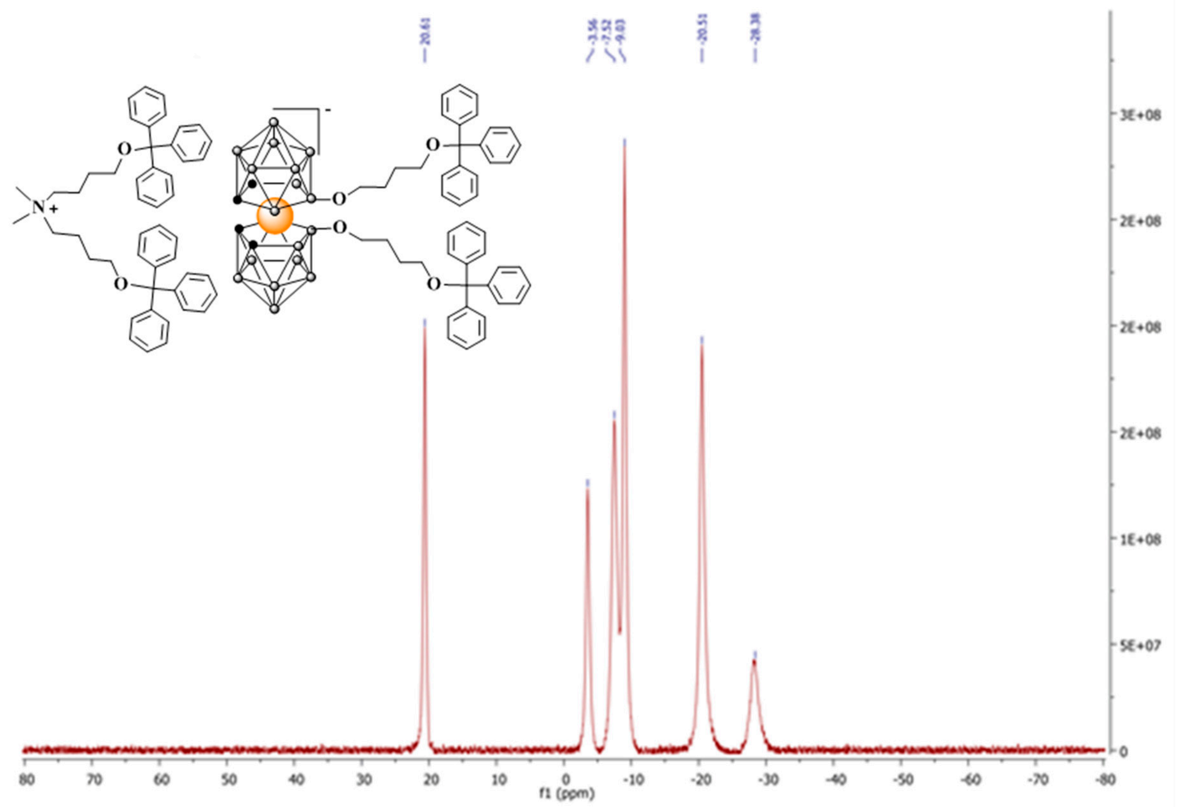

Figure S4. $^{11}B\{^{1}H\}$ NMR spectrum of 3,3'-Co[(8-O$(CH_2)_4OCPh_3$-1,2-$C_2B_9H_{10}$)]$_2$ $(CH_3)_2N[(CH_2)_4OPh_3]_2$ **(7)**.

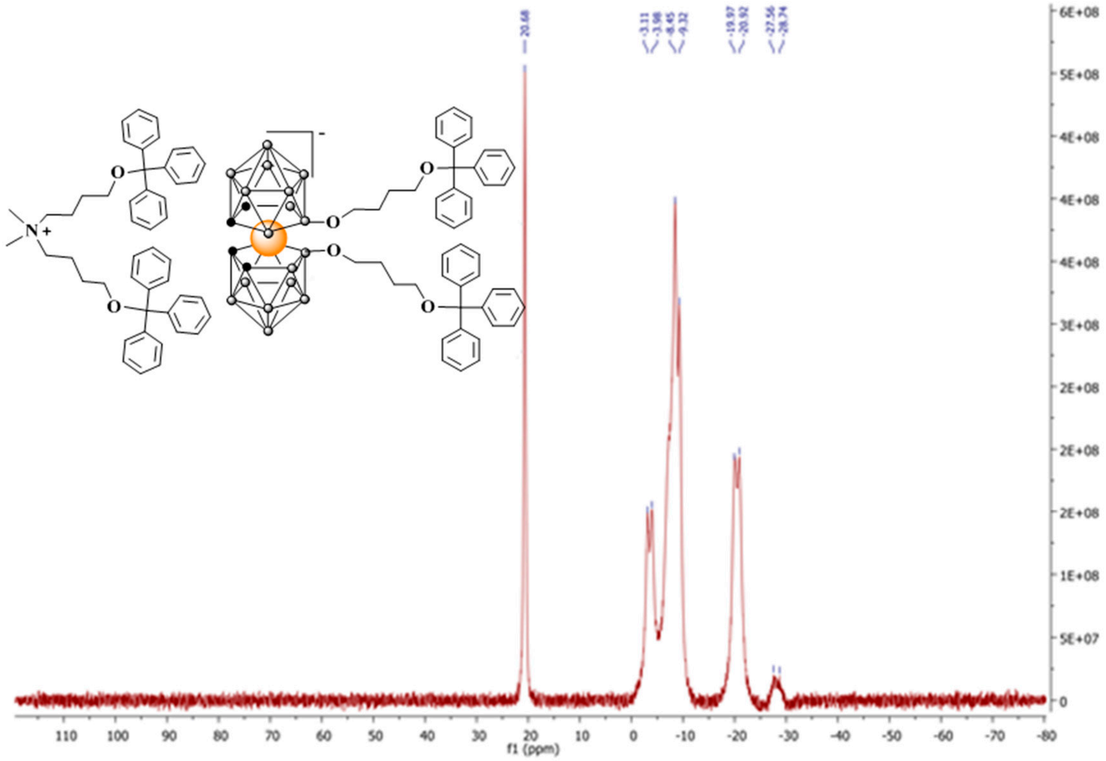

Figure S5. ^{11}B NMR spectrum of 3,3'-Co[(8-O(CH$_2$)$_4$OCPh$_3$-1,2-C$_2$B$_9$H$_{10}$)]$_2$ (CH$_3$)$_2$N[(CH$_2$)$_4$OPh$_3$]$_2$ **(7)**.

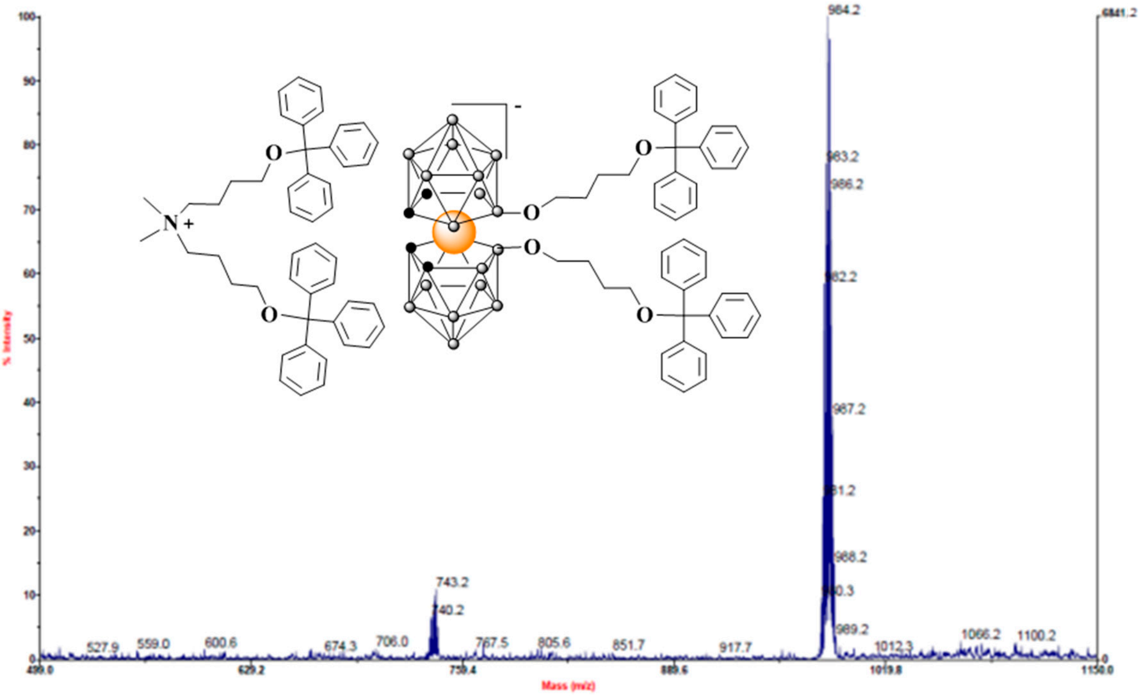

Figure S6. MS (ESI) spectrum of 3,3'-Co[(8-O(CH$_2$)$_4$OCPh$_3$-1,2-C$_2$B$_9$H$_{10}$)]$_2$ (CH$_3$)$_2$N[(CH$_2$)$_4$OPh$_3$]$_2$ **(7)**.

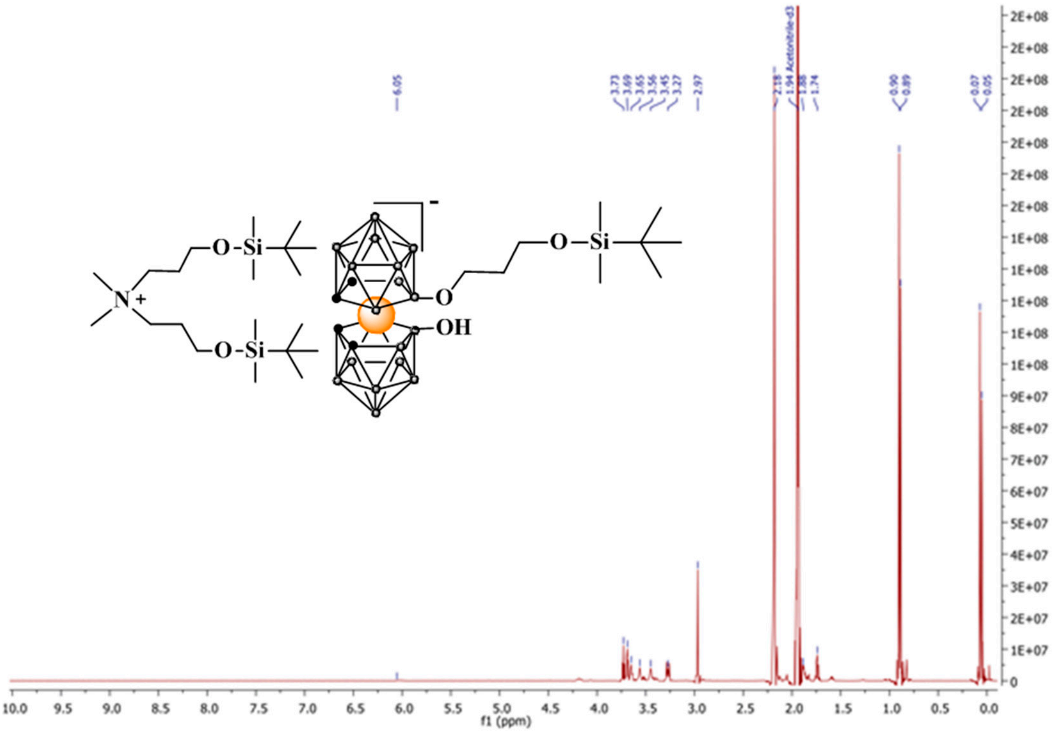

Figure S7. ^{1}H NMR spectrum of 3,3'-Co[8-O(CH_2)$_3$OTBDMS]-1,2-$C_2B_9H_{10}$)(8'-OH-1,2-$C_2B_9H_{10}$) $(CH_3)_2N[(CH_2)_3OTBDMS)]_2$ **(6)**.

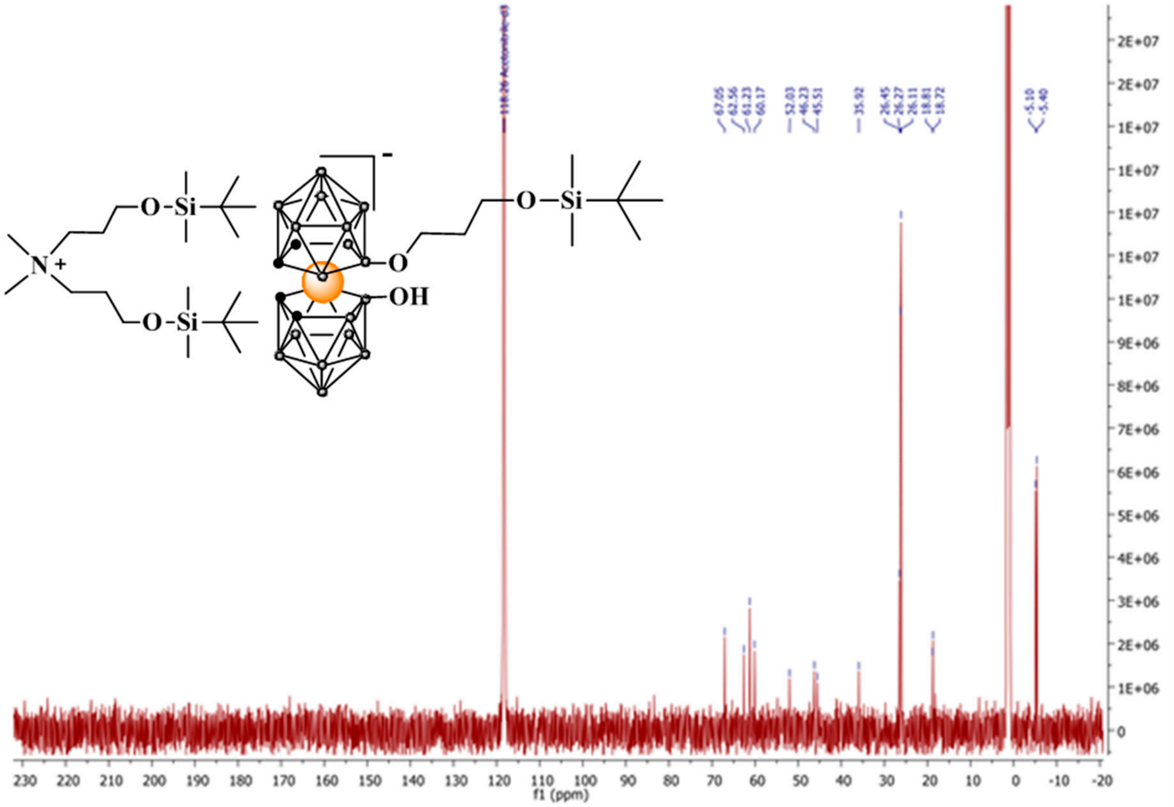

Figure S8. $^{13}C\{^{1}H\}$ NMR spectrum of 3,3'-Co[8-O(CH_2)$_3$OTBDMS]-1,2-$C_2B_9H_{10}$)(8'-OH-1,2-$C_2B_9H_{10}$) $(CH_3)_2N[(CH_2)_3OTBDMS)]_2$ **(6)**.

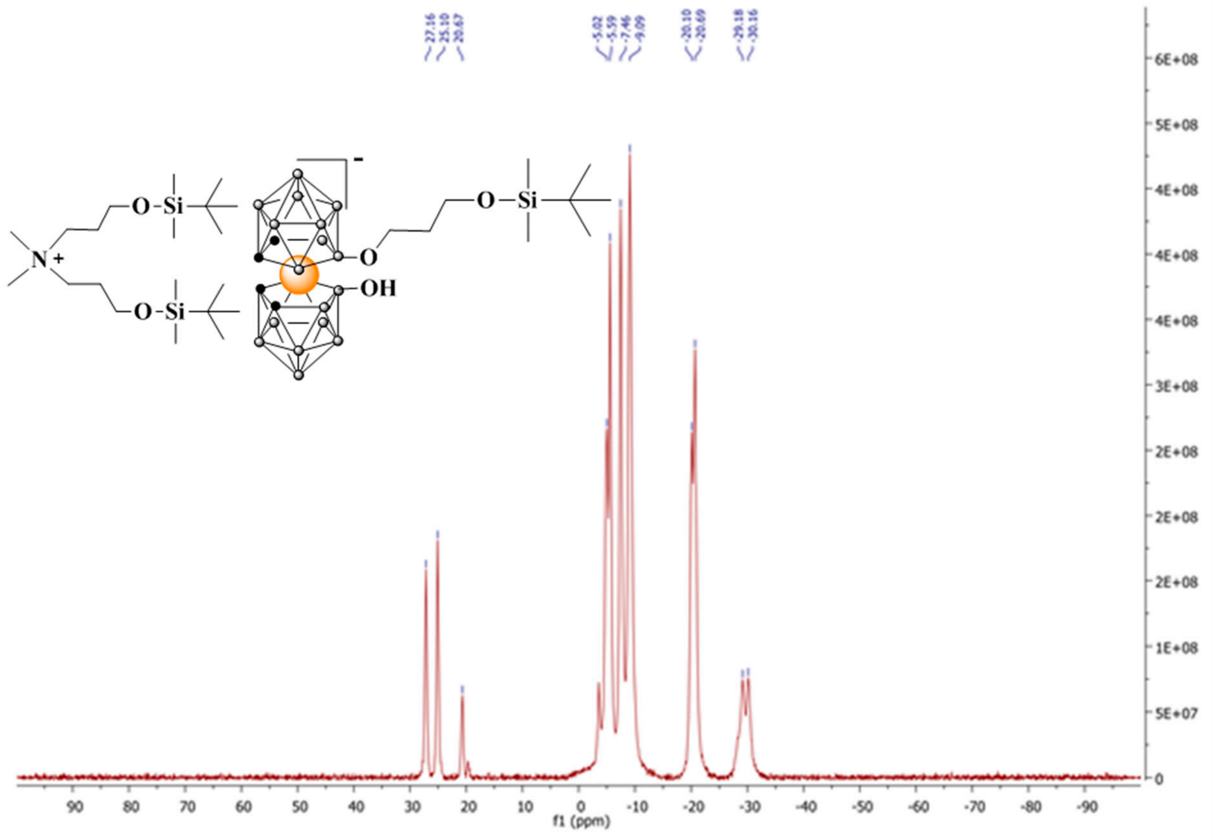

Figure S9. $^{11}B\{^1H\}$ NMR spectrum of 3,3'-Co[8-O$(CH_2)_3$OTBDMS]-1,2-$C_2B_9H_{10}$)(8'-OH-1,2-$C_2B_9H_{10}$) $(CH_3)_2N[(CH_2)_3OTBDMS)]_2$ **(6)**.

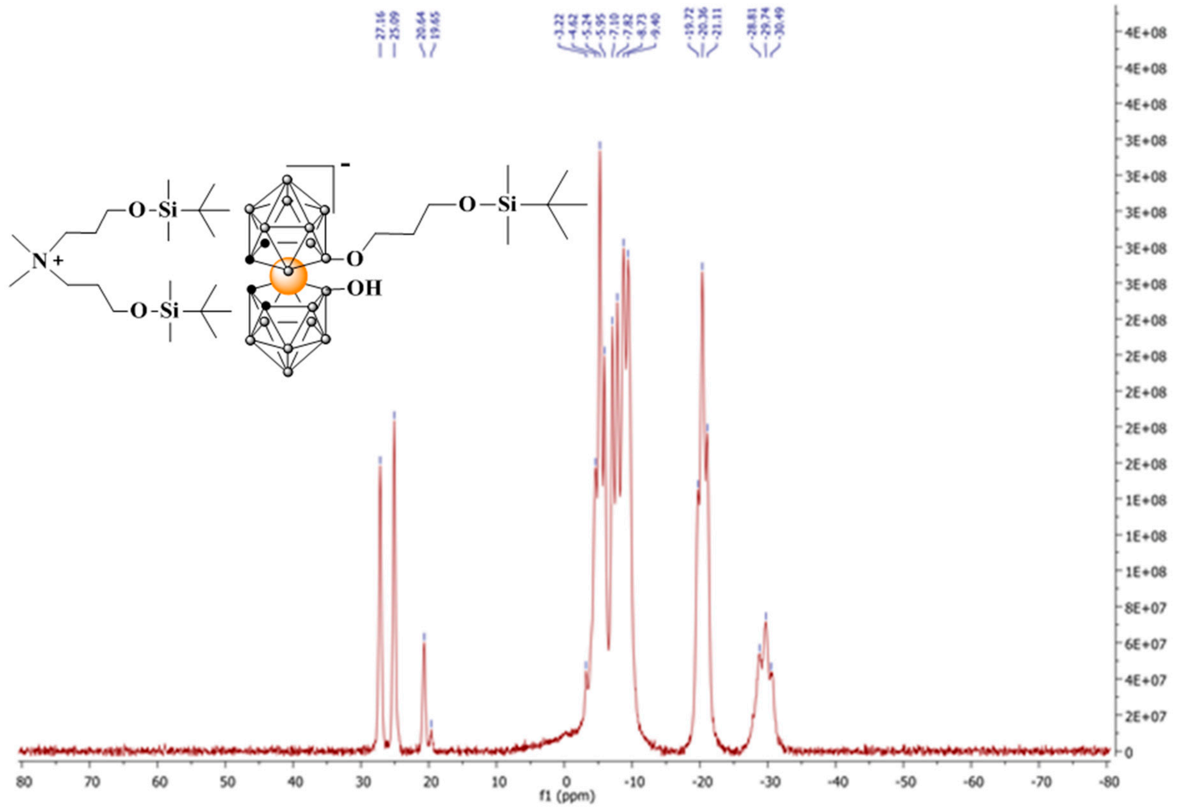

Figure S10. ^{11}B NMR spectrum of 3,3'-Co[8-O$(CH_2)_3$OTBDMS]-1,2-$C_2B_9H_{10}$)(8'-OH-1,2-$C_2B_9H_{10}$) $(CH_3)_2N[(CH_2)_3OTBDMS)]_2$ **(6)**.

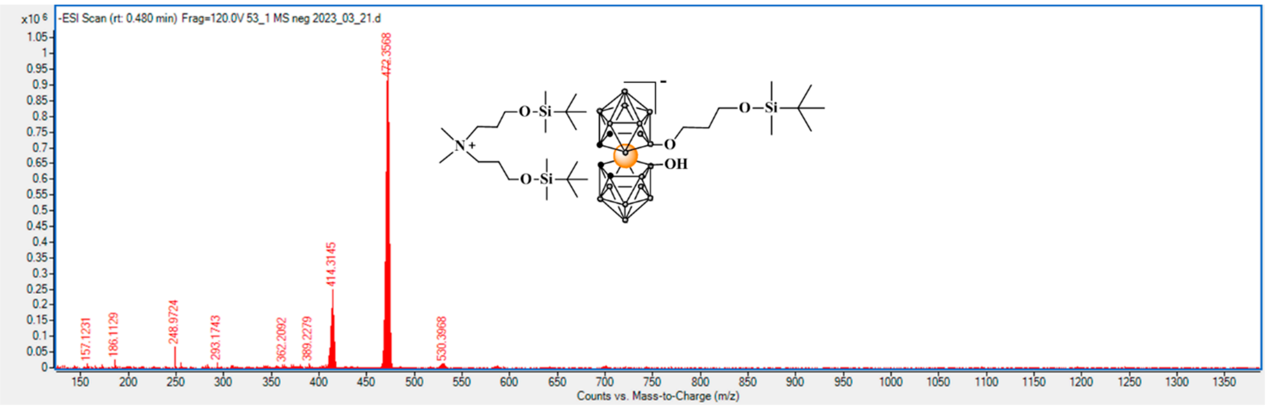

Figure S11. MS(ESI) spectrum of 3,3'-Co[8-O$(CH_2)_3$OTBDMS]-1,2-$C_2B_9H_{10}$)(8'-OH-1,2-$C_2B_9H_{10}$) $(CH_3)_2N[(CH_2)_3OTBDMS)]_2$ **(6)**.

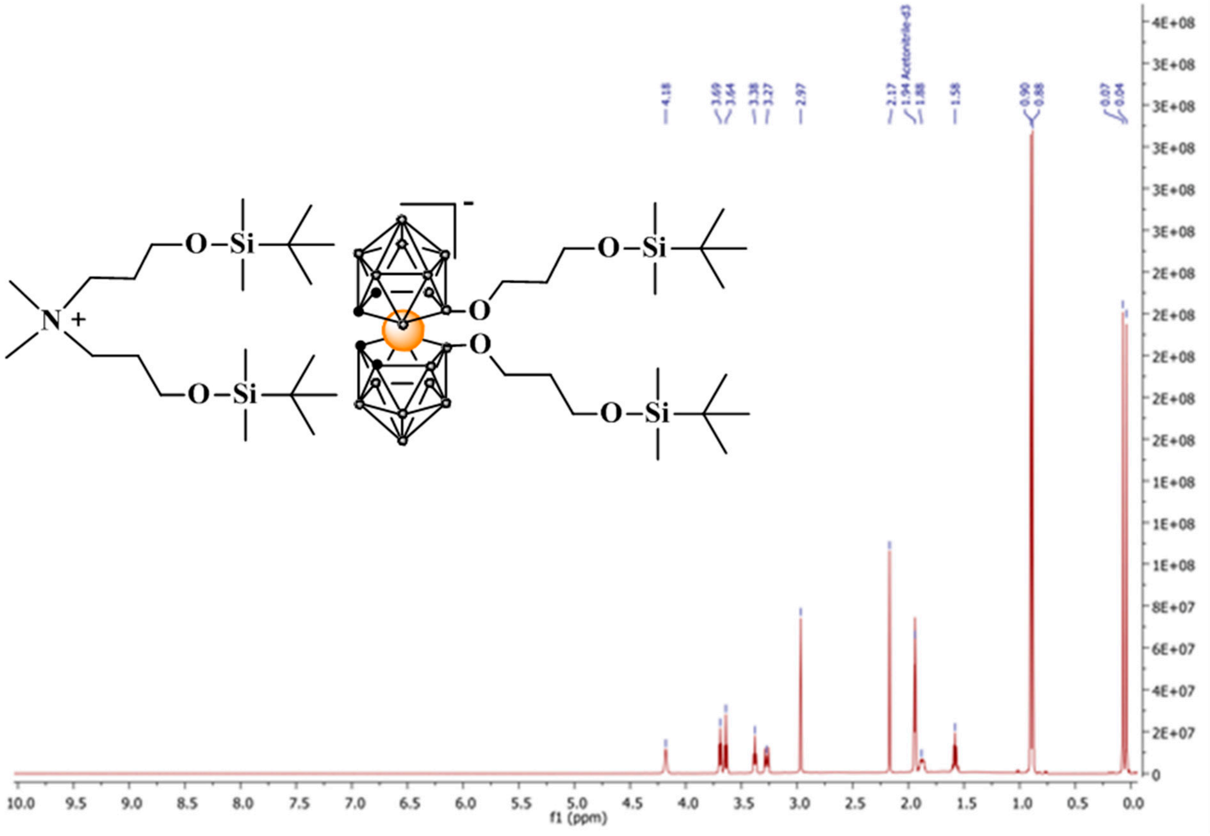

Figure S12. ^{1}H NMR spectrum 3,3'-Co[8-O$(CH_2)_3$OTBDMS-1,2-$C_2B_9H_{10}$)$]_2$ $(CH_3)_2N[(CH_2)_3OTBDMS)]_2$ (**8**).

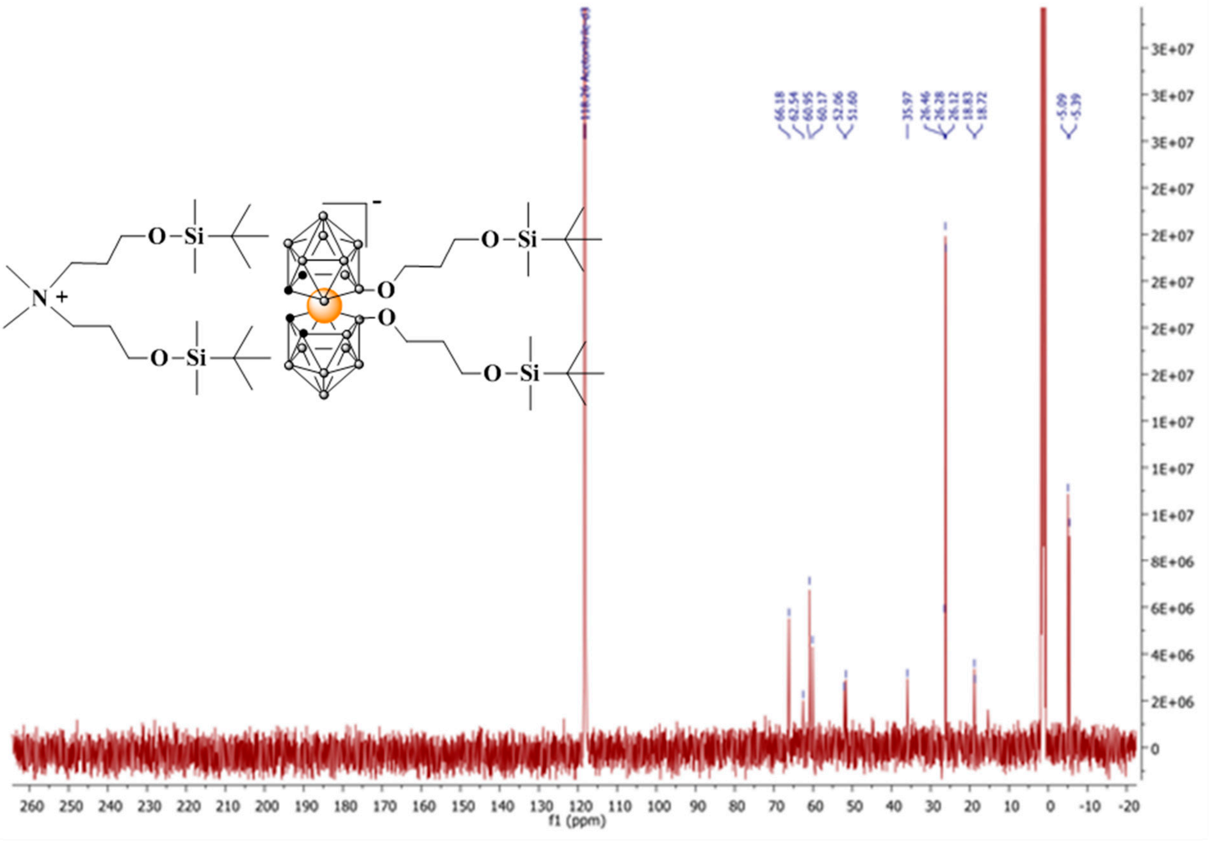

Figure S13. $^{13}C\{^1H\}$ NMR spectrum of 3,3’-Co[8-O$(CH_2)_3$OTBDMS-1,2-$C_2B_9H_{10}$)]$_2$ $(CH_3)_2N[(CH_2)_3OTBDMS)]_2$ **(8).**

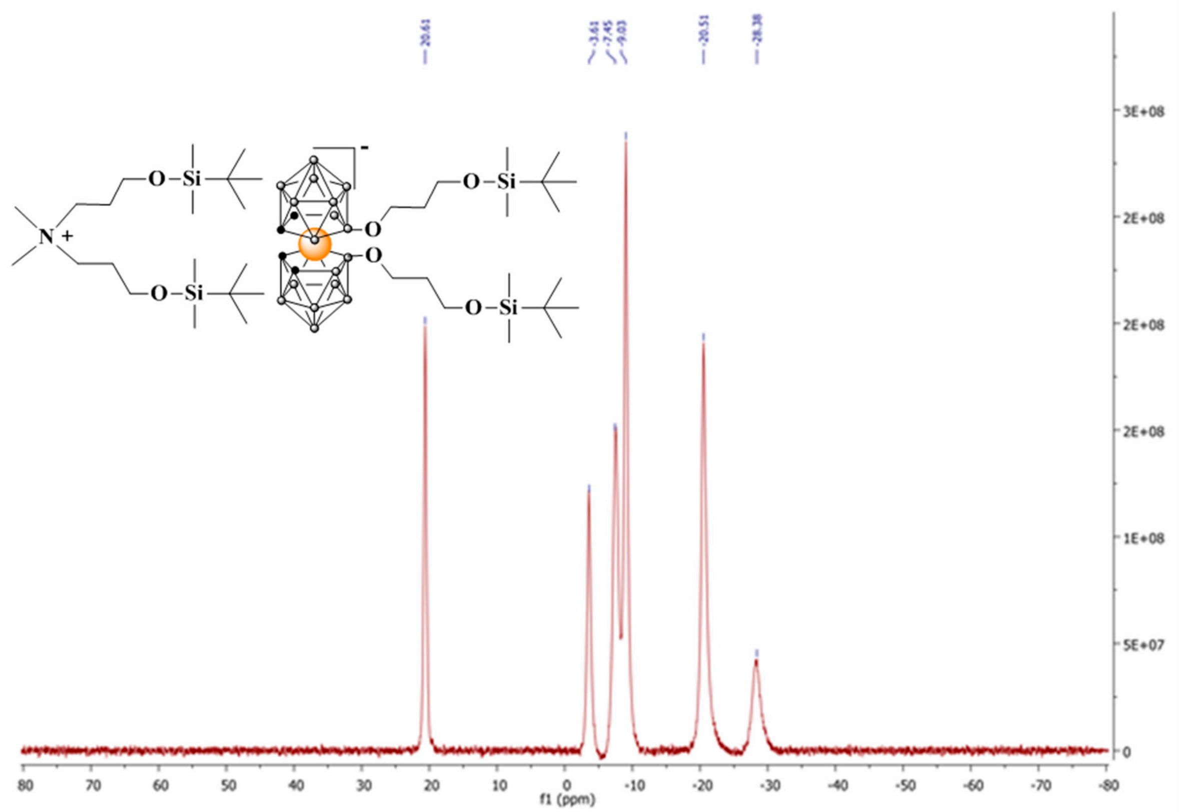

Figure S14. $^{11}B\{^1H\}$ NMR spectrum of 3,3’-Co[8-O$(CH_2)_3$OTBDMS-1,2-$C_2B_9H_{10}$)]$_2$ $(CH_3)_2N[(CH_2)_3OTBDMS)]_2$ **(8).**

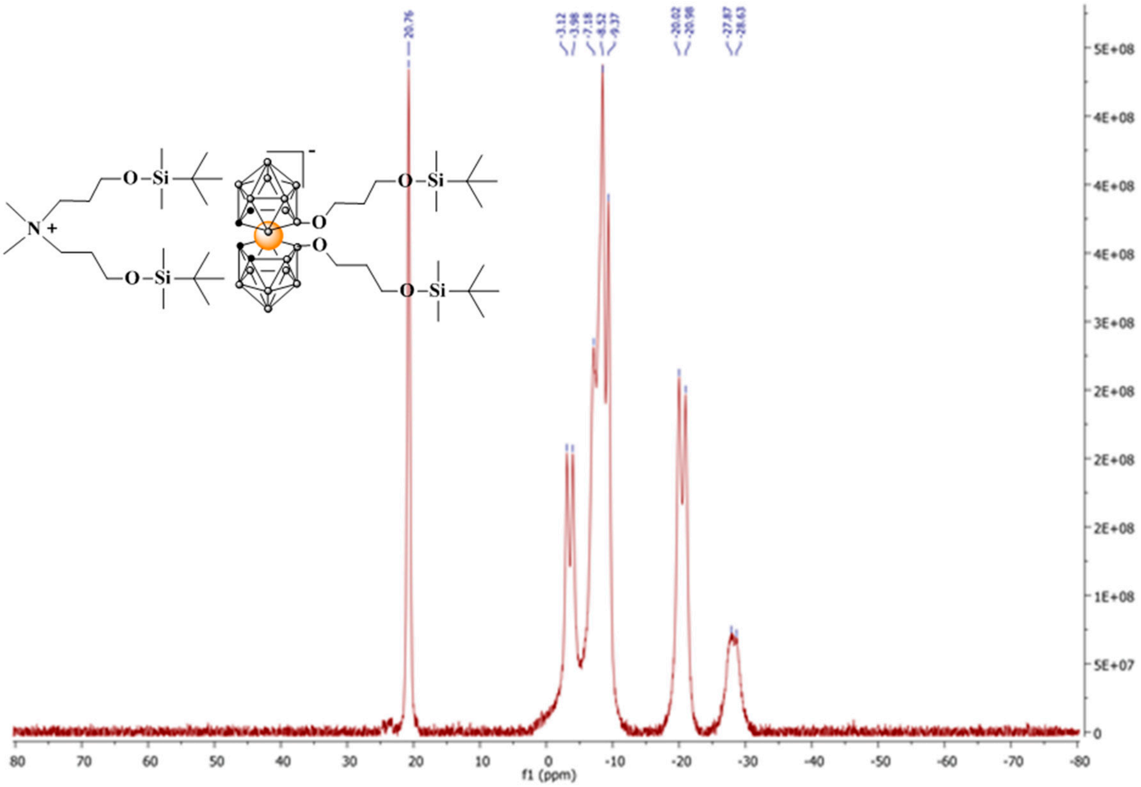

Figure S15. ^{11}B NMR spectrum of 3,3'-Co[8-O$(CH_2)_3$OTBDMS-1,2-$C_2B_9H_{10}$)$]_2$ $(CH_3)_2$N[$(CH_2)_3$OTBDMS)$]_2$ **(8).**

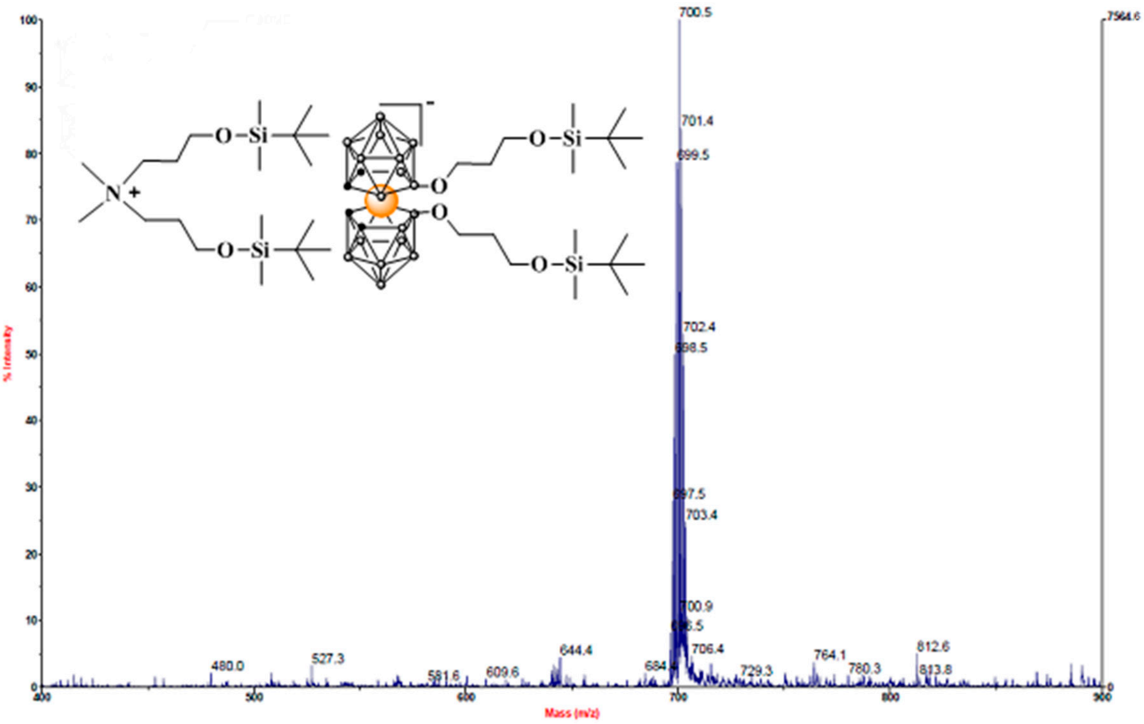

Figure S16. MS(ESI) spectrum of 3,3'-Co[8-O$(CH_2)_3$OTBDMS-1,2-$C_2B_9H_{10}$)$]_2$ $(CH_3)_2$N[$(CH_2)_3$OTBDMS)$]_2$ **(8).**

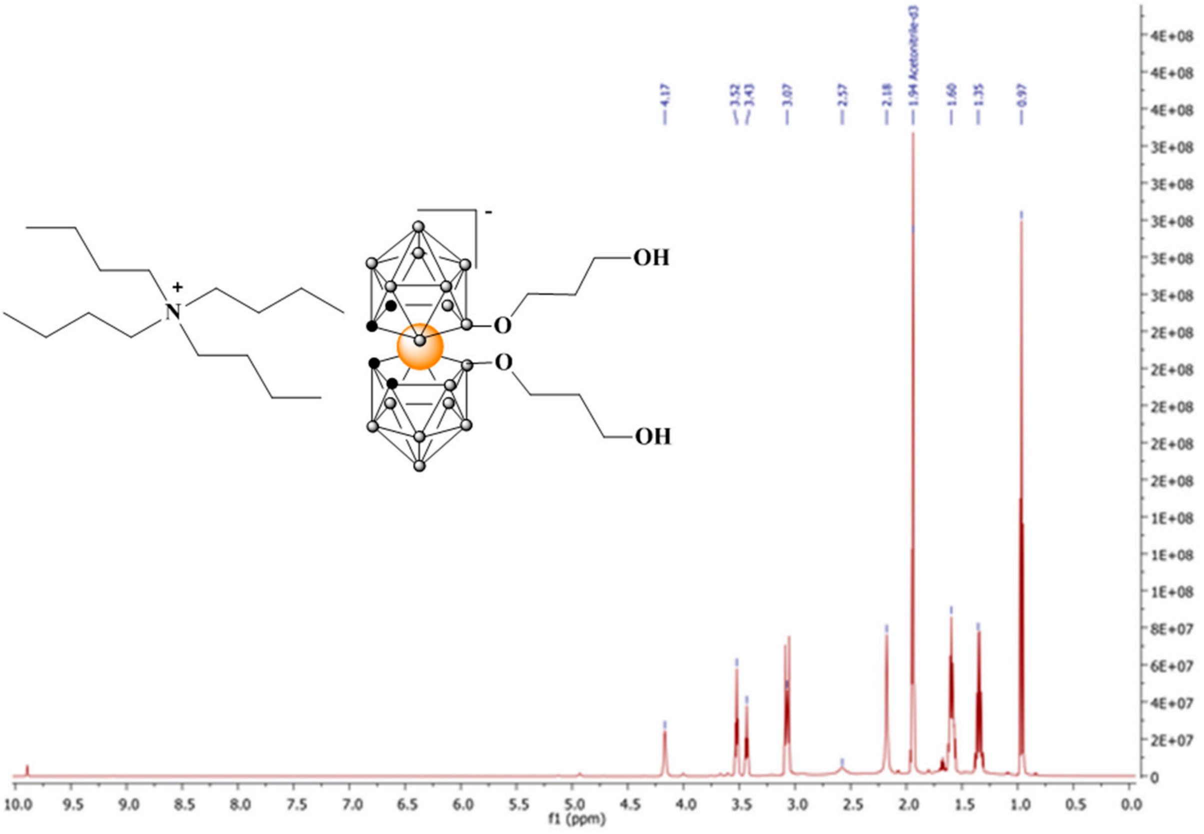

Figure S17. ^{1}H NMR spectrum of [3,3'-Co(8-O(CH$_2$)$_3$OH-1,2-C$_2$B$_9$H$_{10}$)$_2$] TBA **(9).**

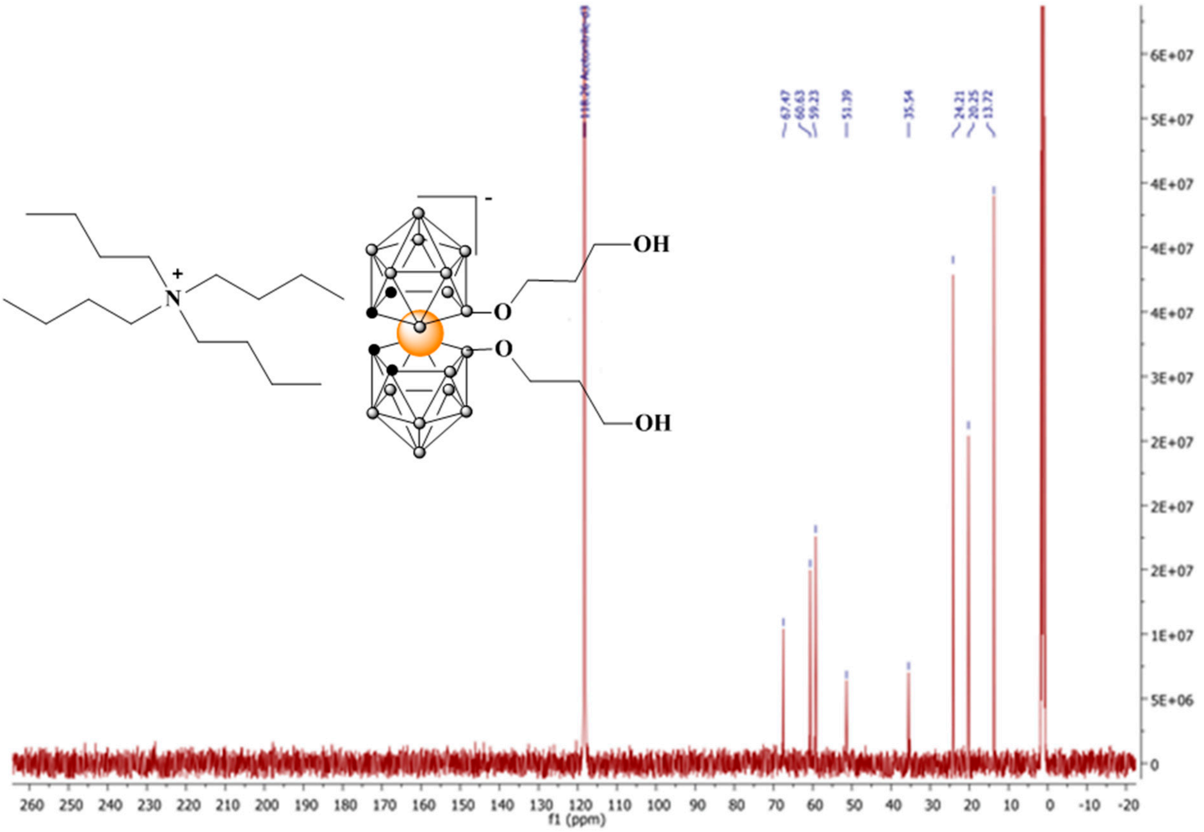

Figure S18. ^{13}C{^{1}H} NMR spectrum of [3,3'-Co(8-O(CH$_2$)$_3$OH-1,2-C$_2$B$_9$H$_{10}$)$_2$] TBA **(9).**

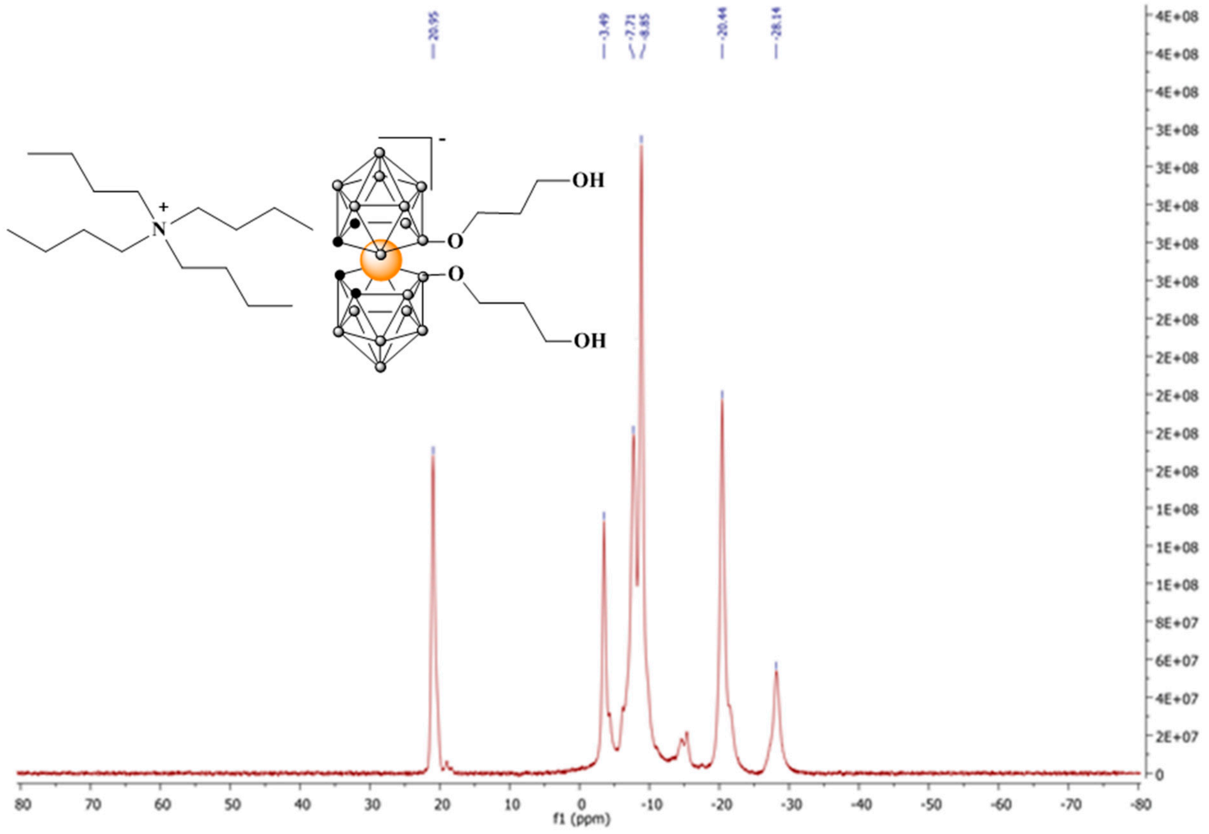

Figure S19. $^{11}B\{^{1}H\}$ NMR spectrum of [3,3'-Co(8-O(CH$_2$)$_3$OH-1,2-C$_2$B$_9$H$_{10}$)$_2$] TBA **(9).**

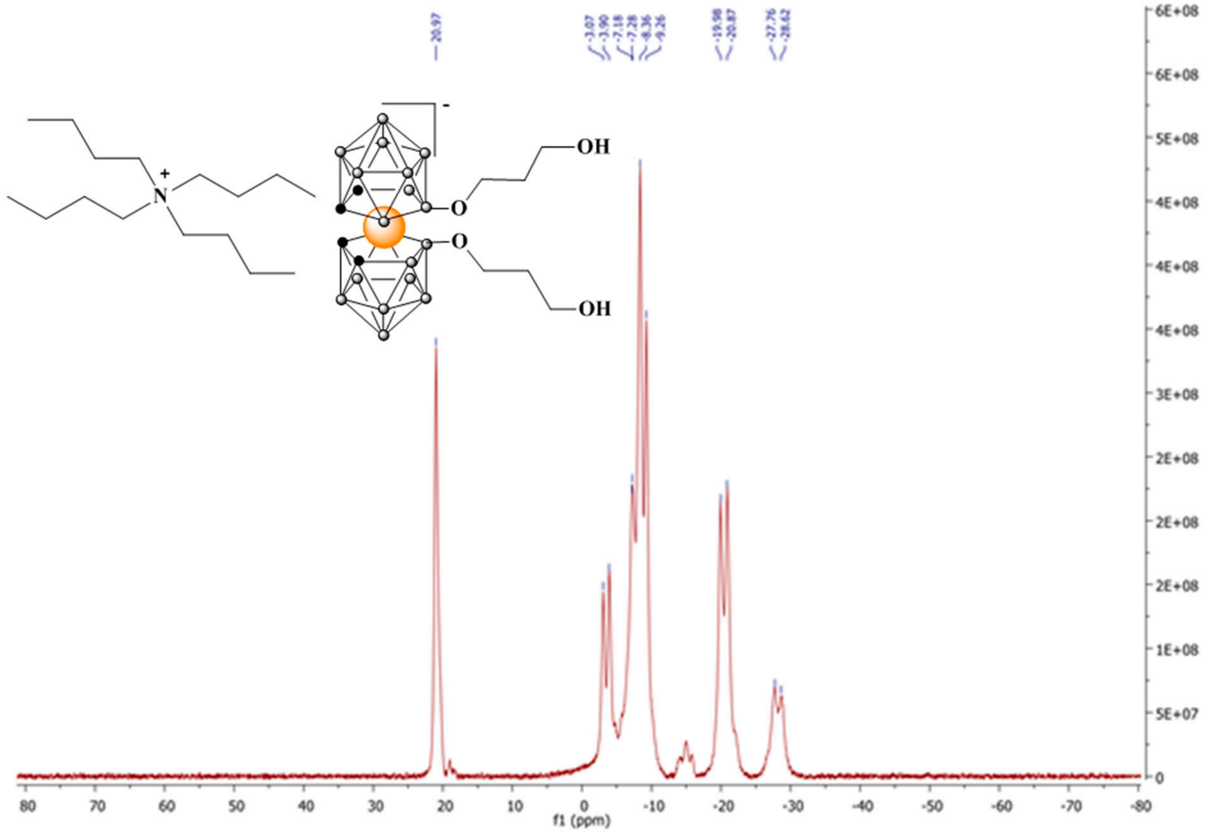

Figure S20. ^{11}B NMR spectrum of [3,3'-Co(8-O(CH$_2$)$_3$OH-1,2-C$_2$B$_9$H$_{10}$)$_2$] TBA **(9).**

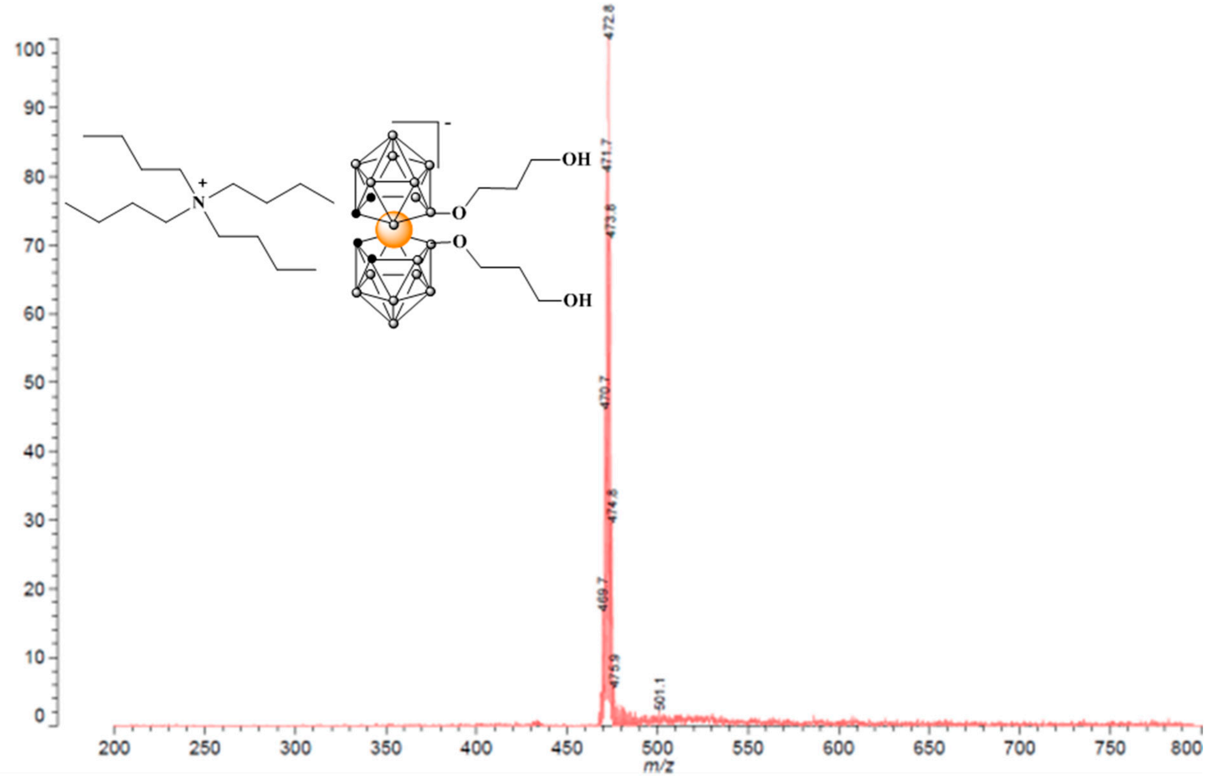

Figure S21. MS(ESI) spectrum of [3,3'-Co(8-O$(CH_2)_3$OH-1,2-$C_2B_9H_{10})_2$] TBA **(9).**

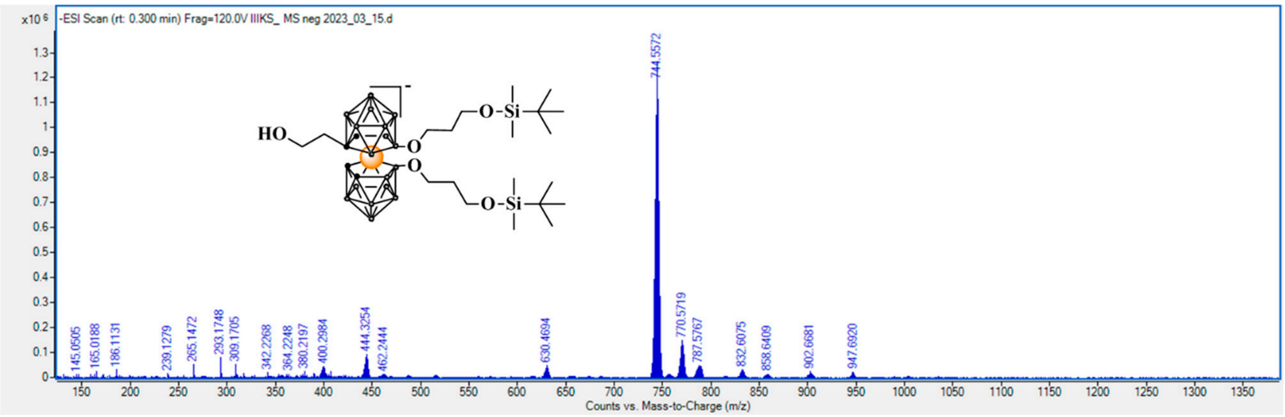

Figure S22. MS (ESI) spectrum of 3,3'-Co{[8-O$(CH_2)_3$OTBDMS-1-$(CH_2)_2$OH]-1,2-$C_2B_9H_9$)}[8'-O$(CH_2)_3$OTBDMS-1',2'-$C_2B_9H_{10}$)]$^-$ **(10)**.

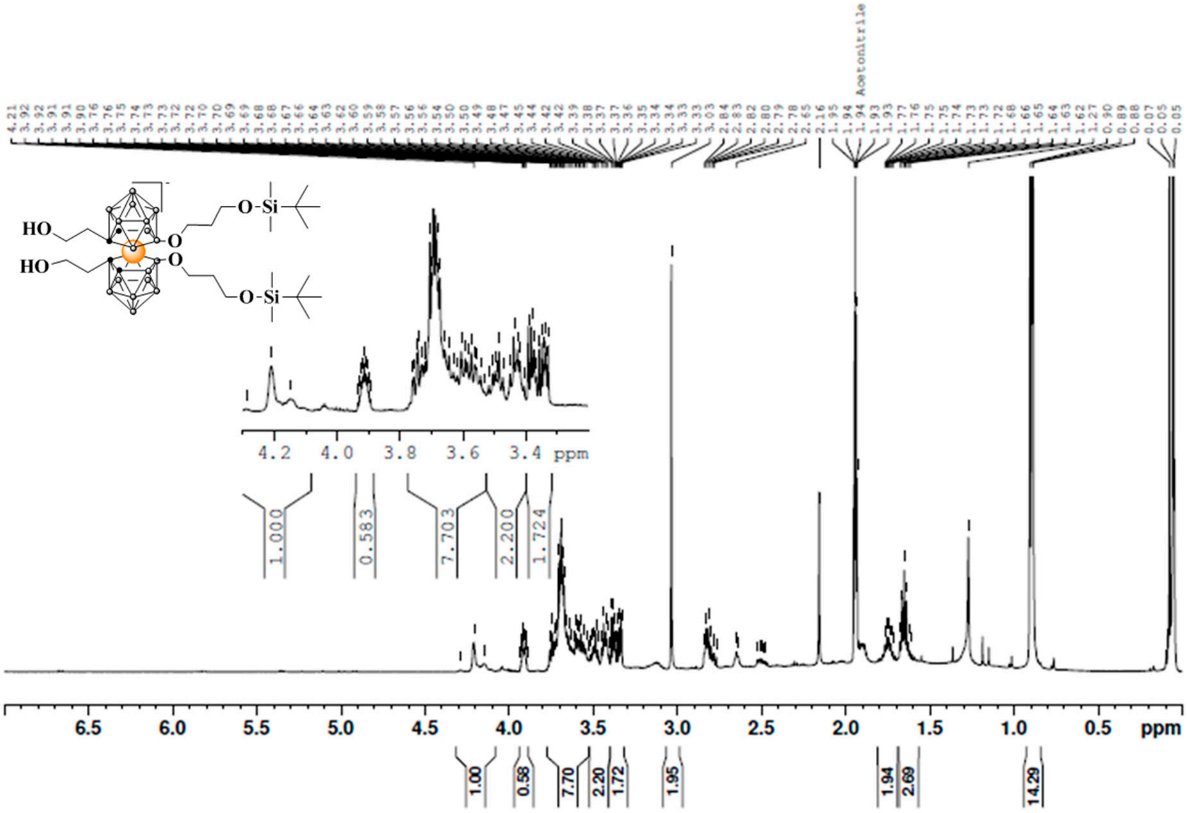

Figure S23. ^{1}H NMR spectrum of 3,3'-Co[(8-O(CH2)3OTBDMS-1-(CH2)2OH-1,2-C2B9H9)]2- **(11)**.

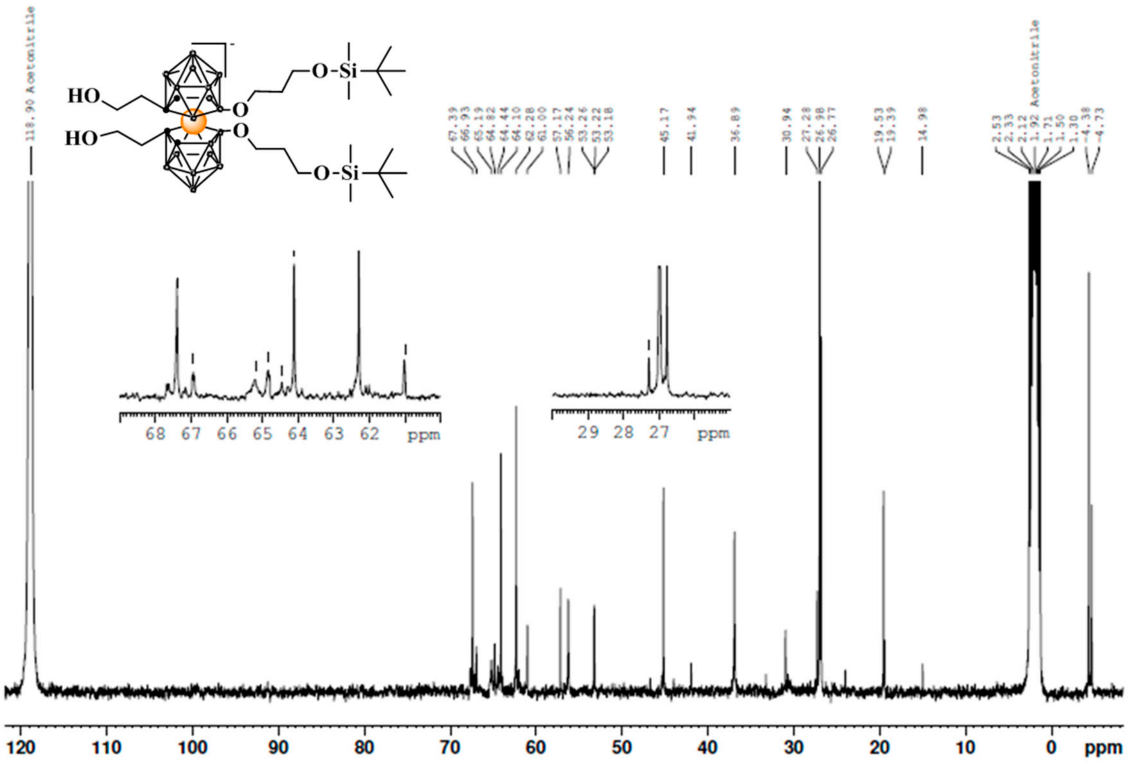

Figure S24. ^{13}C{^{1}H} NMR spectrum of 3,3'-Co[(8-O(CH2)3OTBDMS-1-(CH2)2OH-1,2-C2B9H9)]2- **(11)**.

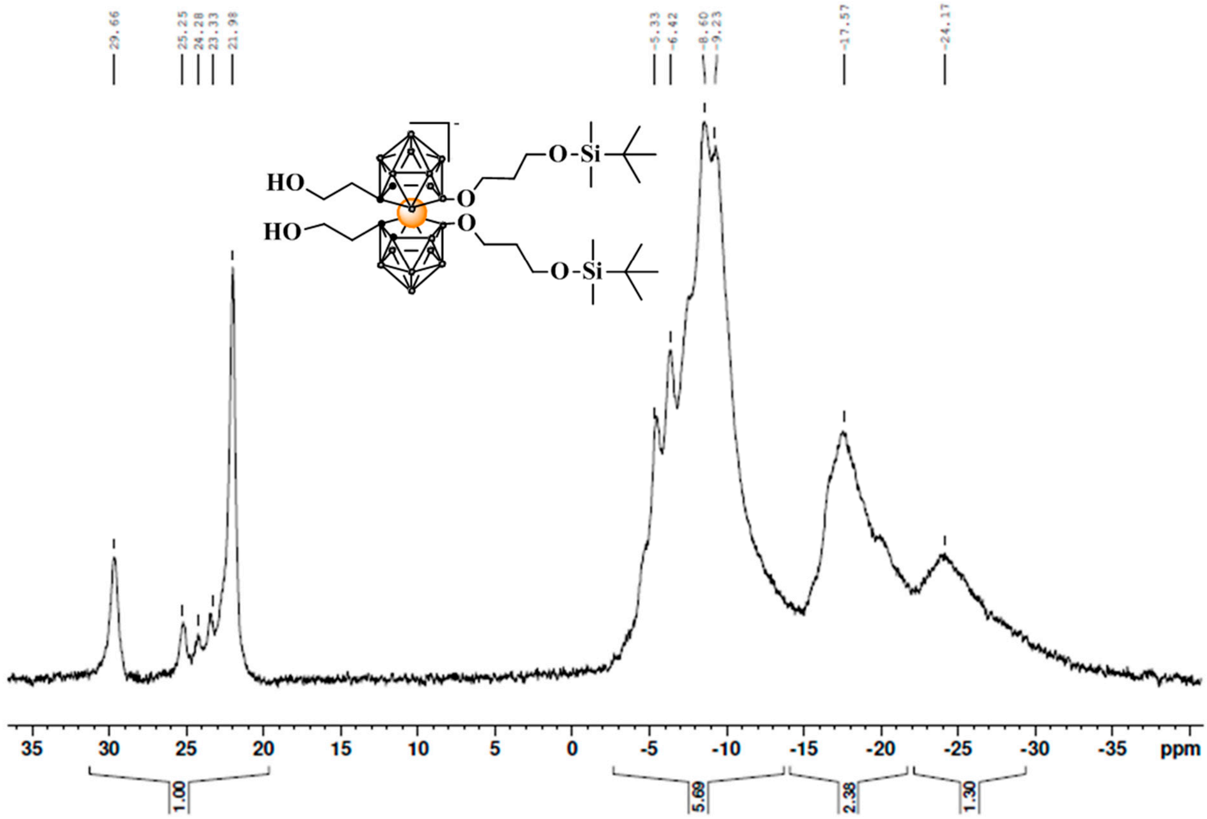

Figure S25. $^{11}B\{^{1}H\}$ NMR spectrum of 3,3'-Co[(8-O(CH$_2$)$_3$OTBDMS-1-(CH$_2$)$_2$OH-1,2-C$_2$B$_9$H$_9$)]$_2^-$ **(11)**.

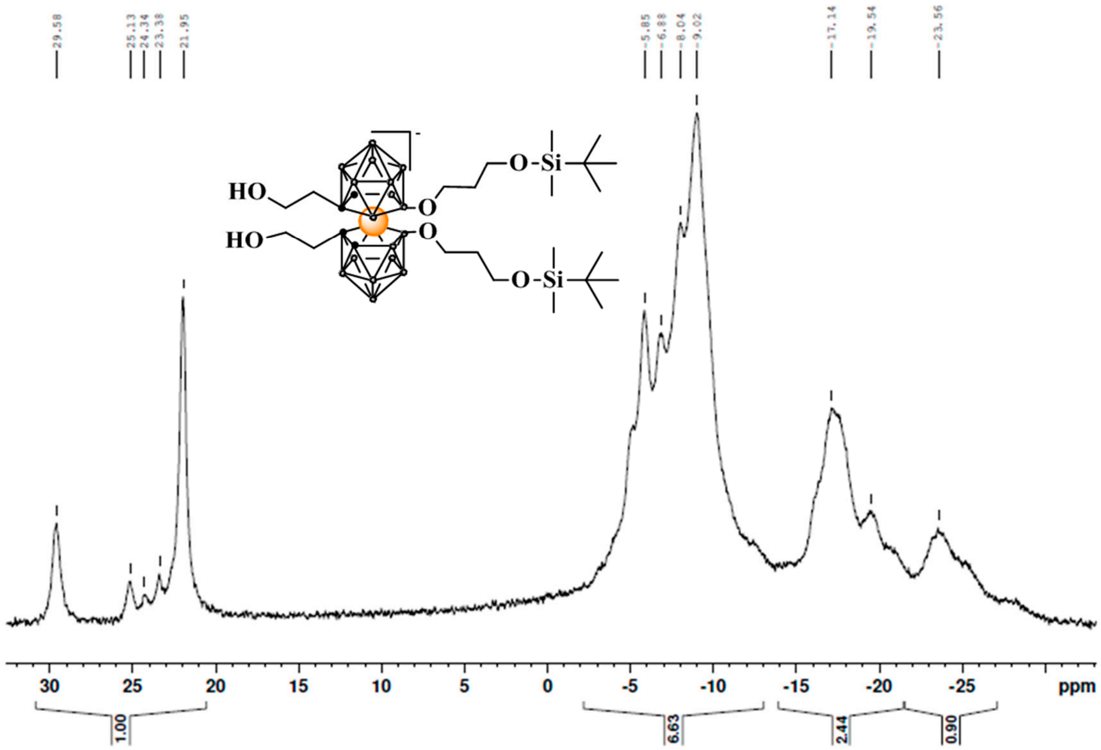

Figure S26. ^{11}B NMR spectrum of 3,3'-Co[(8-O(CH$_2$)$_3$OTBDMS-1-(CH$_2$)$_2$OH-1,2-C$_2$B$_9$H$_9$)]$_2^-$ **(11)**.

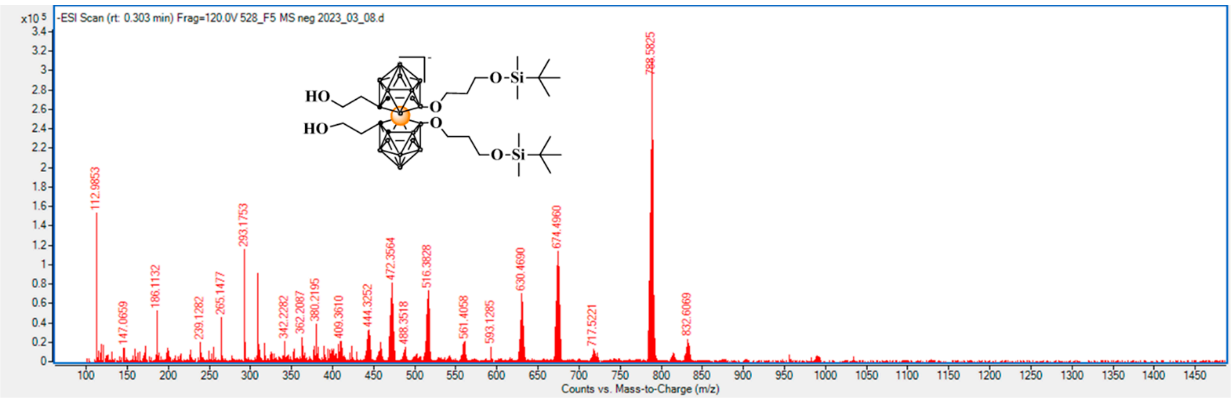

Figure S27. MS (ESI) spectrum of 3,3'-Co[(8-O(CH$_2$)$_3$OTBDMS-1-(CH$_2$)$_2$OH-1,2-$C_2B_9H_9$)]$_2^-$ **(11)**.

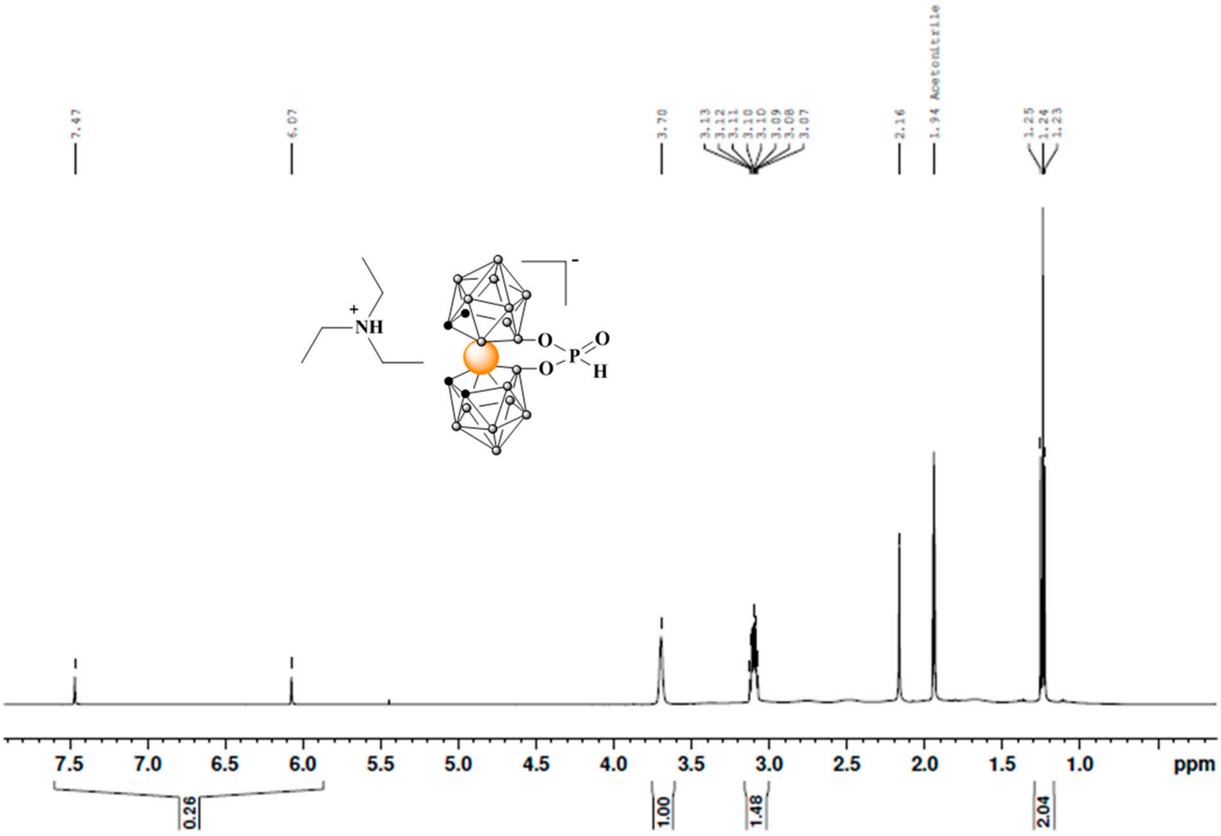

Figure S28. ^{1}H NMR spectrum of 8,8'-bridged [8,8'-O_2P(O)H-3,3'-Co(1,2-$C_2B_9H_{10}$)$_2$] $HNEt_3$ H-phosphonate **(12)**.

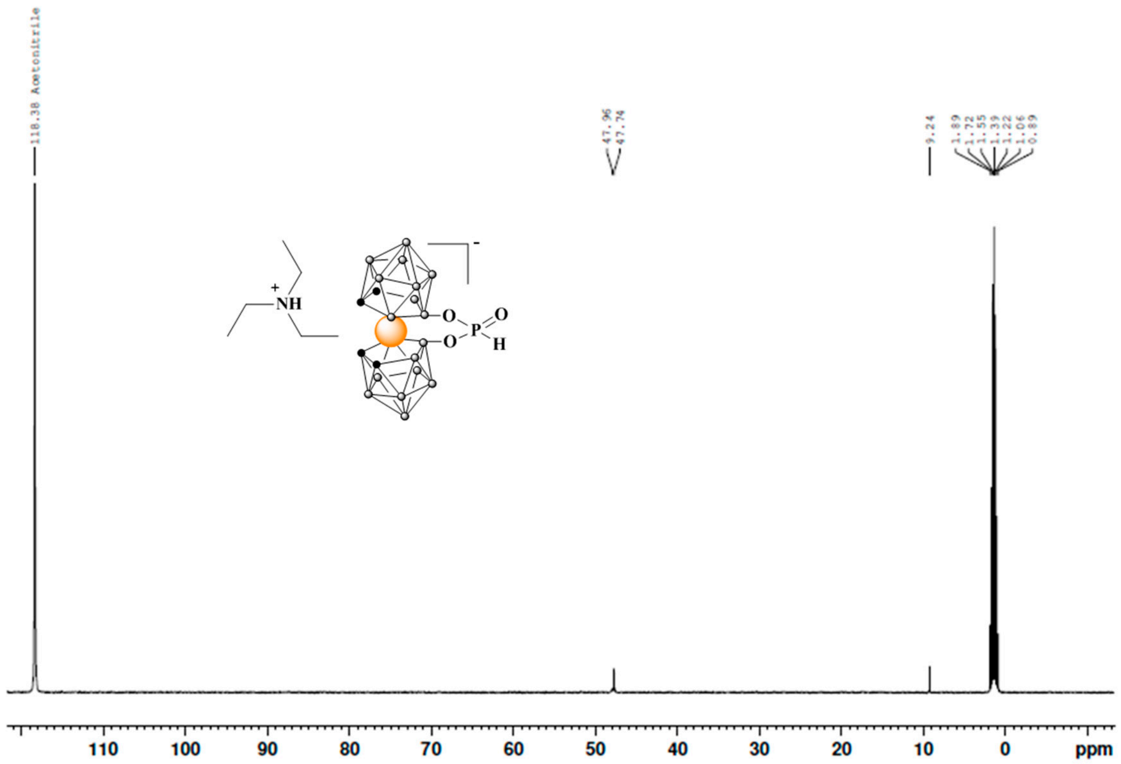

Figure S29. $^{13}C\{^{1}H\}$ NMR spectrum 8,8'-bridged [8,8'-$O_2P(O)H$-3,3'-Co(1,2-$C_2B_9H_{10}$)$_2$] $HNEt_3$ H-phosphonate **(12)**.

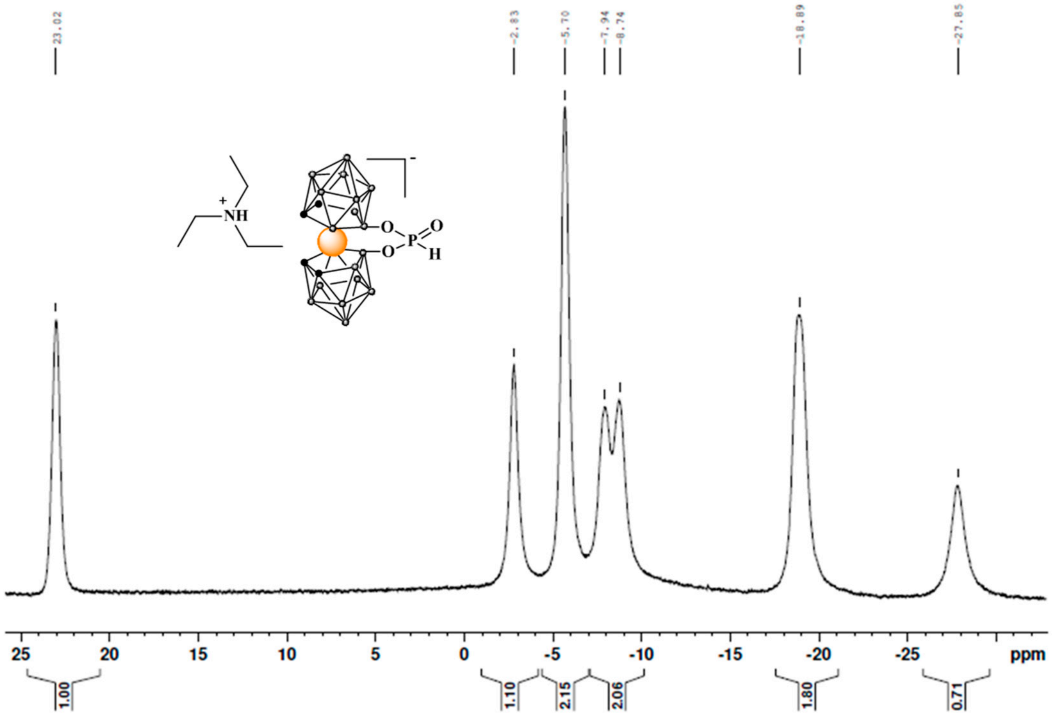

Figure S30. $^{11}B\{^{1}H\}$ NMR spectrum of 8,8'-bridged [8,8'-$O_2P(O)H$-3,3'-Co(1,2-$C_2B_9H_{10}$)$_2$] $HNEt_3$ H-phosphonate **(12)**.

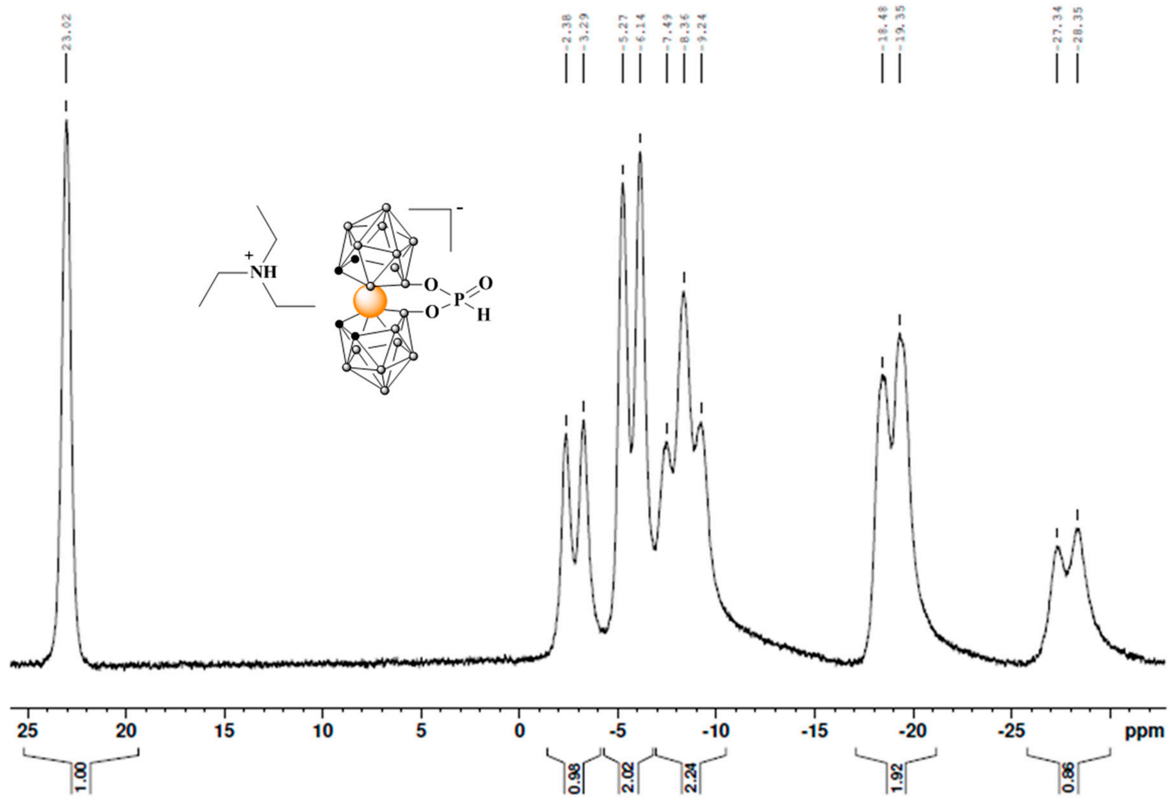

Figure S31. ^{11}B NMR spectrum of 8,8'-bridged [8,8'-$O_2P(O)H$-3,3'-Co(1,2-$C_2B_9H_{10})_2$] $HNEt_3$ H-phosphonate **(12)**.

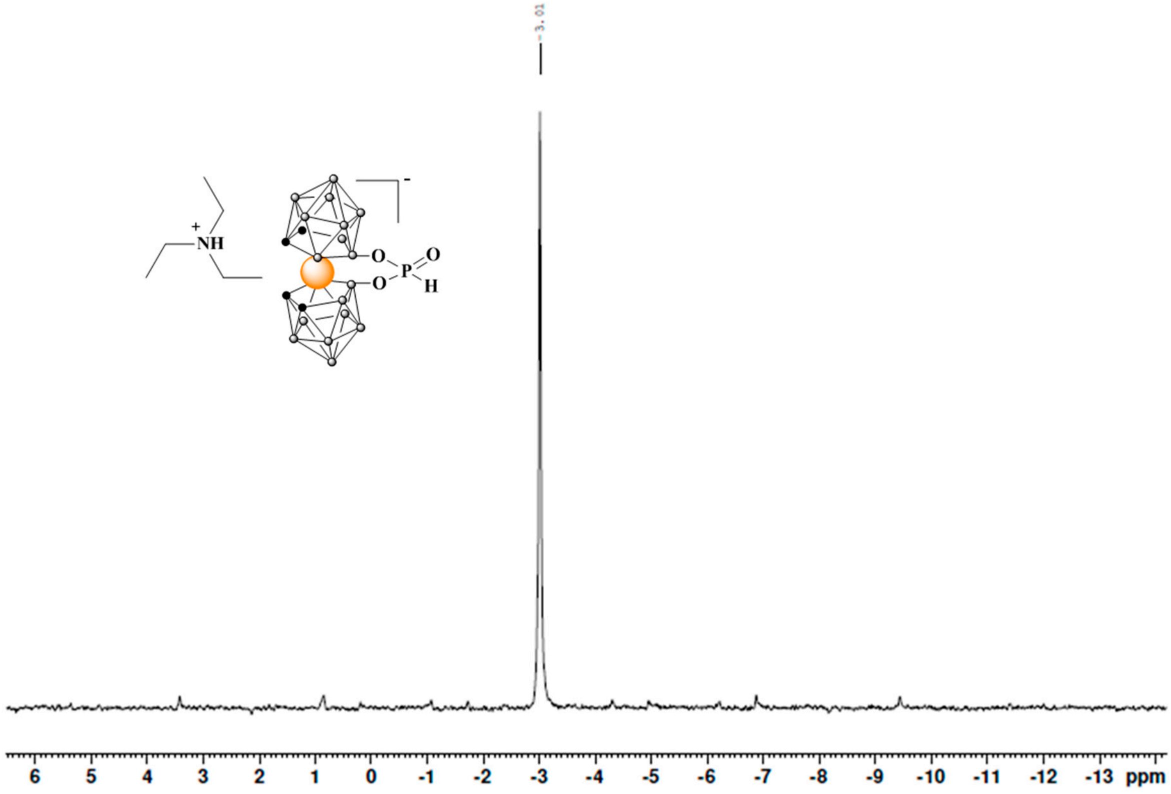

Figure S32. $^{31}P\{^1H\}$ NMR spectrum of 8,8'-bridged [8,8'-$O_2P(O)H$-3,3'-Co(1,2-$C_2B_9H_{10})_2$] $HNEt_3$ H-phosphonate **(12)**.

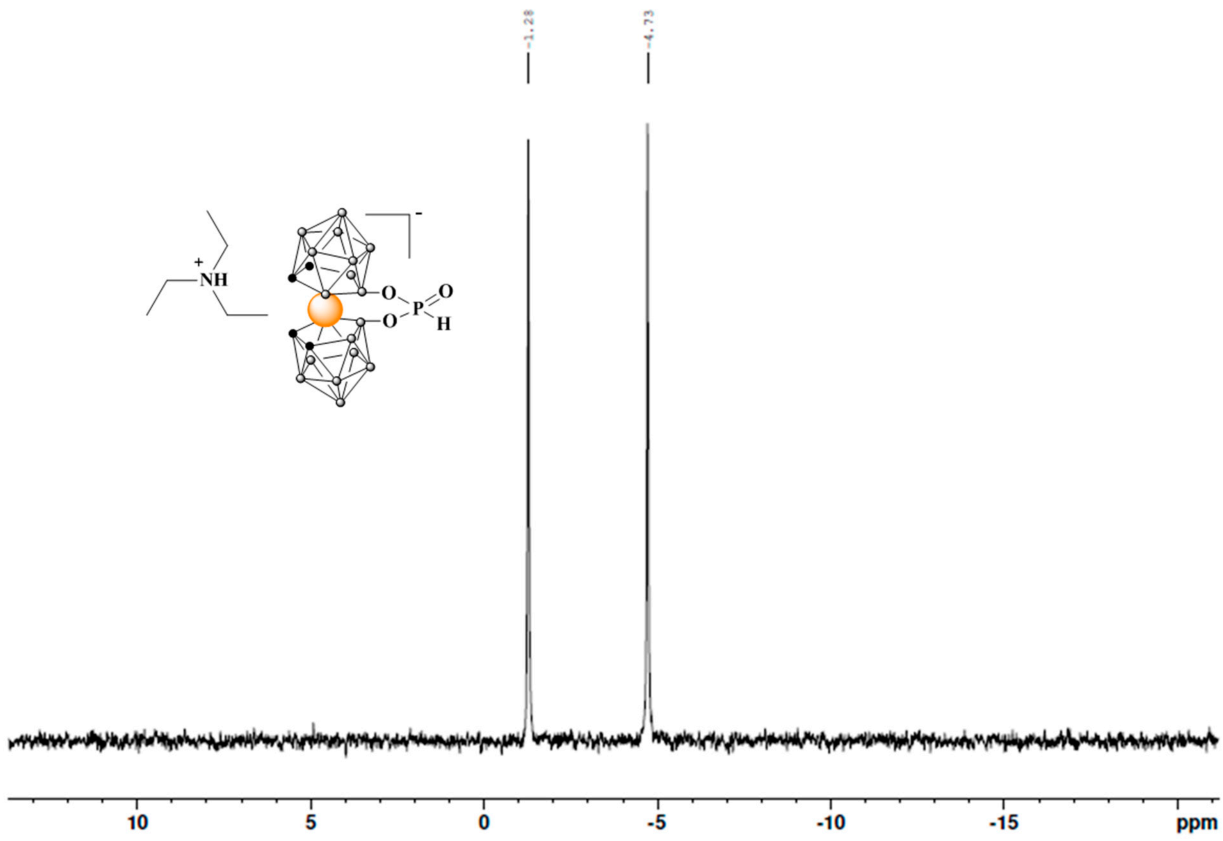

Figure S33. ^{31}P NMR spectrum of 8,8'-bridged [8,8'-$O_2P(O)H$-3,3'-Co(1,2-$C_2B_9H_{10}$)$_2$] $HNEt_3$ H-phosphonate **(12).**

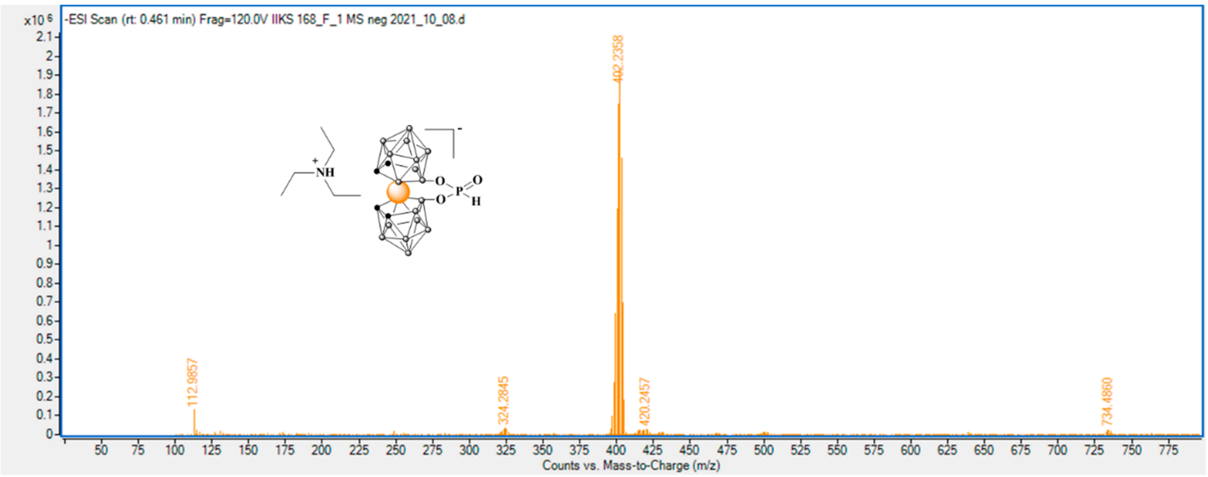

Figure S34. MS (ESI) spectrum of 8,8'-bridged [8,8'-$O_2P(O)H$-3,3'-Co(1,2-$C_2B_9H_{10}$)$_2$] $HNEt_3$ H-phosphonate **(12).**

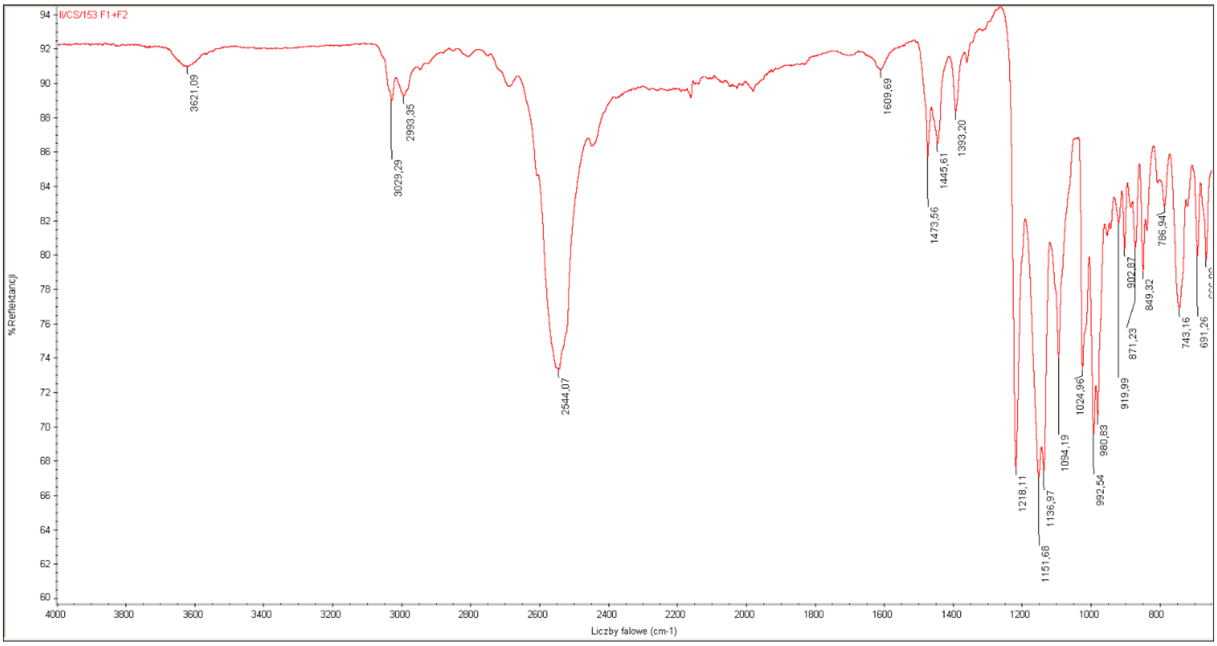

Figure S35. FT-IR spectrum of 8,8'-bridged [8,8'-$O_2P(O)H$-3,3'-Co(1,2-$C_2B_9H_{10}$)$_2$] $HNEt_3$ H-phosphonate **(12)**.

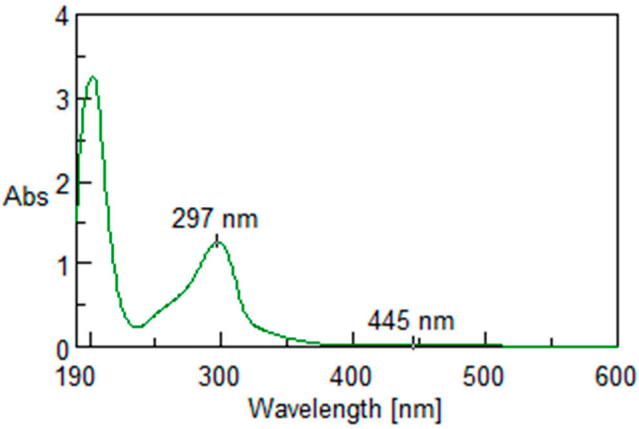

Figure S36. UV-VIS spectrum of 8,8'-bridged [8,8'-$O_2P(O)H$-3,3'-Co(1,2-$C_2B_9H_{10}$)$_2$] $HNEt_3$ H-phosphonate **(12)**.

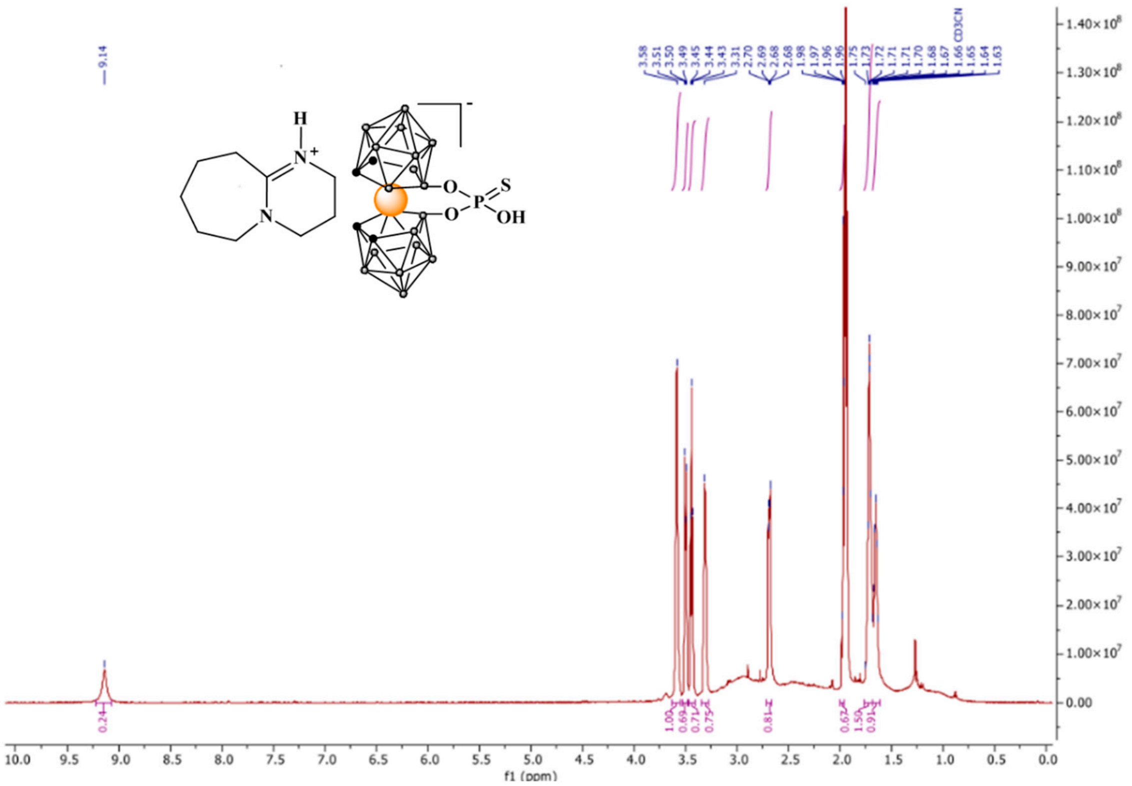

Figure S37. ^{1}H NMR spectrum of 8,8'-bridged [8,8'-O_2P(O)SH-3,3'-Co(1,2-$C_2B_9H_{10}$)$_2$] HDBU phosphorothioate **(13).**

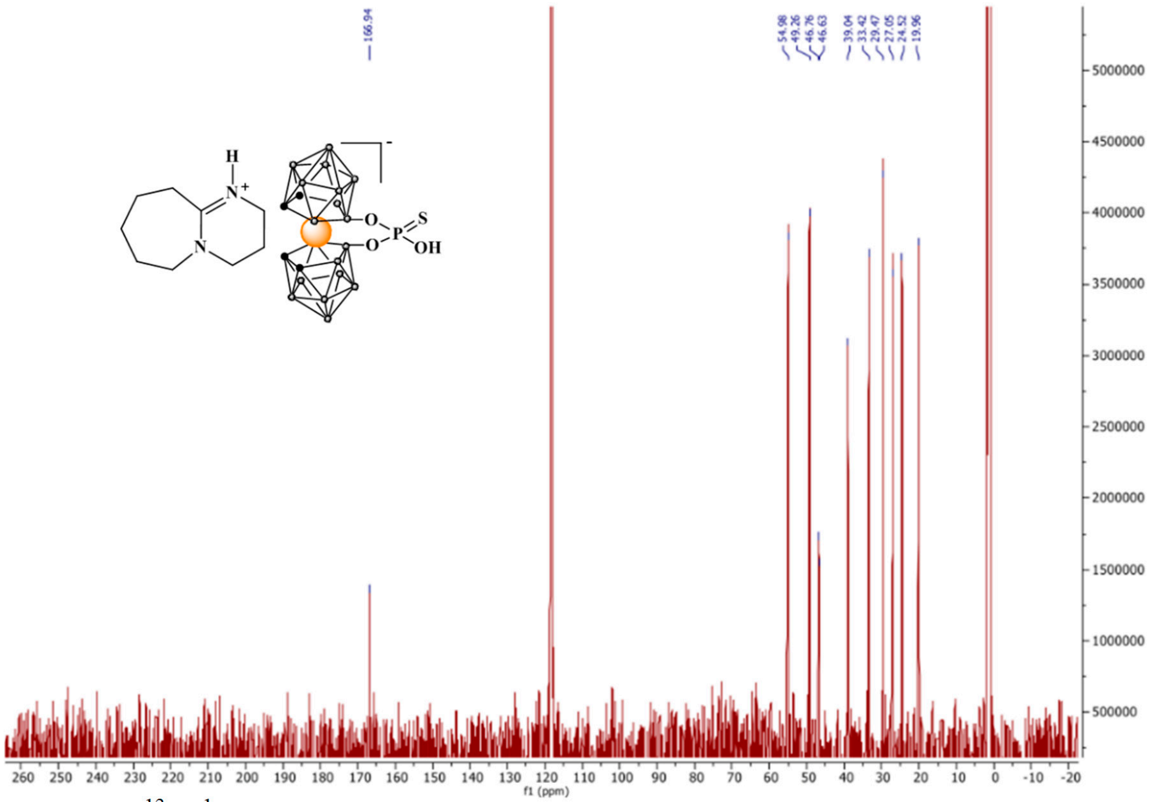

Figure S38. ^{13}C{^{1}H} NMR spectrum of 8,8'-bridged [8,8'-O_2P(O)SH-3,3'-Co(1,2-$C_2B_9H_{10}$)$_2$] HDBU phosphorothioate **(13).**

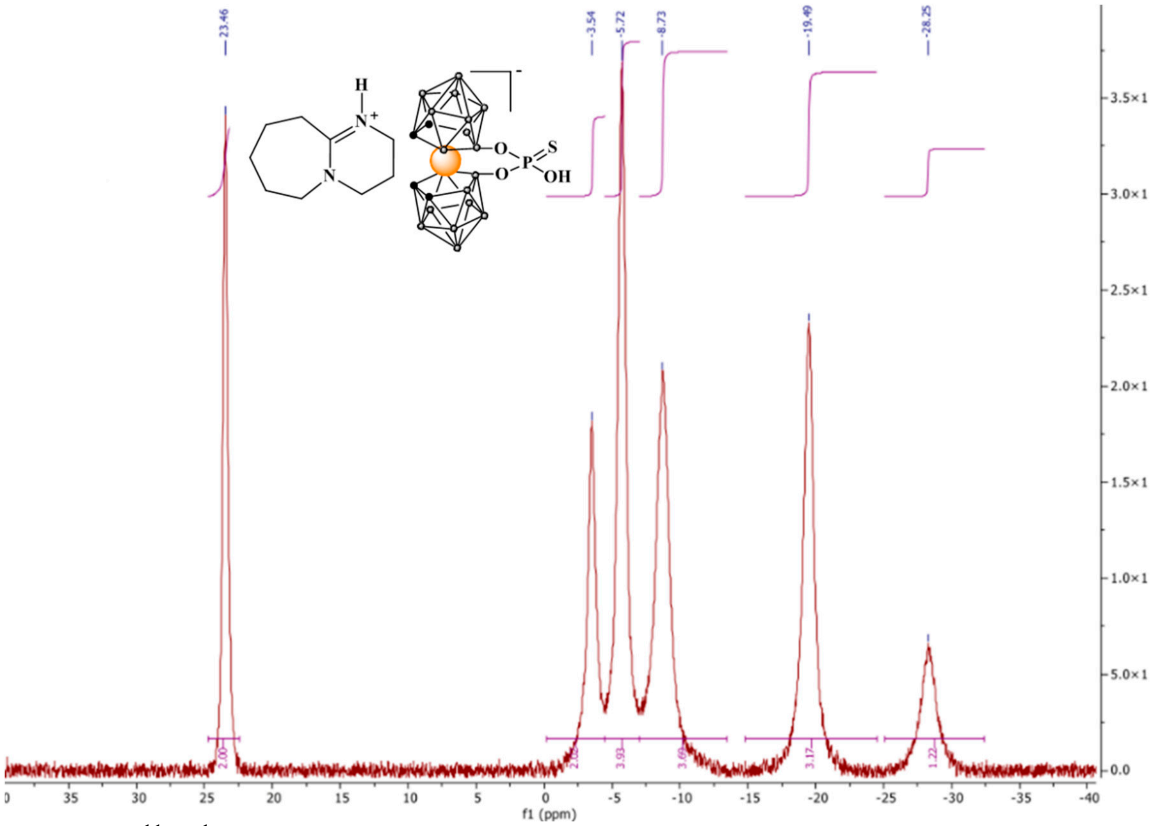

Figure S39. $^{11}B\{^{1}H\}$ NMR spectrum of 8,8'-bridged [8,8'-$O_2P(O)SH$-3,3'-Co(1,2-$C_2B_9H_{10}$)$_2$] HDBU phosphorothioate **(13).**

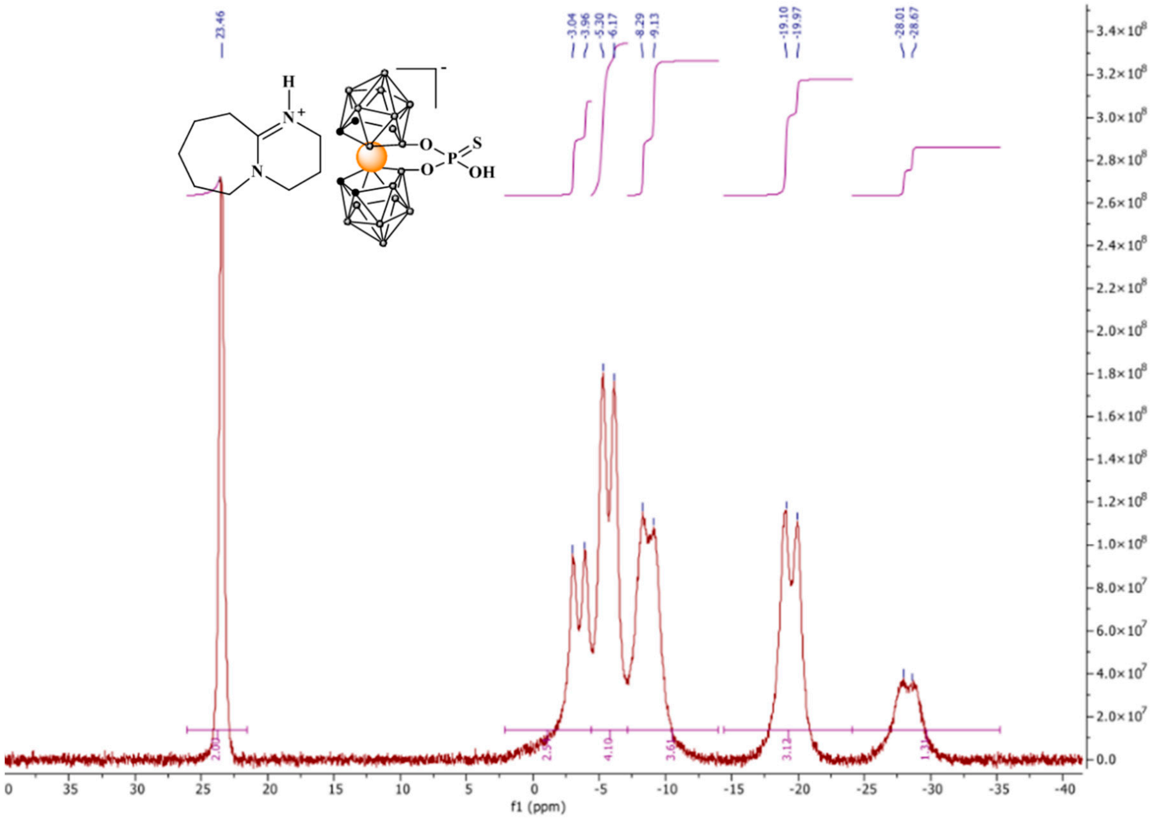

Figure S40. ^{11}B NMR spectrum of 8,8'-bridged [8,8'-$O_2P(O)SH$-3,3'-Co(1,2-$C_2B_9H_{10}$)$_2$] HDBU phosphorothioate **(13).**

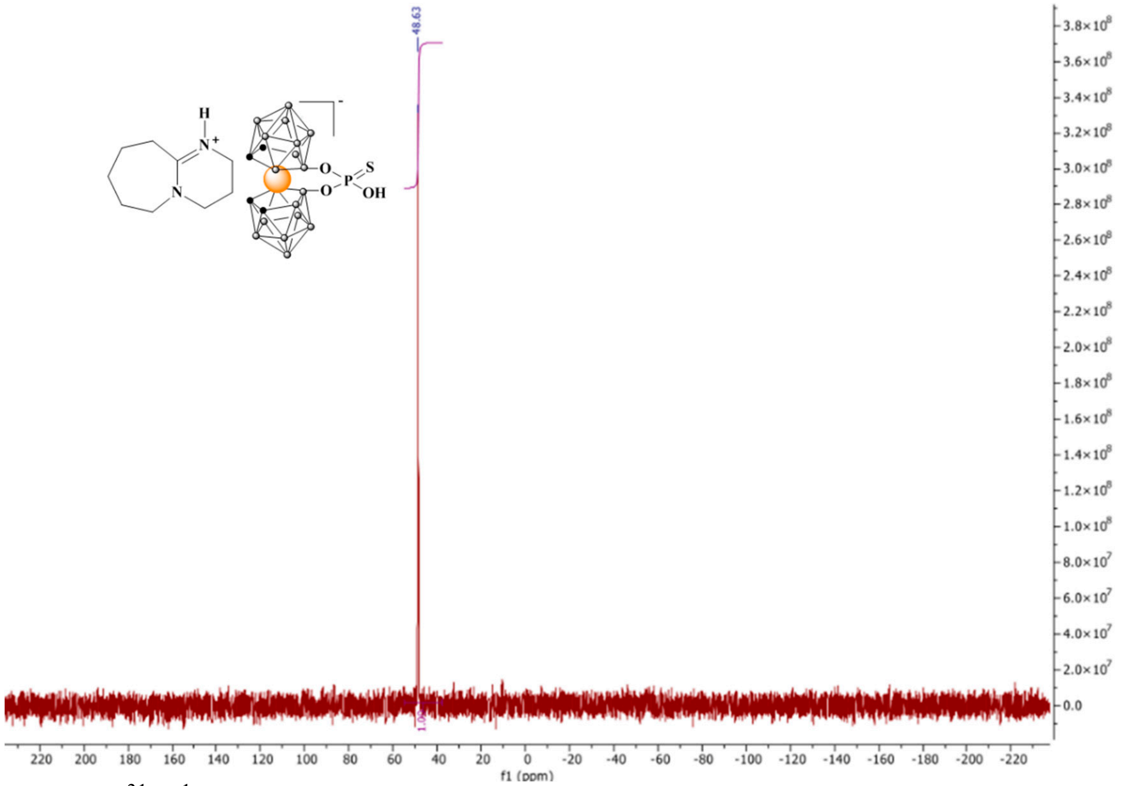

Figure S41. $^{31}P\{^{1}H\}$ NMR spectrum of 8,8’-bridged [8,8'-$O_2P(O)SH$-3,3'-Co(1,2-$C_2B_9H_{10}$)$_2$] HDBU phosphorothioate **(13).**

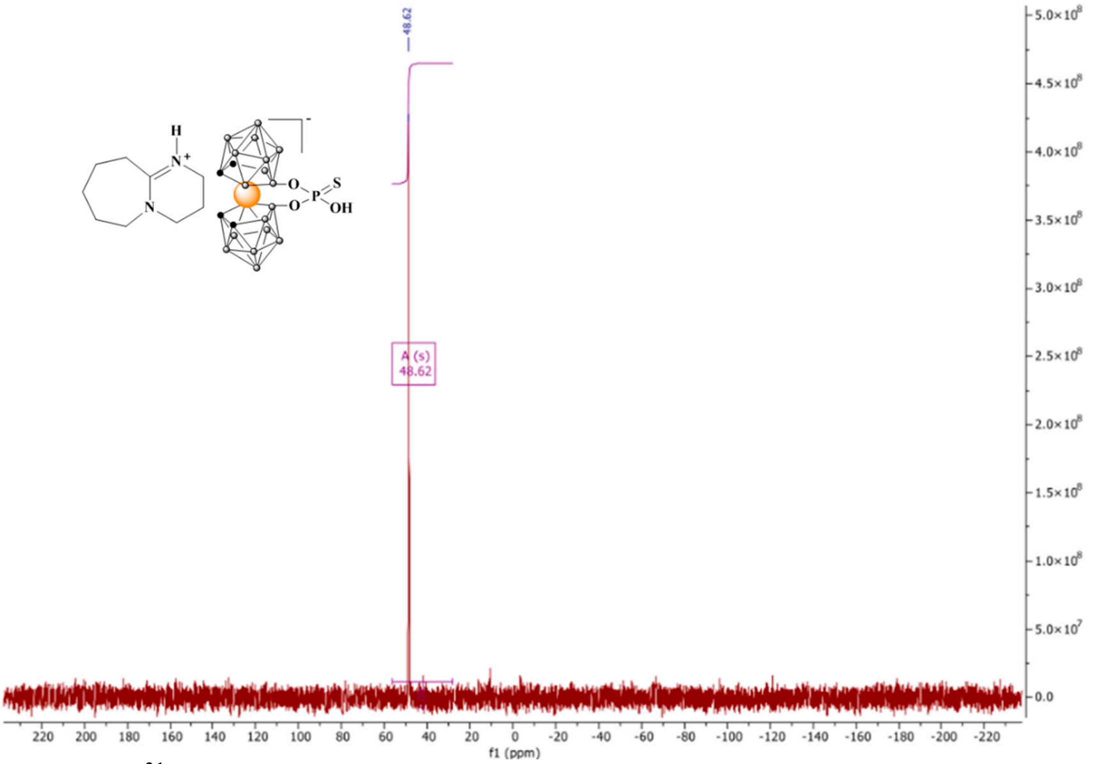

Figure S42. ^{31}P NMR spectrum of 8,8’-bridged [8,8'-$O_2P(O)SH$-3,3'-Co(1,2-$C_2B_9H_{10}$)$_2$] HDBU phosphorothioate **(13).**

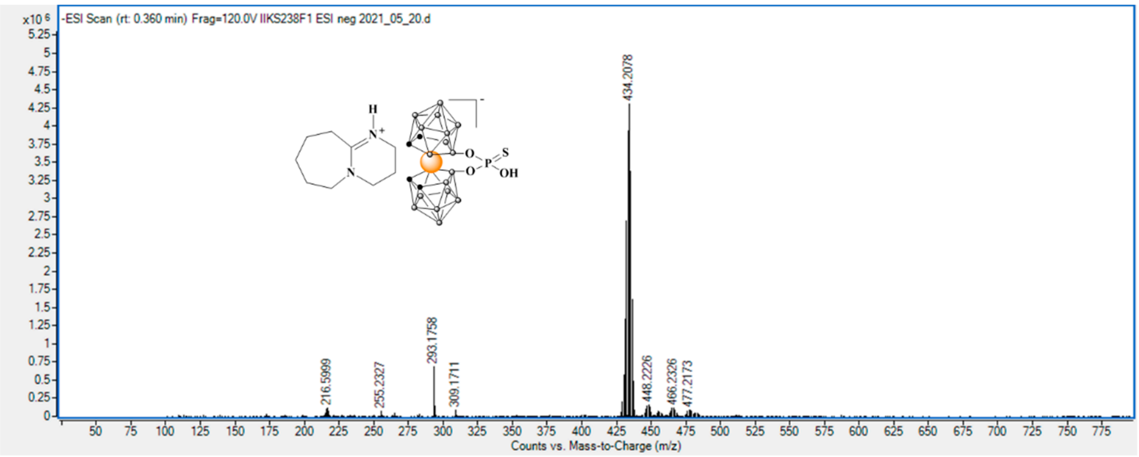

Figure S43. MS (ESI) spectrum of 8,8'-bridged [8,8'-$O_2P(O)SH$-3,3'-Co(1,2-$C_2B_9H_{10}$)$_2$] HDBU phosphorothioate **(13).**

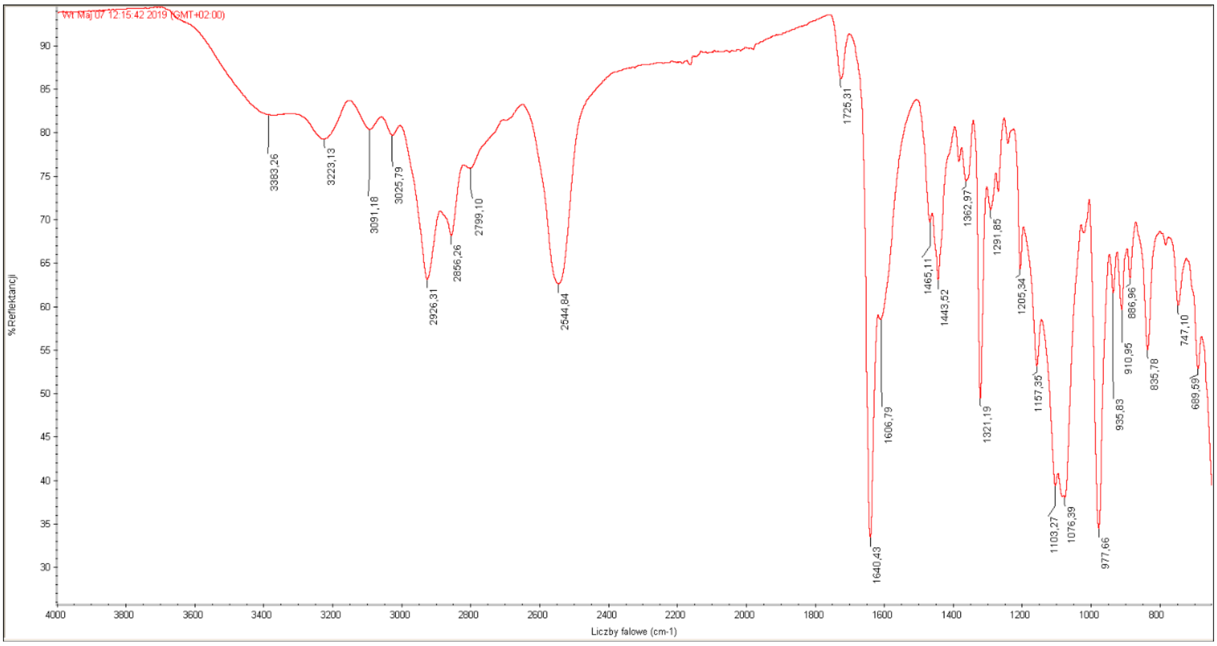

Figure S44. FT-IR spectrum of 8,8'-bridged [8,8'-$O_2P(O)SH$-3,3'-Co(1,2-$C_2B_9H_{10}$)$_2$] HDBU phosphorothioate **(13).**

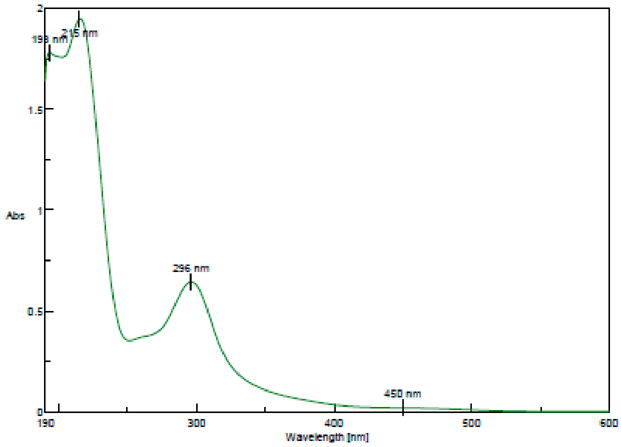

Figure S45. UV-VIS spectrum of spectrum of 8,8'-bridged [8,8'-$O_2P(O)SH$-3,3'-Co(1,2-$C_2B_9H_{10}$)$_2$] HDBU phosphorothioate **(13).**

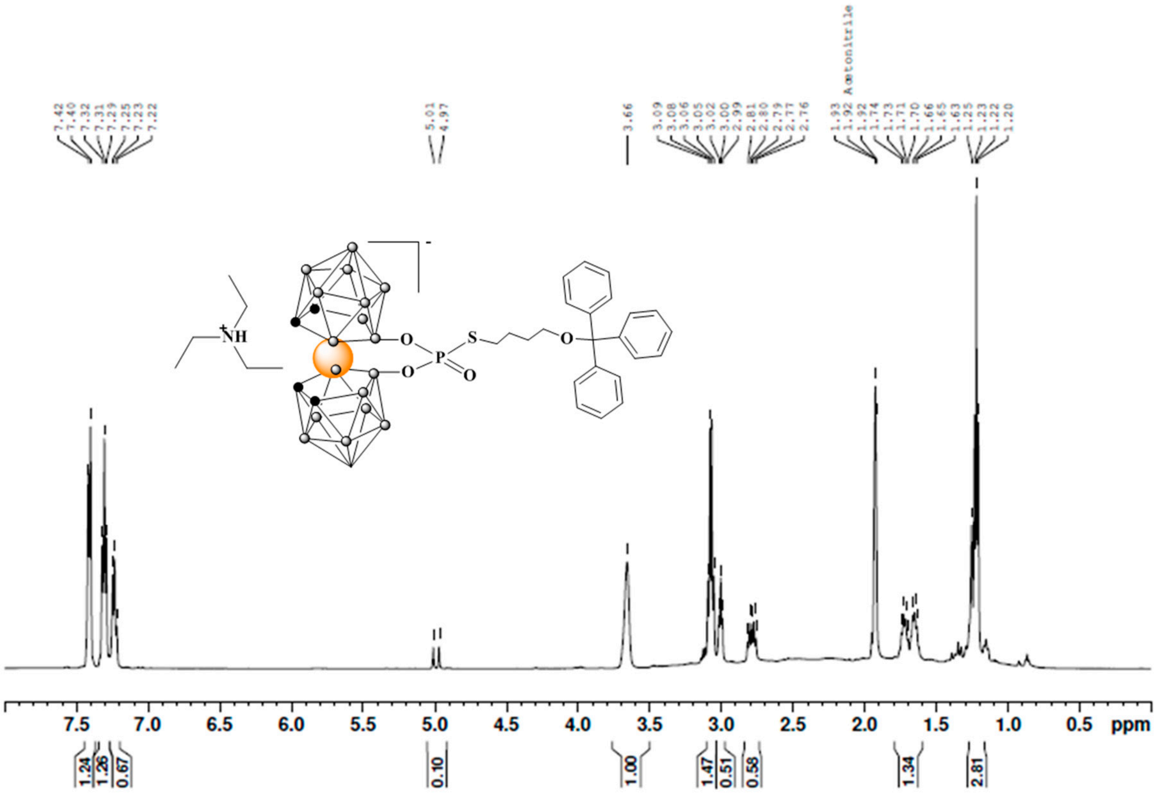

Figure S46. ^{1}H NMR spectrum of [8,8'-$O_2P(O)S(CH_2)_4OCPh_3$-3,3'-Co(1,2-$C_2B_9H_{10})_2$] $HNEt_3$ **(15)**.

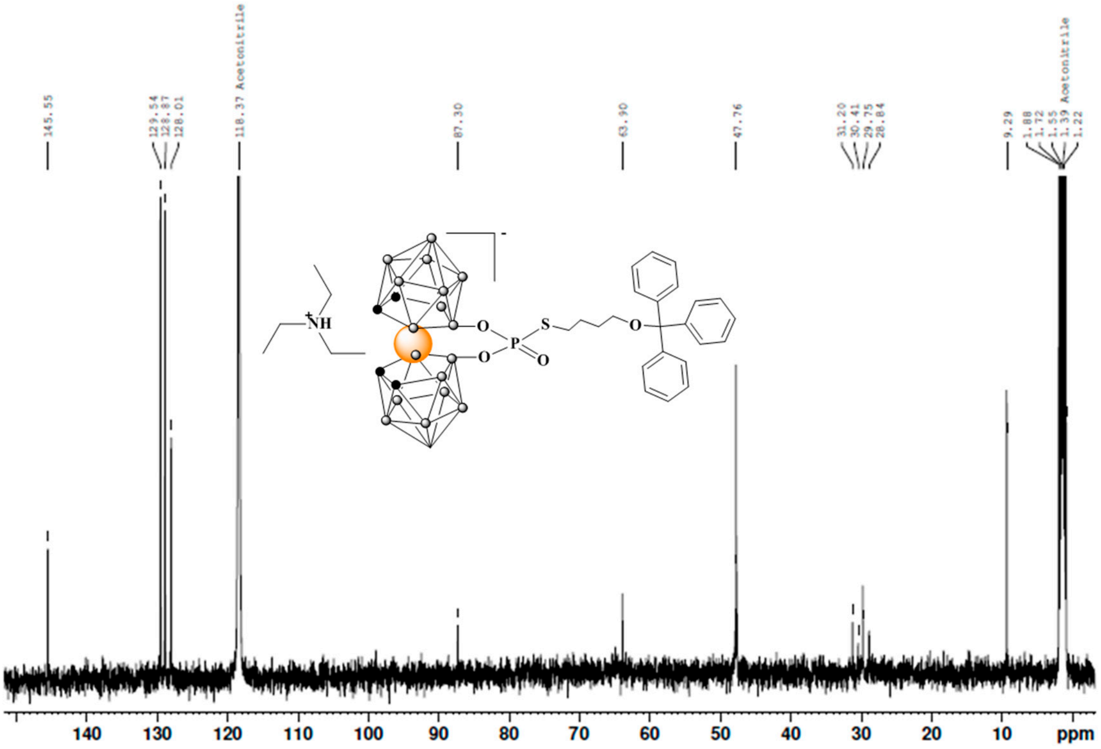

Figure S47. ^{13}C{^{1}H} NMR spectrum of [8,8'-$O_2P(O)S(CH_2)_4OCPh_3$-3,3'-Co(1,2-$C_2B_9H_{10})_2$] $HNEt_3$ **(15)**.

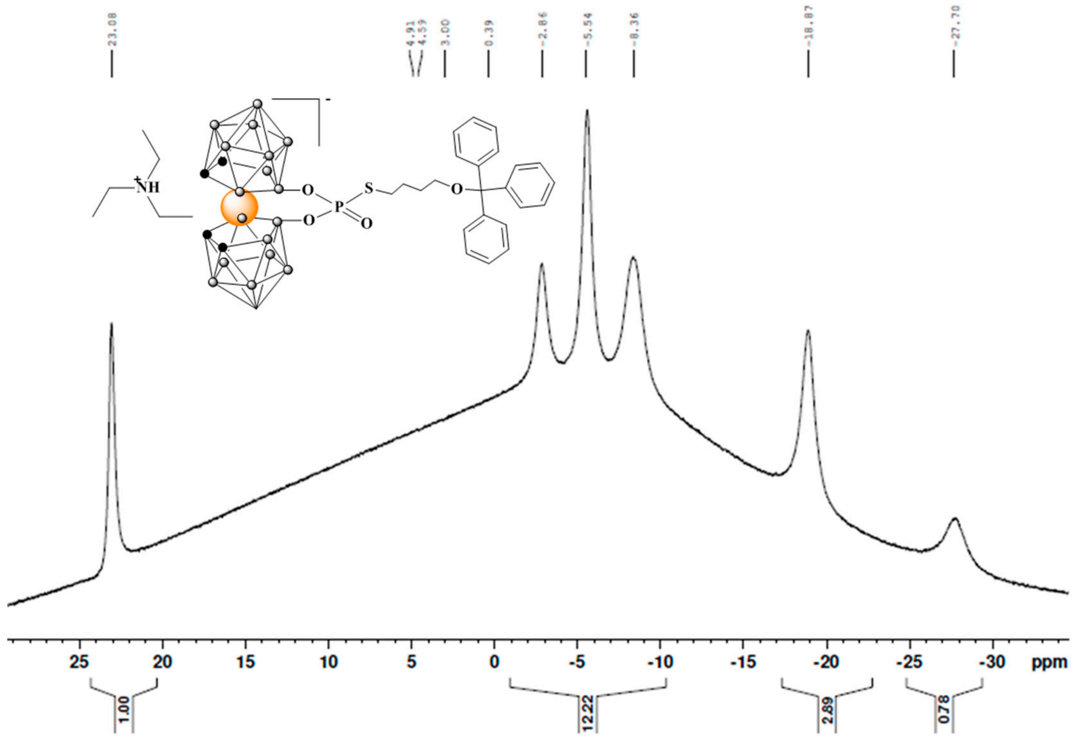

Figure S48. $^{11}B\{^{1}H\}$ NMR spectrum of [8,8'-$O_2P(O)S(CH_2)_4OCPh_3$-3,3'-Co(1,2-$C_2B_9H_{10})_2$] $HNEt_3$ **(15)**.

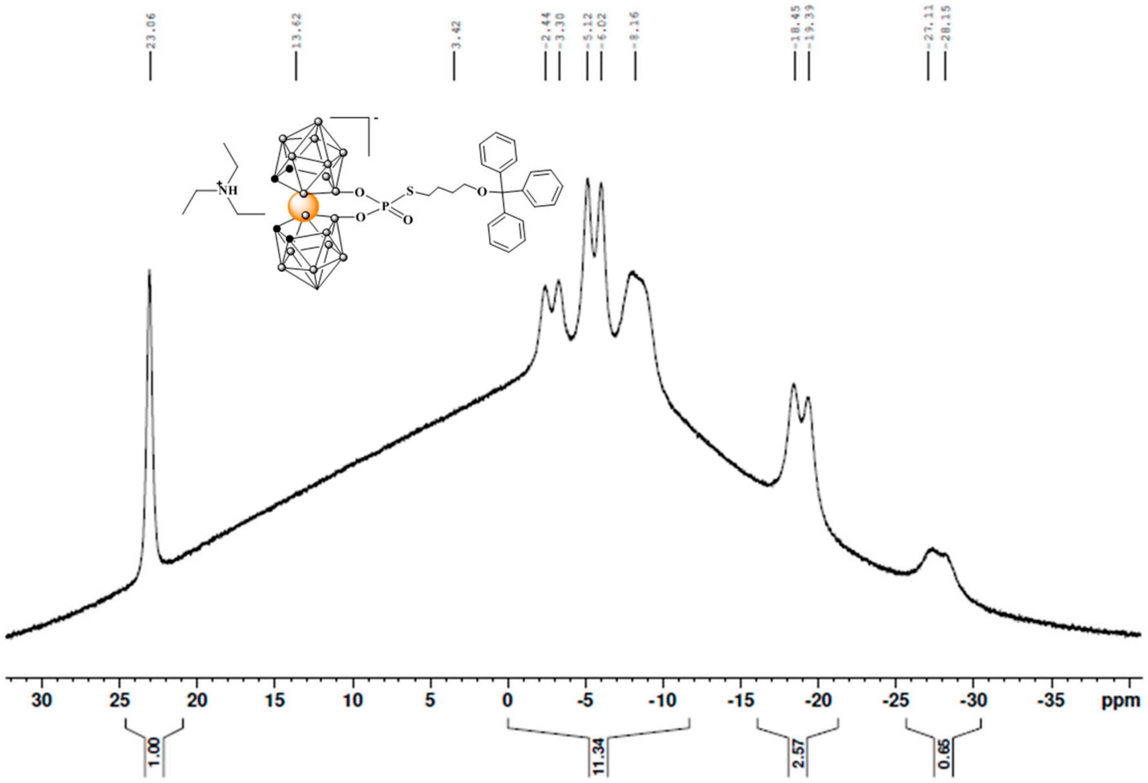

Figure S49. ^{11}B NMR spectrum of [8,8'-$O_2P(O)S(CH_2)_4OCPh_3$-3,3'-Co(1,2-$C_2B_9H_{10})_2$] $HNEt_3$ **(15)**.

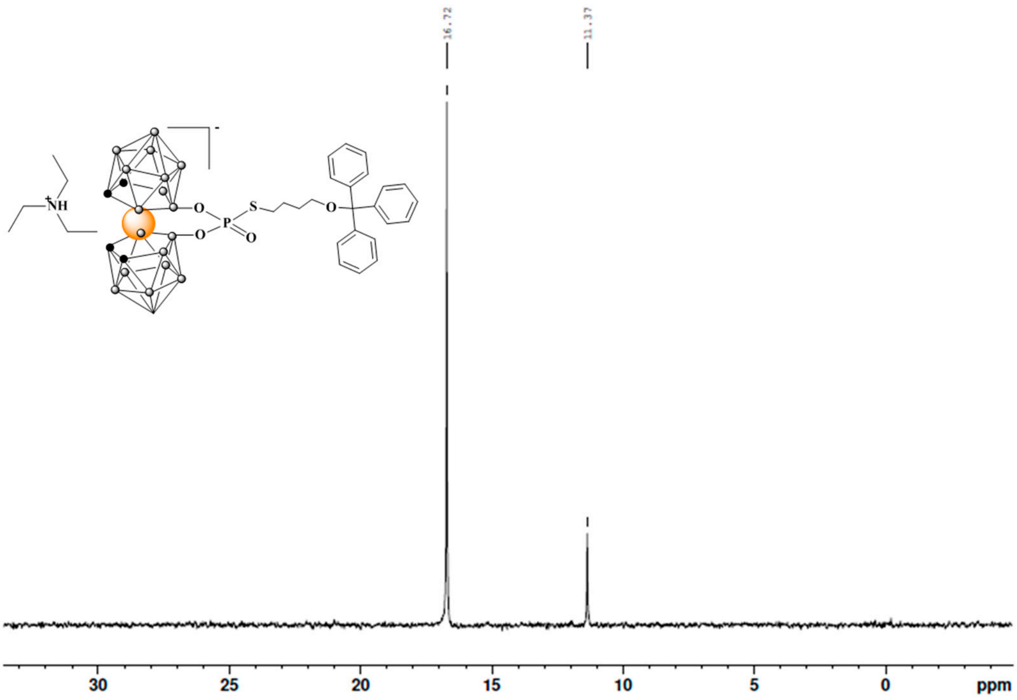

Figure S50. $^{31}P\{^{1}H\}$ NMR spectrum of $[8,8'-O_2P(O)S(CH_2)_4OCPh_3-3,3'-Co(1,2-C_2B_9H_{10})_2]$ $HNEt_3$ **(15)**.

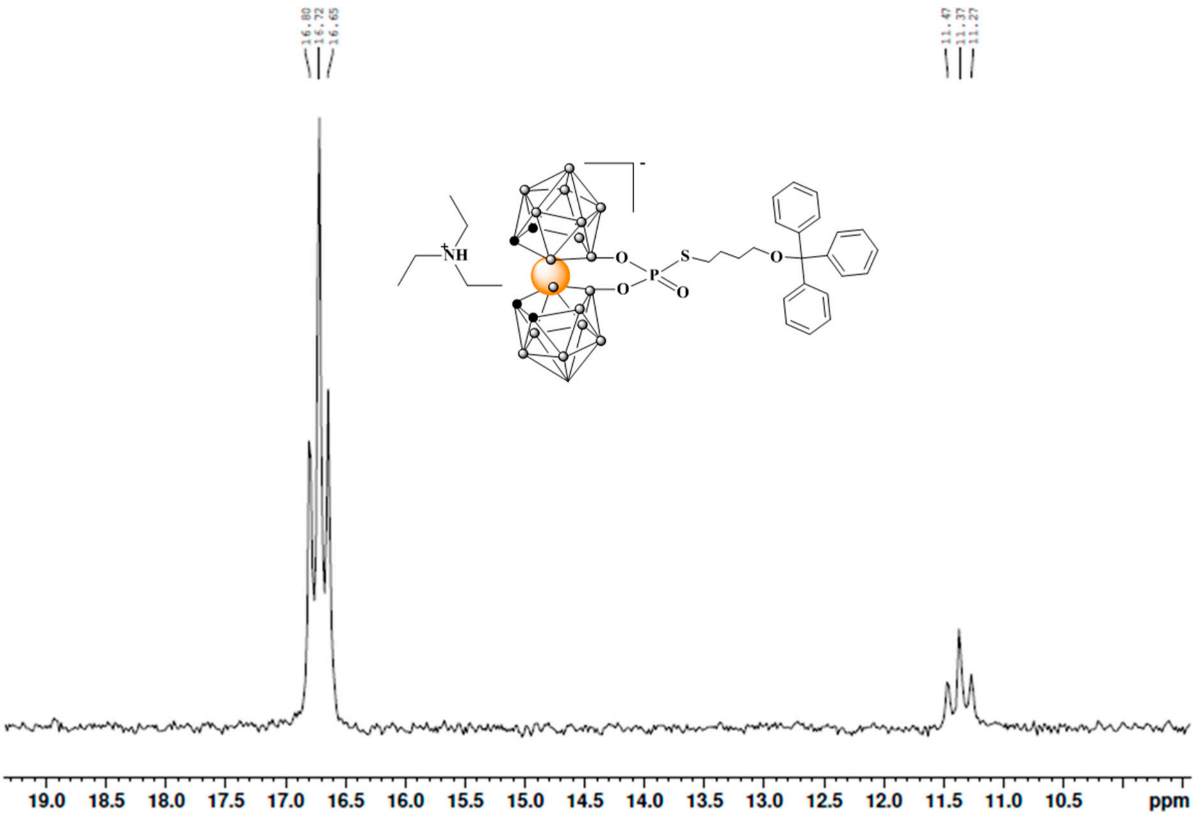

Figure S51. ^{31}P NMR spectrum of $[8,8'-O_2P(O)S(CH_2)_4OCPh_3-3,3'-Co(1,2-C_2B_9H_{10})_2]$ $HNEt_3$ **(15)**.

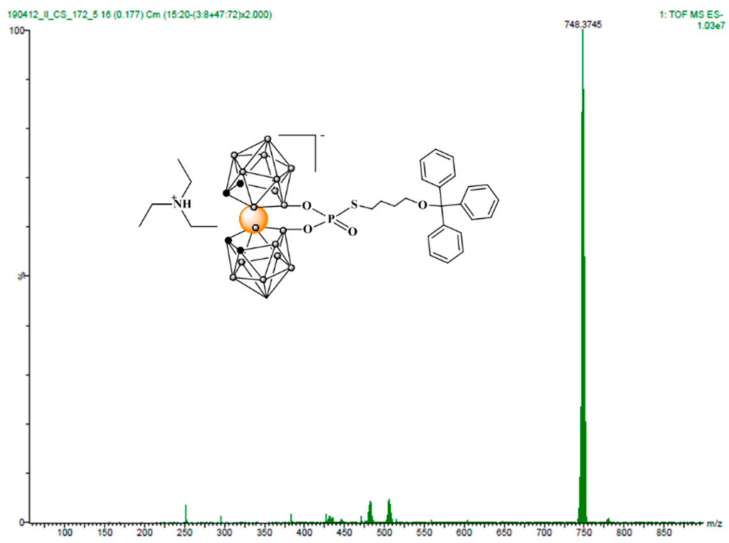

Figure S52. MS (ESI) spectrum of [8,8'-$O_2P(O)S(CH_2)_4OCPh_3$-3,3'-Co(1,2-$C_2B_9H_{10})_2$] $HNEt_3$ (**15**).

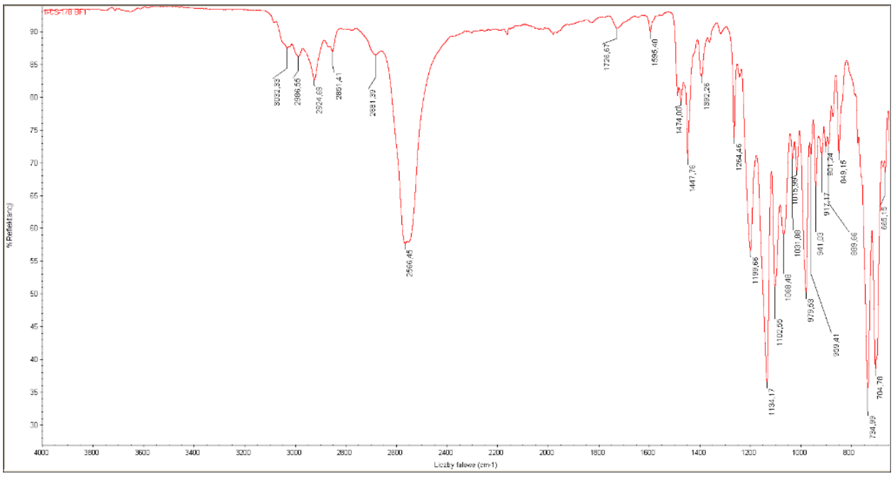

Figure S53. FT-IR spectrum of [8,8'-$O_2P(O)S(CH_2)_4OCPh_3$-3,3'-Co(1,2-$C_2B_9H_{10})_2$] $HNEt_3$ (**15**).

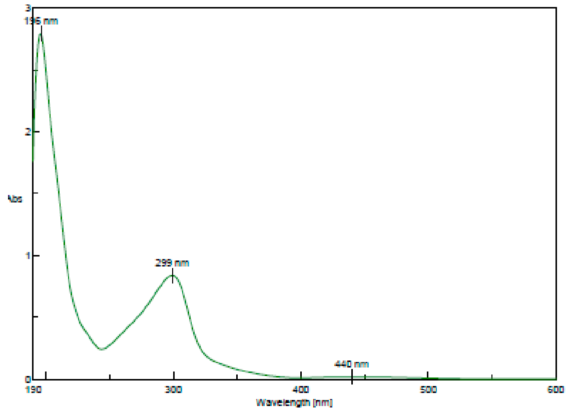

Figure S54. UV-VIS spectrum of [8,8'-$O_2P(O)S(CH_2)_4OCPh_3$-3,3'-Co(1,2-$C_2B_9H_{10})_2$] $HNEt_3$ (**15**).

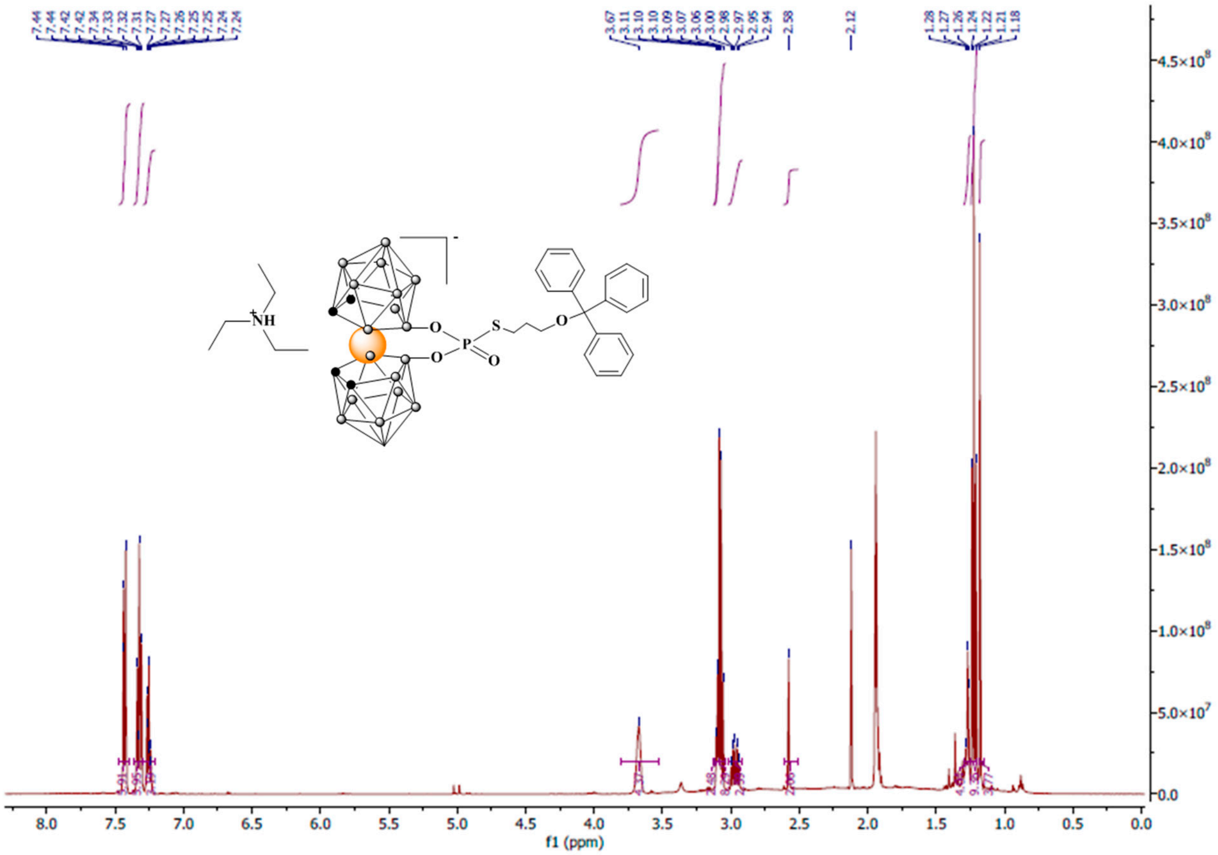

Figure S55. ^{1}H NMR spectrum of [8,8'-$O_2P(O)S(CH_2)_3OCPh_3$-3,3'-Co(1,2-$C_2B_9H_{10})_2$] $HNEt_3$ **(16)**.

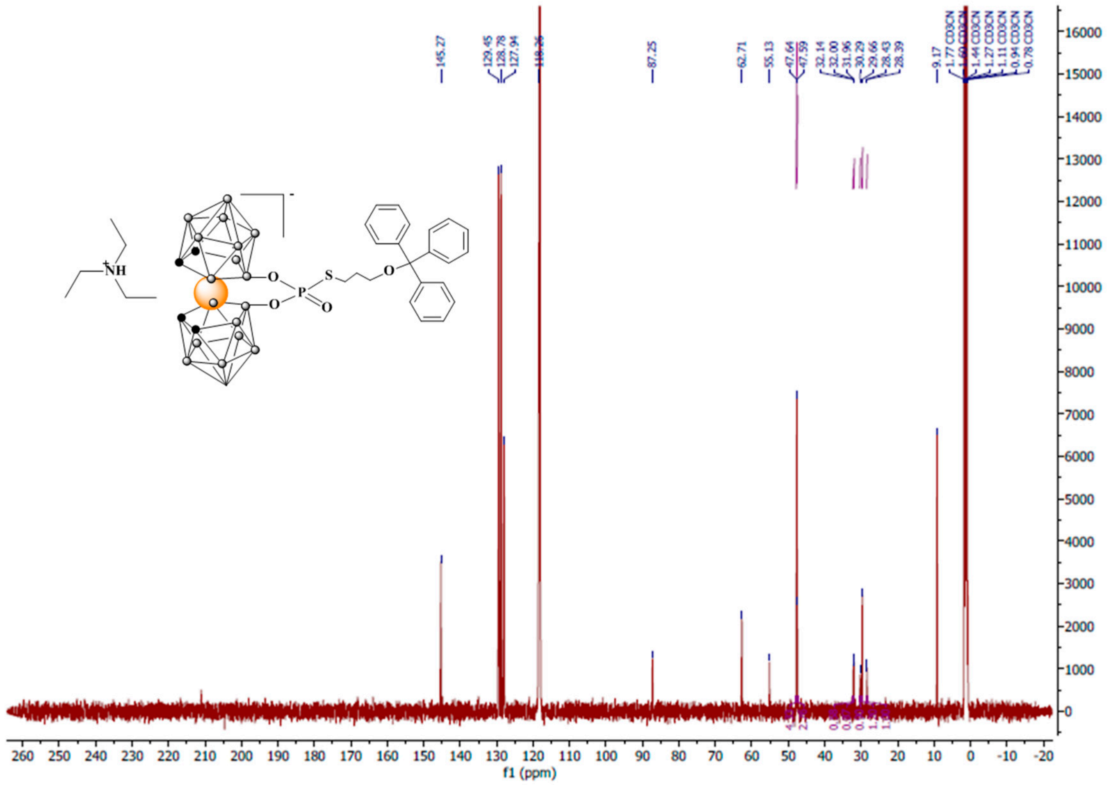

Figure S56. $^{13}C\{^{1}H\}$ NMR spectrum of [8,8'-$O_2P(O)S(CH_2)_3OCPh_3$-3,3'-Co(1,2-$C_2B_9H_{10})_2$] $HNEt_3$ **(16)**.

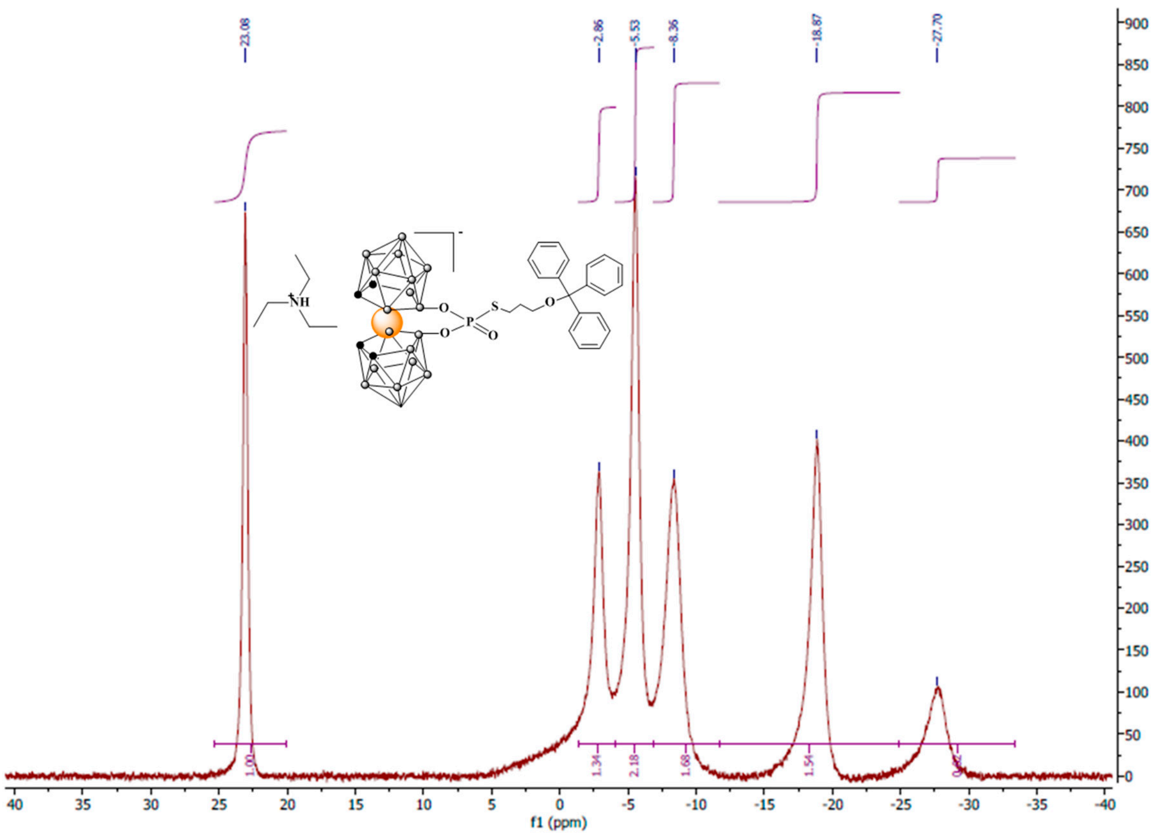

Figure S57. $^{11}B\{^{1}H\}$ NMR spectrum of [8,8'-$O_2P(O)S(CH_2)_3OCPh_3$-3,3'-Co(1,2-$C_2B_9H_{10})_2$] $HNEt_3$ **(16)**.

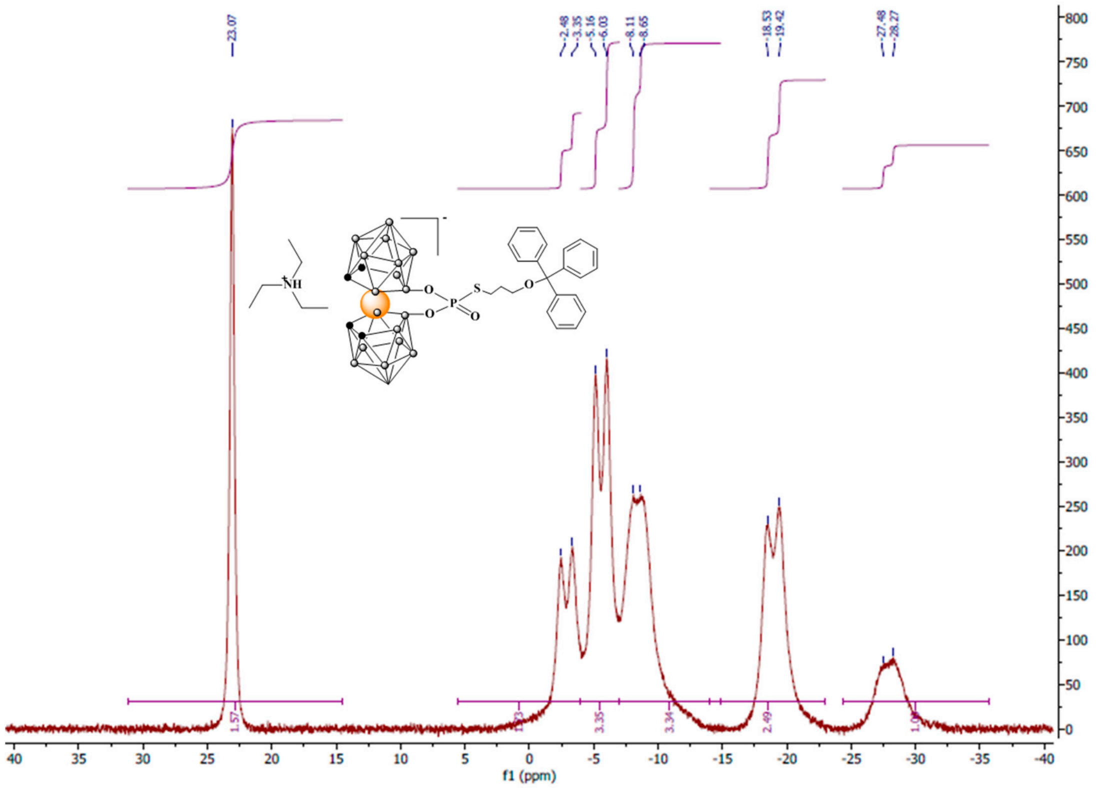

Figure S58. ^{11}B NMR spectrum of [8,8'-$O_2P(O)S(CH_2)_3OCPh_3$-3,3'-Co(1,2-$C_2B_9H_{10})_2$] $HNEt_3$ **(16)**.

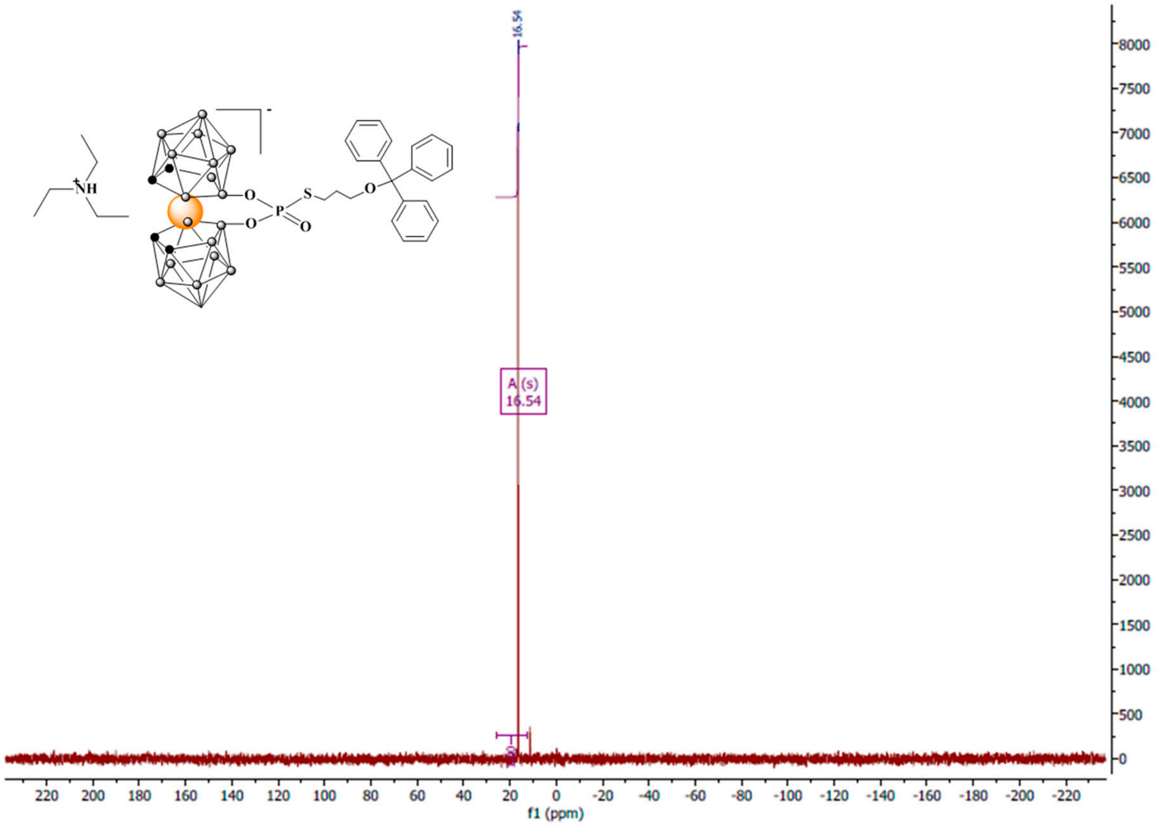

Figure S59. $^{31}P\{^{1}H\}$ NMR spectrum of [8,8'-$O_2P(O)S(CH_2)_3OCPh_3$-3,3'-Co(1,2-$C_2B_9H_{10})_2$] $HNEt_3$ **(16)**.

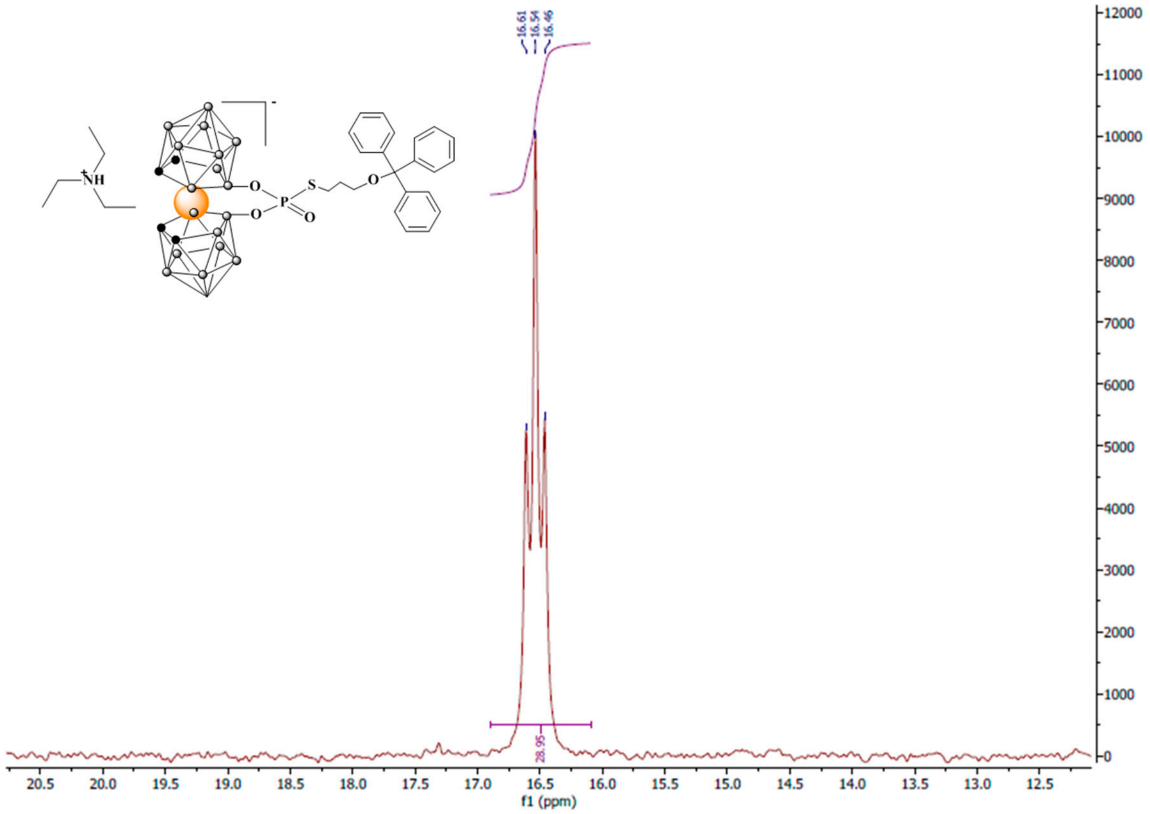

Figure S60. ^{31}P NMR spectrum of [8,8'-$O_2P(O)S(CH_2)_3OCPh_3$-3,3'-Co(1,2-$C_2B_9H_{10})_2$] $HNEt_3$ **(16)**.

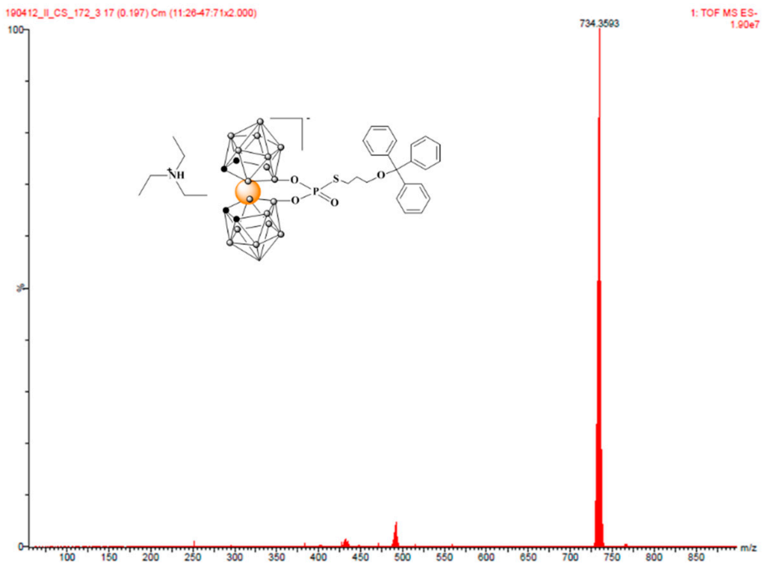

Figure S61. MS (ESI) spectrum of [8,8'-$O_2P(O)S(CH_2)_3OCPh_3$-3,3'-Co(1,2-$C_2B_9H_{10}$)$_2$] $HNEt_3$ (**16**).

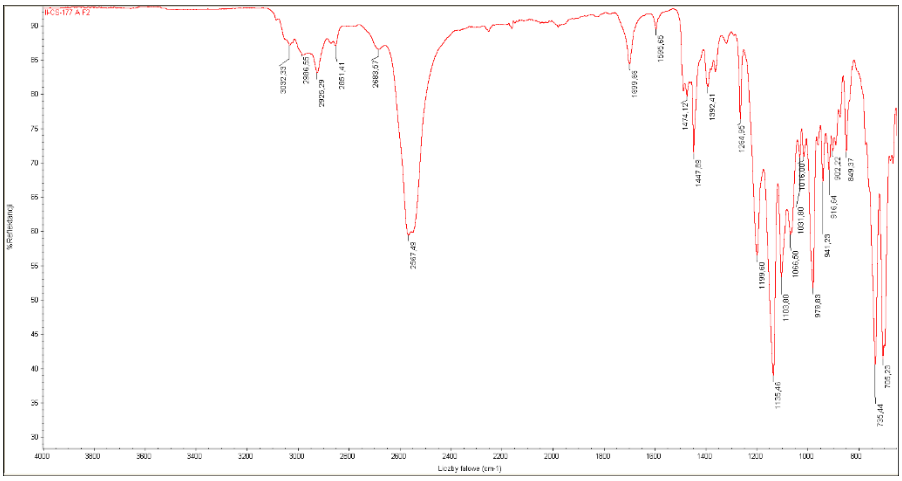

Figure S62. FT-IR spectrum of [8,8'-$O_2P(O)S(CH_2)_3OCPh_3$-3,3'-Co(1,2-$C_2B_9H_{10}$)$_2$] $HNEt_3$ (**16**).

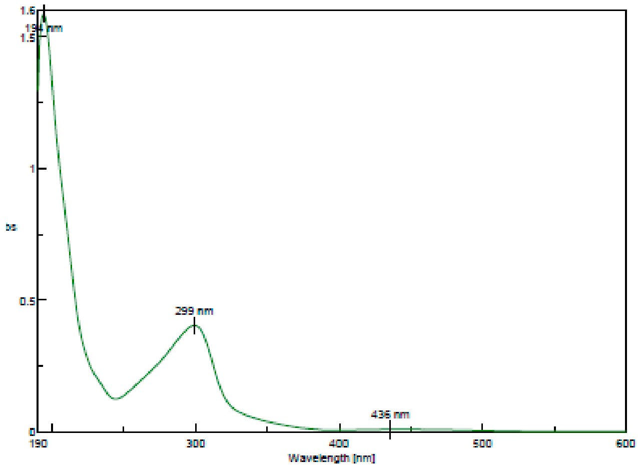

Figure S63. UV-VIS spectrum of [8,8'-$O_2P(O)S(CH_2)_3OCPh_3$-3,3'-Co(1,2-$C_2B_9H_{10}$)$_2$] $HNEt_3$ (**16**).

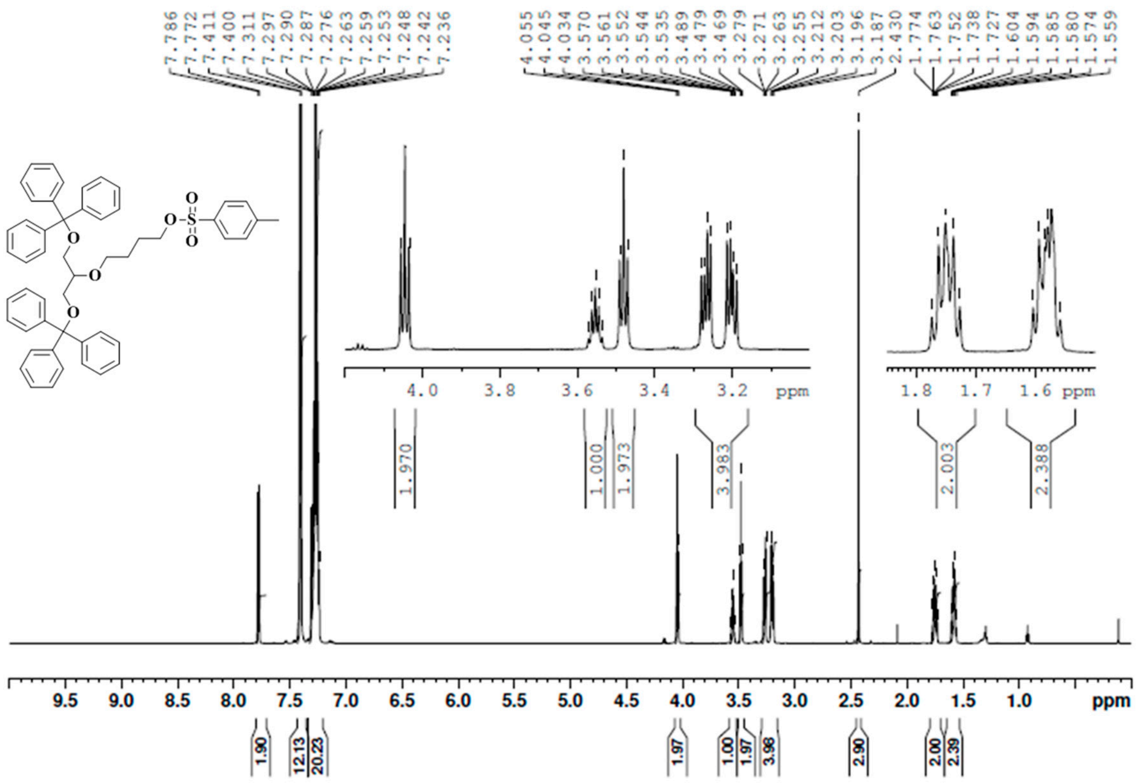

Figure S64. ^{1}H NMR spectrum of 4-(1,3-bis(trityloxy)propan-2-yloxy)butyl4-methylbenzenesulfonate **(20)**.

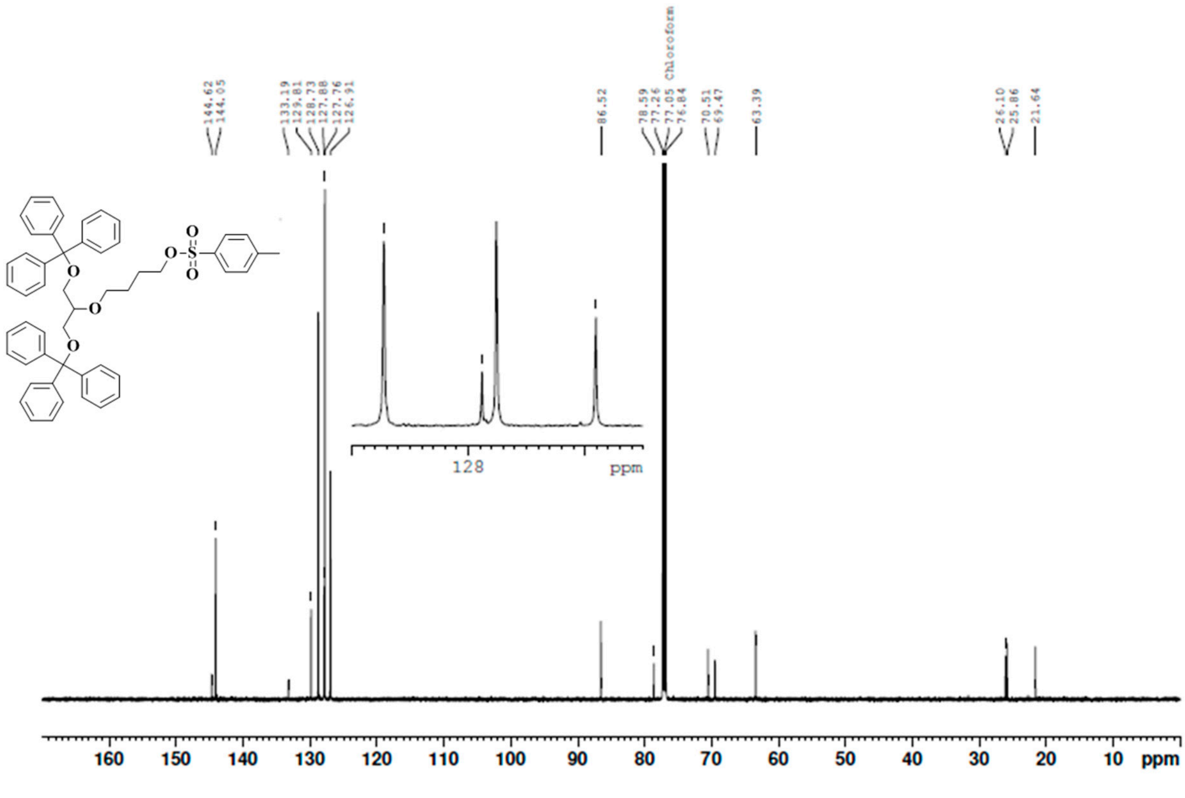

Figure S65. ^{13}C{^{1}H} NMR spectrum of 4-(1,3-bis(trityloxy)propan-2-yloxy)butyl4-methylbenzenesulfonate **(20)**.

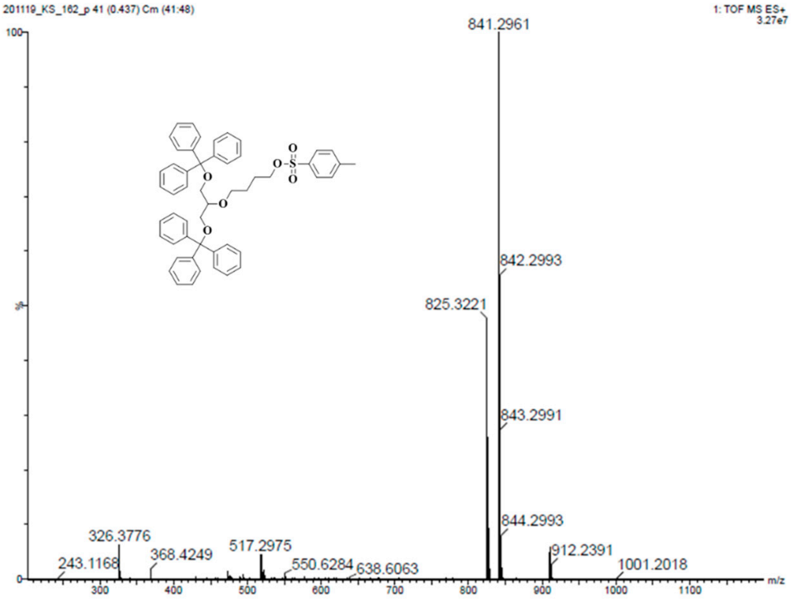

Figure S66. MS (ESI) spectrum of 4-(1,3-bis(trityloxy)propan-2-yloxy)butyl4-methylbenzenesulfonate **(20)**.

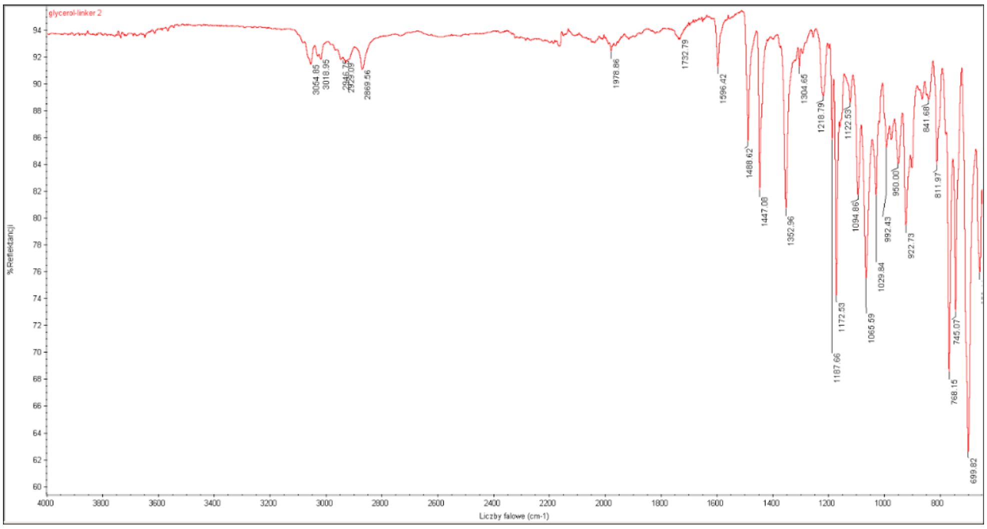

Figure S67. FT-IR spectrum of spectrum of 4-(1,3-bis(trityloxy)propan-2-yloxy)butyl4-methylbenzenesulfonate **(20)**.

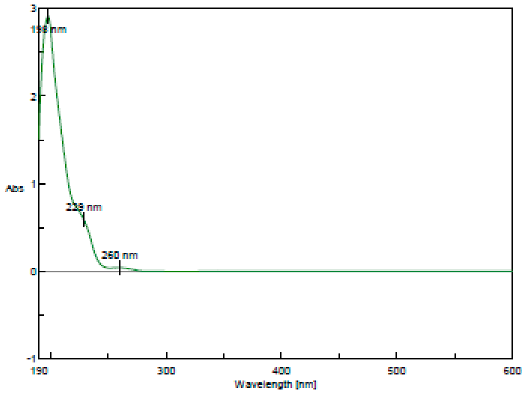

Figure S68. UV-VIS spectrum of 4-(1,3-bis(trityloxy)propan-2-yloxy)butyl4-methylbenzenesulfonate (20).

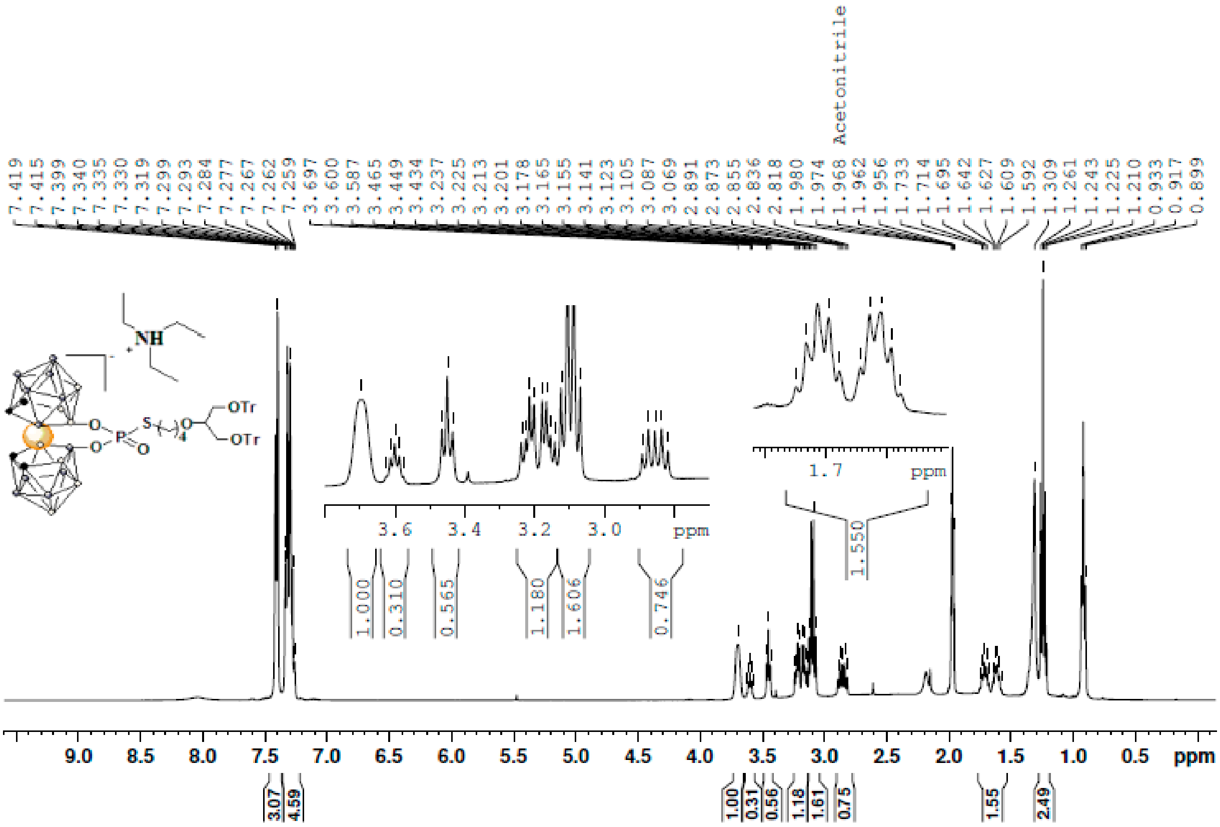

Figure S69. ^{1}H NMR spectrum of [8,8'-$O_2P(O)S(CH_2)_4OCH(CH_2OTr)_2$-3,3'-Co(1,2-$C_2B_9H_{10})_2$]$HNEt_3$ **(21)**.

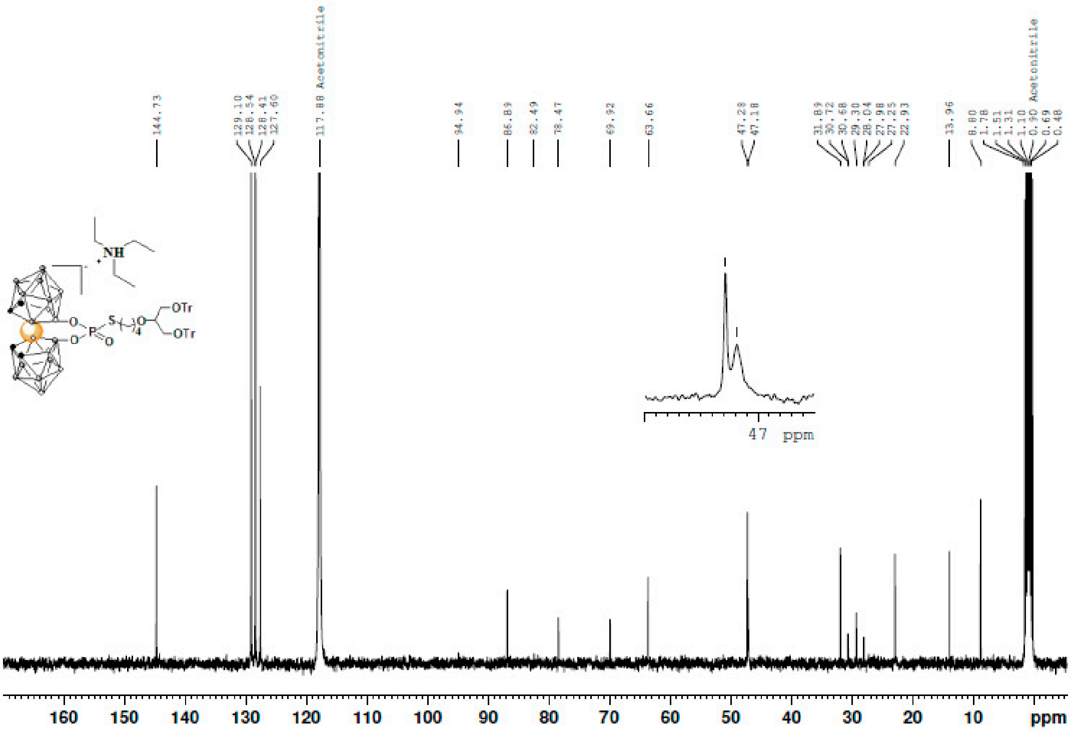

Figure S70. $^{13}C\{^1H\}$ NMR spectrum of [8,8'-$O_2P(O)S(CH_2)_4OCH(CH_2OTr)_2$-3,3'-Co(1,2-$C_2B_9H_{10})_2$]$HNEt_3$ **(21)**.

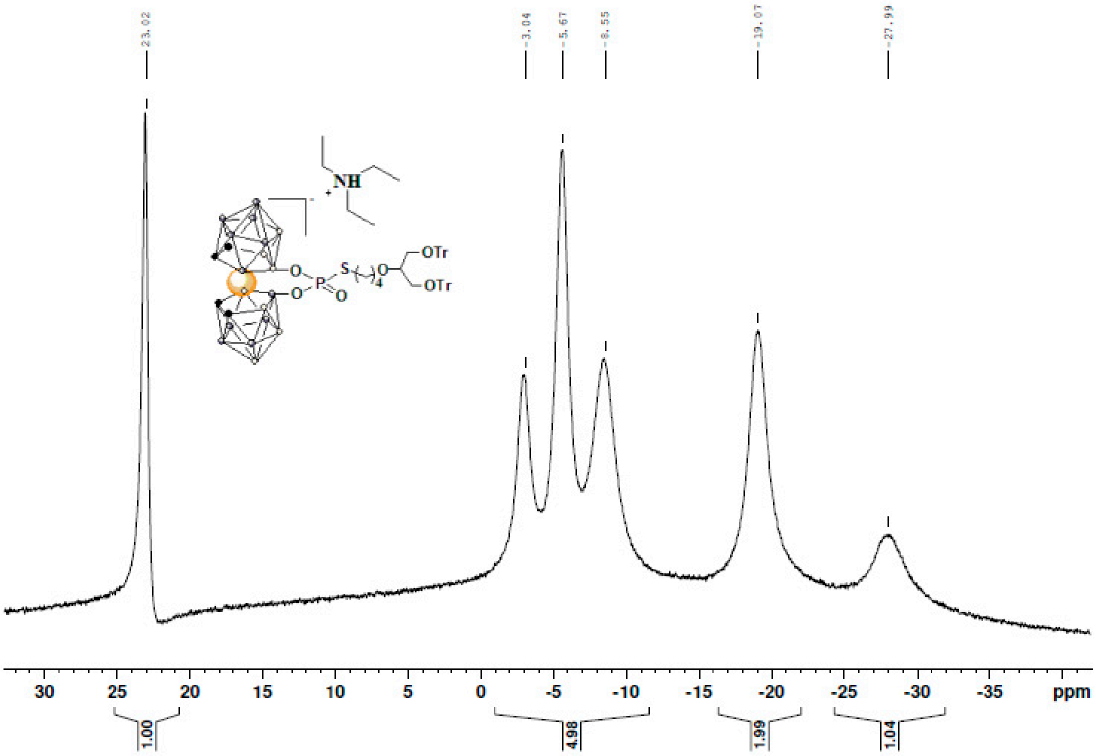

Figure S71. $^{11}B\{^{1}H\}$ NMR spectrum of [8,8'-$O_2P(O)S(CH_2)_4OCH(CH_2OTr)_2$-3,3'-Co(1,2-$C_2B_9H_{10})_2$]$HNEt_3$ **(21)**.

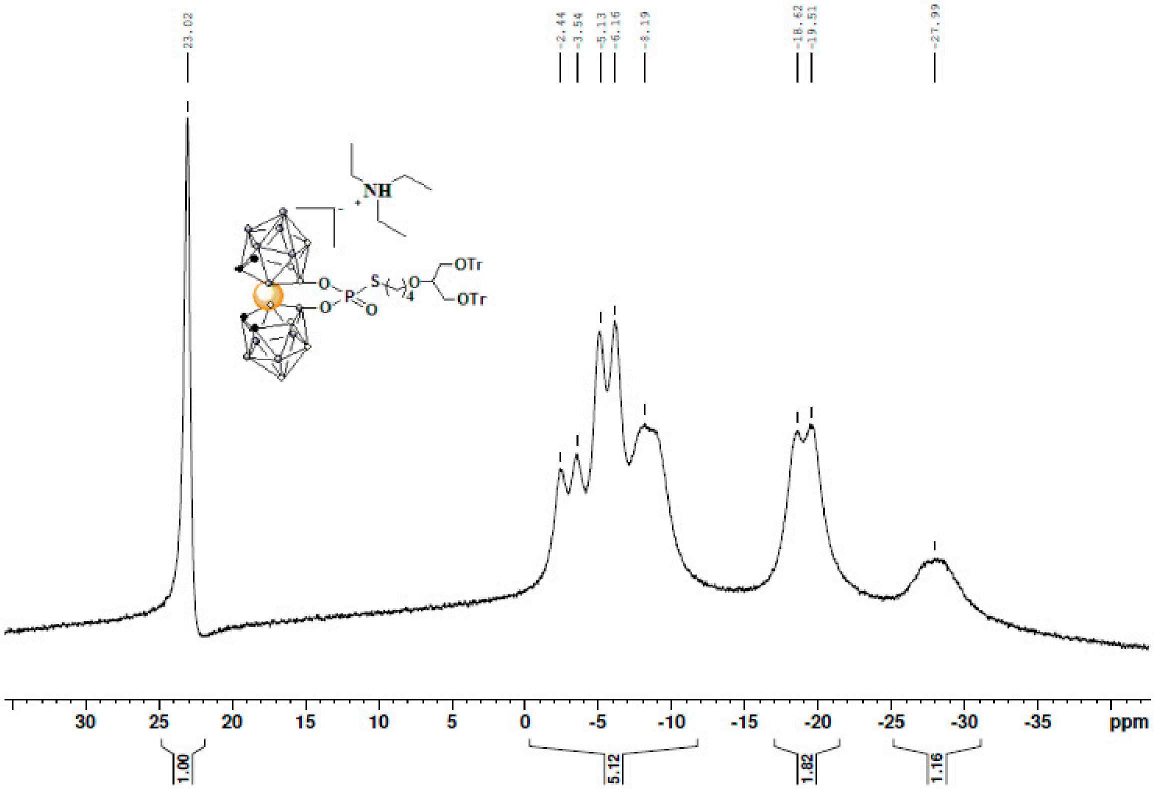

Figure S72. ^{11}B NMR spectrum of [8,8'-$O_2P(O)S(CH_2)_4OCH(CH_2OTr)_2$-3,3'-Co(1,2-$C_2B_9H_{10})_2$]$HNEt_3$ **(21)**.

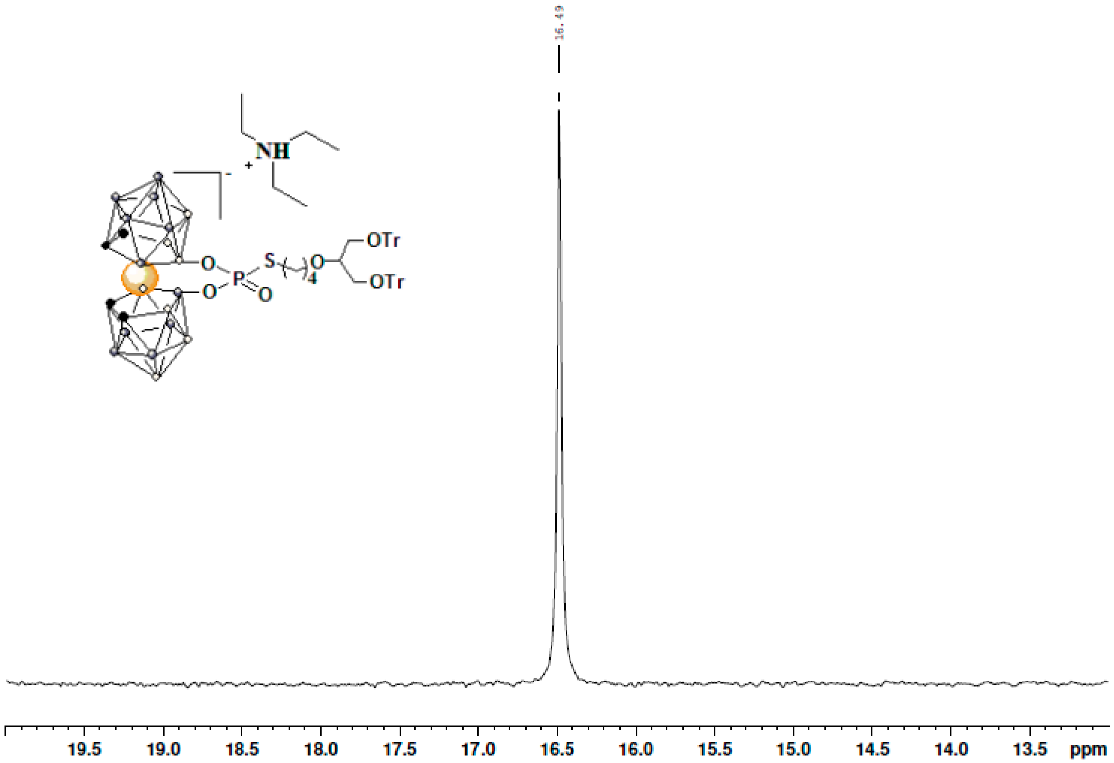

Figure S73. $^{31}P\{^1H\}$ NMR spectrum of [8,8'-$O_2P(O)S(CH_2)_4OCH(CH_2OTr)_2$-3,3'-Co(1,2-$C_2B_9H_{10})_2$]$HNEt_3$ **(21)**.

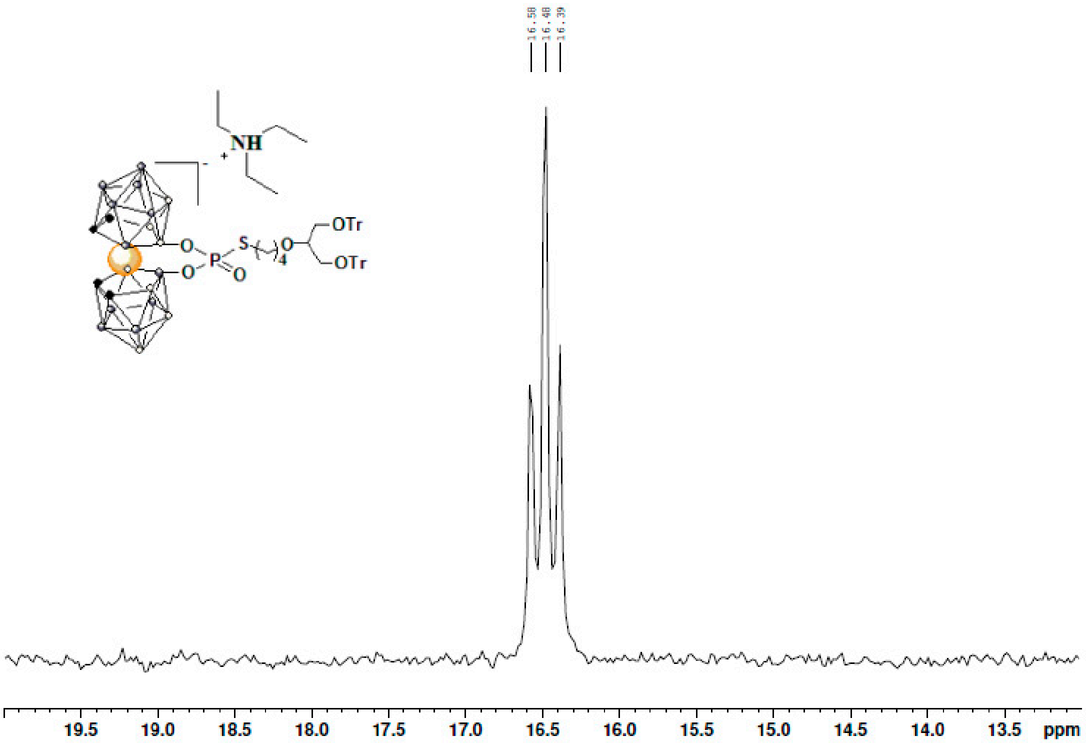

Figure S74. ^{31}P NMR spectrum of [8,8'-$O_2P(O)S(CH_2)_4OCH(CH_2OTr)_2$-3,3'-Co(1,2-$C_2B_9H_{10})_2$]$HNEt_3$ **(21)**.

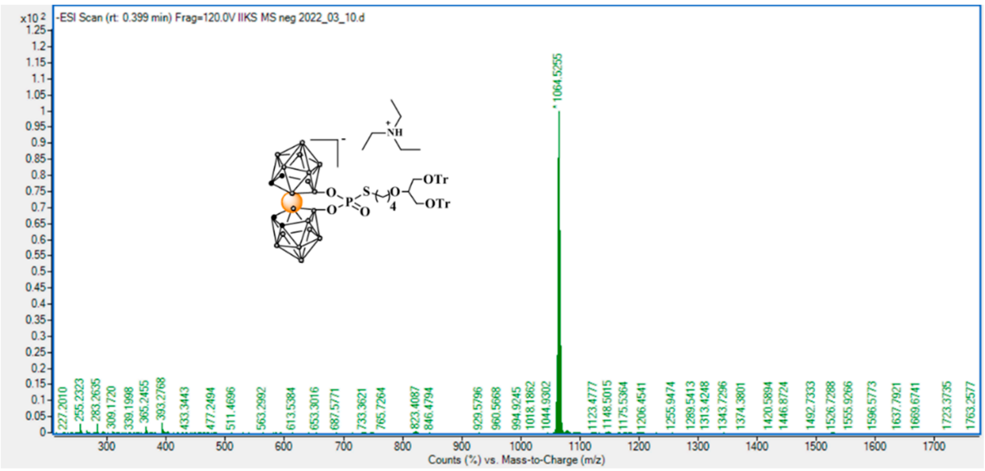

Figure S75. MS (ESI) spectrum of [8,8'-$O_2P(O)S(CH_2)_4OCH(CH_2OTr)_2$-3,3'-Co(1,2-$C_2B_9H_{10})_2$]$HNEt_3$ **(21)**.

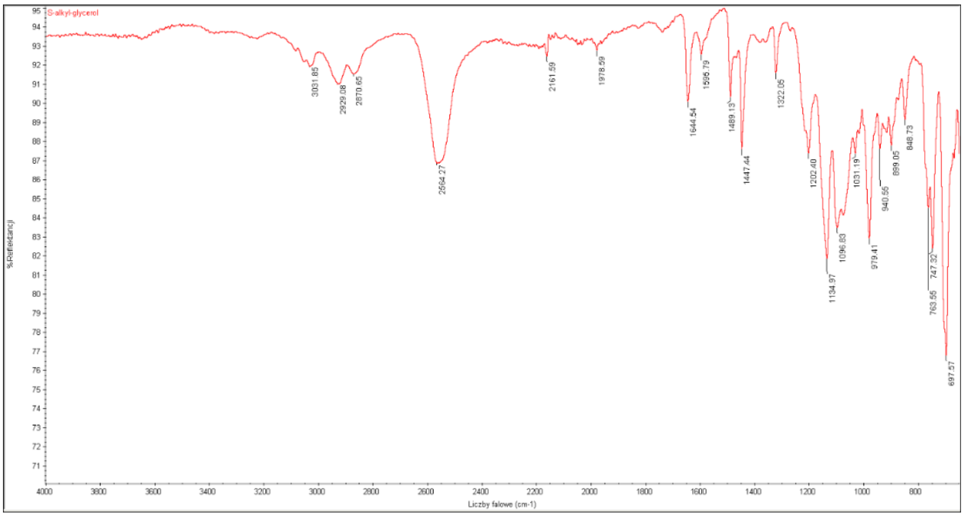

Figure S76. MS (ESI) spectrum of [8,8'-$O_2P(O)S(CH_2)_4OCH(CH_2OTr)_2$-3,3'-Co(1,2-$C_2B_9H_{10})_2$]$HNEt_3$ **(21)**.

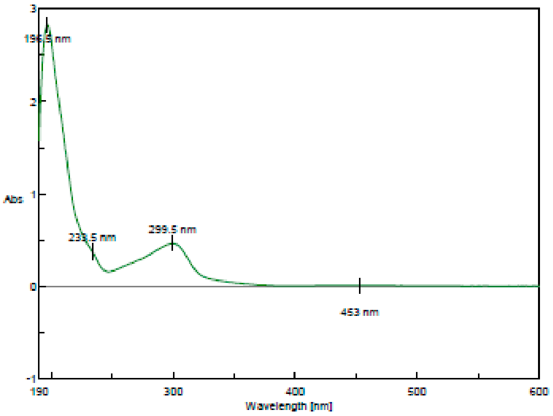

Figure S77. UV-VIS spectrum of spectrum of [8,8'-$O_2P(O)S(CH_2)_4OCH(CH_2OTr)_2$-3,3'-Co(1,2-$C_2B_9H_{10})_2$]$HNEt_3$ **(21)**.

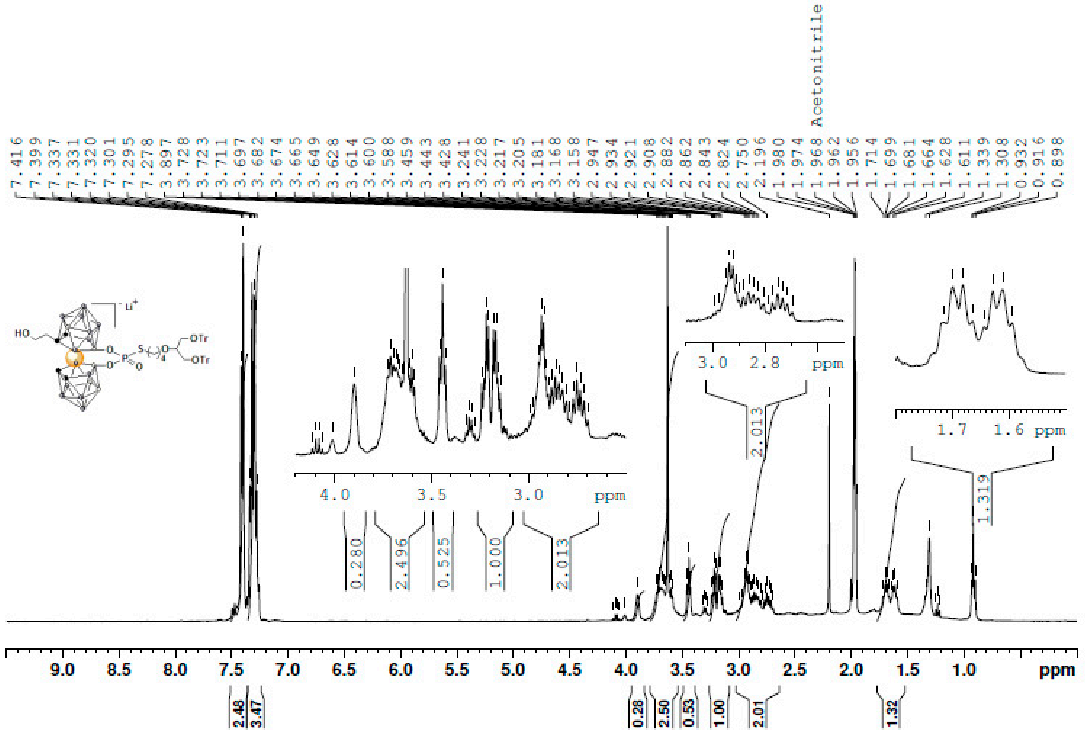

Figure S78. ^{1}H NMR spectrum of {8,8'-$O_2P(O)S[(CH_2)_4OCH(CH_2OCPh_3)_2]$-3,3'-Co[1-$(CH_2)_2OH$-1,2-$C_2B_9H_{10}$)](1',2'-$C_2B_9H_{10}$)} **(22)**.

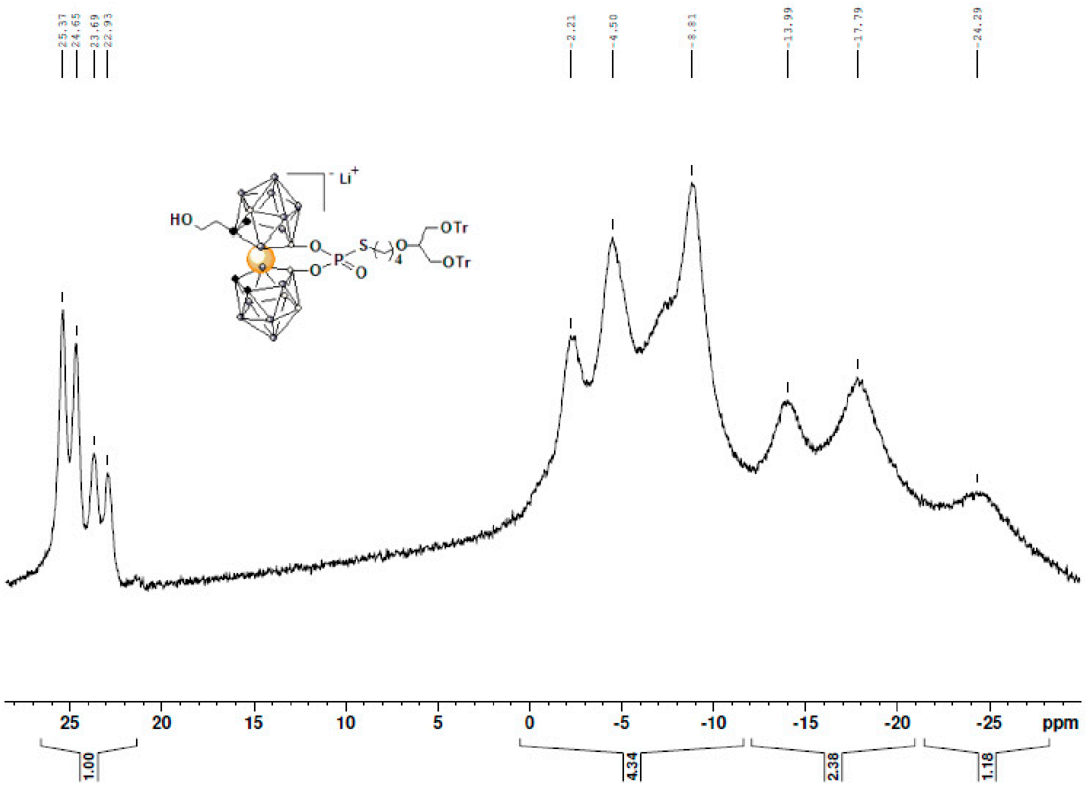

Figure S79. ^{11}B{^{1}H} NMR spectrum of {8,8'-$O_2P(O)S[(CH_2)_4OCH(CH_2OCPh_3)_2]$-3,3'-Co[1-$(CH_2)_2OH$-1,2-$C_2B_9H_{10}$)](1',2'-$C_2B_9H_{10}$)} **(22)**.

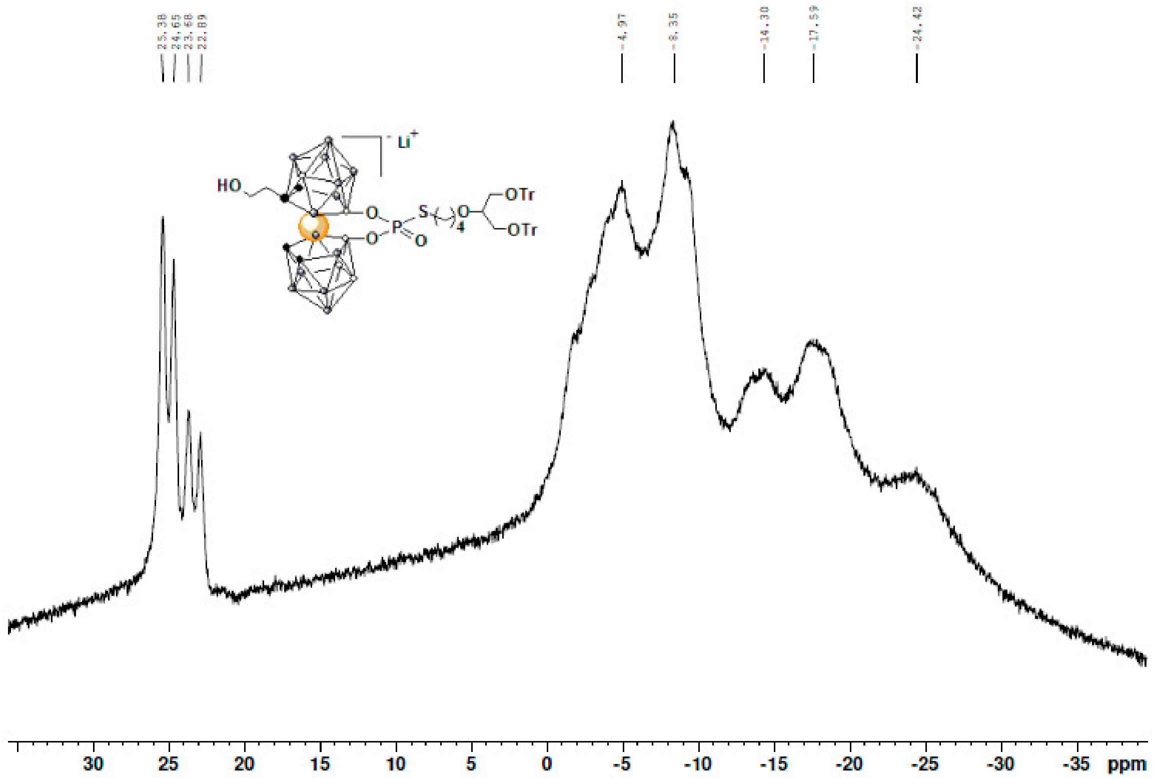

Figure S80. ^{11}B NMR spectrum of {8,8'-$O_2P(O)S[(CH_2)_4OCH(CH_2OCPh_3)_2]$-3,3'-Co[1-$(CH_2)_2OH$-1,2-$C_2B_9H_{10}$)](1',2'-$C_2B_9H_{10}$)} **(22)**.

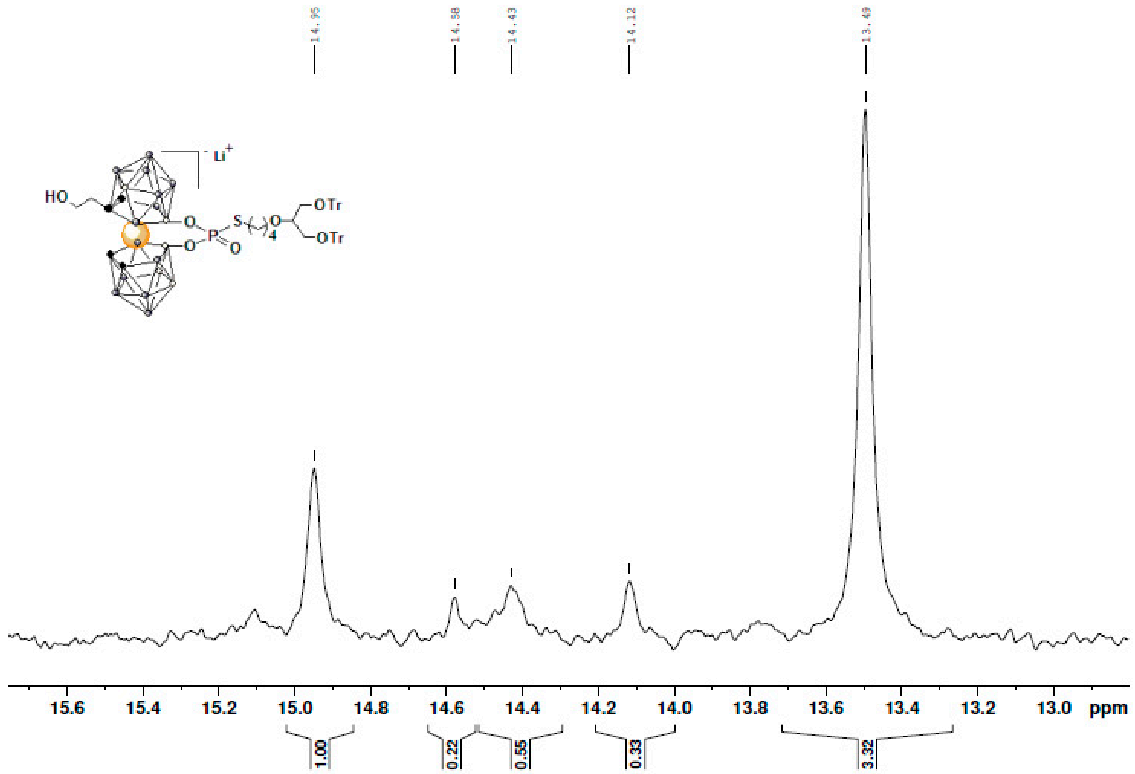

Figure S81. $^{31}P\{^1H\}$ NMR spectrum of {8,8'-$O_2P(O)S[(CH_2)_4OCH(CH_2OCPh_3)_2]$-3,3'-Co[1-$(CH_2)_2OH$-1,2-$C_2B_9H_{10}$)](1',2'-$C_2B_9H_{10}$)} **(22)**.

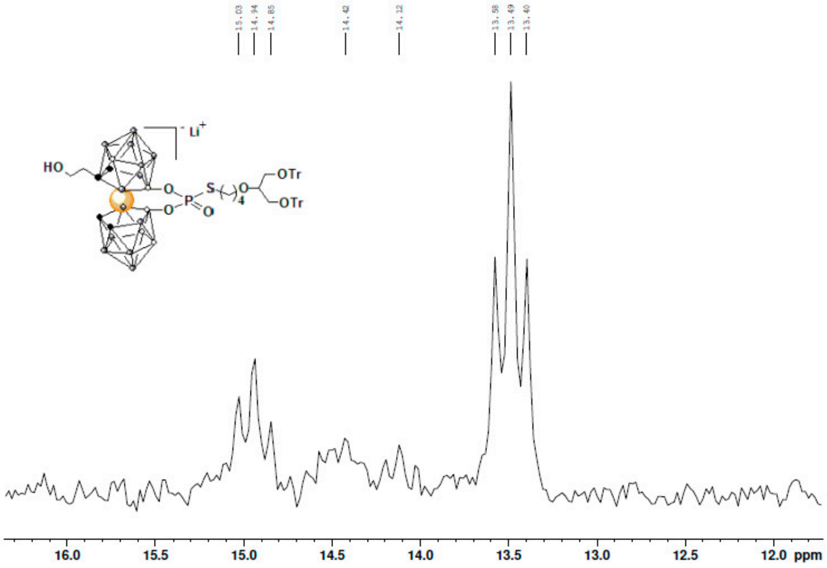

Figure S82. ^{31}P NMR spectrum of {8,8'-$O_2P(O)S[(CH_2)_4OCH(CH_2OCPh_3)_2]$-3,3'-Co[1-$(CH_2)_2OH$-1,2-$C_2B_9H_{10}$)](1',2'-$C_2B_9H_{10}$)} **(22)**.

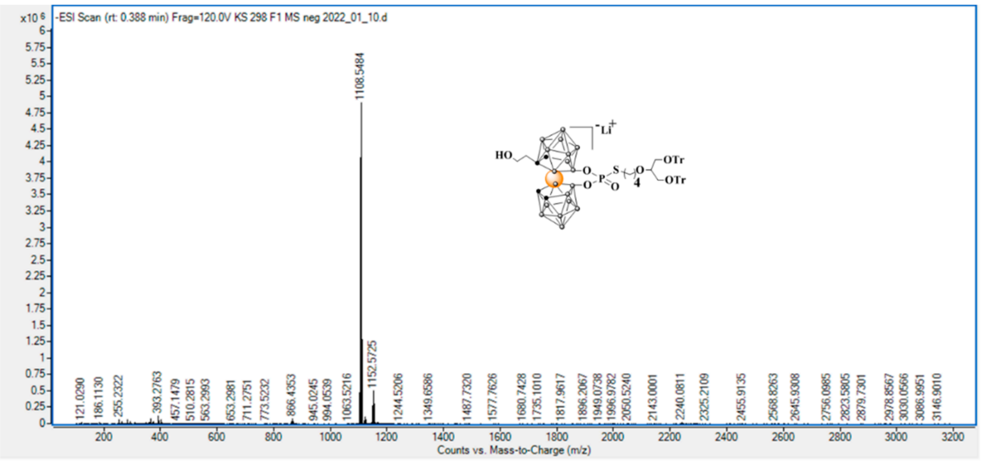

Figure S83. MS (ESI) spectrum of {8,8'-$O_2P(O)S[(CH_2)_4OCH(CH_2OCPh_3)_2]$-3,3'-Co[1-$(CH_2)_2OH$-1,2-$C_2B_9H_{10}$)](1',2'-$C_2B_9H_{10}$)} **(22)**.

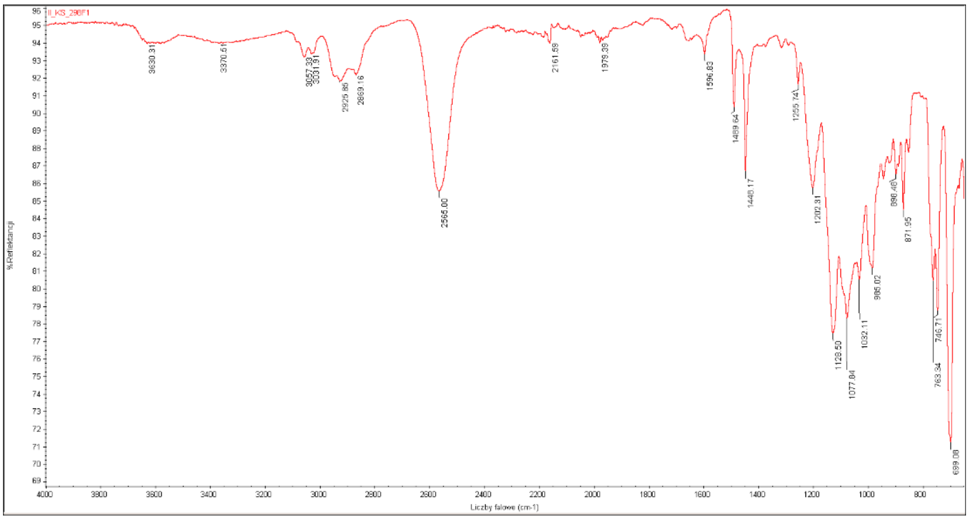

Figure S84. FT-IR spectrum of {8,8'-$O_2P(O)S[(CH_2)_4OCH(CH_2OCPh_3)_2]$-3,3'-Co[1-$(CH_2)_2OH$-1,2-$C_2B_9H_{10}$)](1',2'-$C_2B_9H_{10}$)} **(22)**.

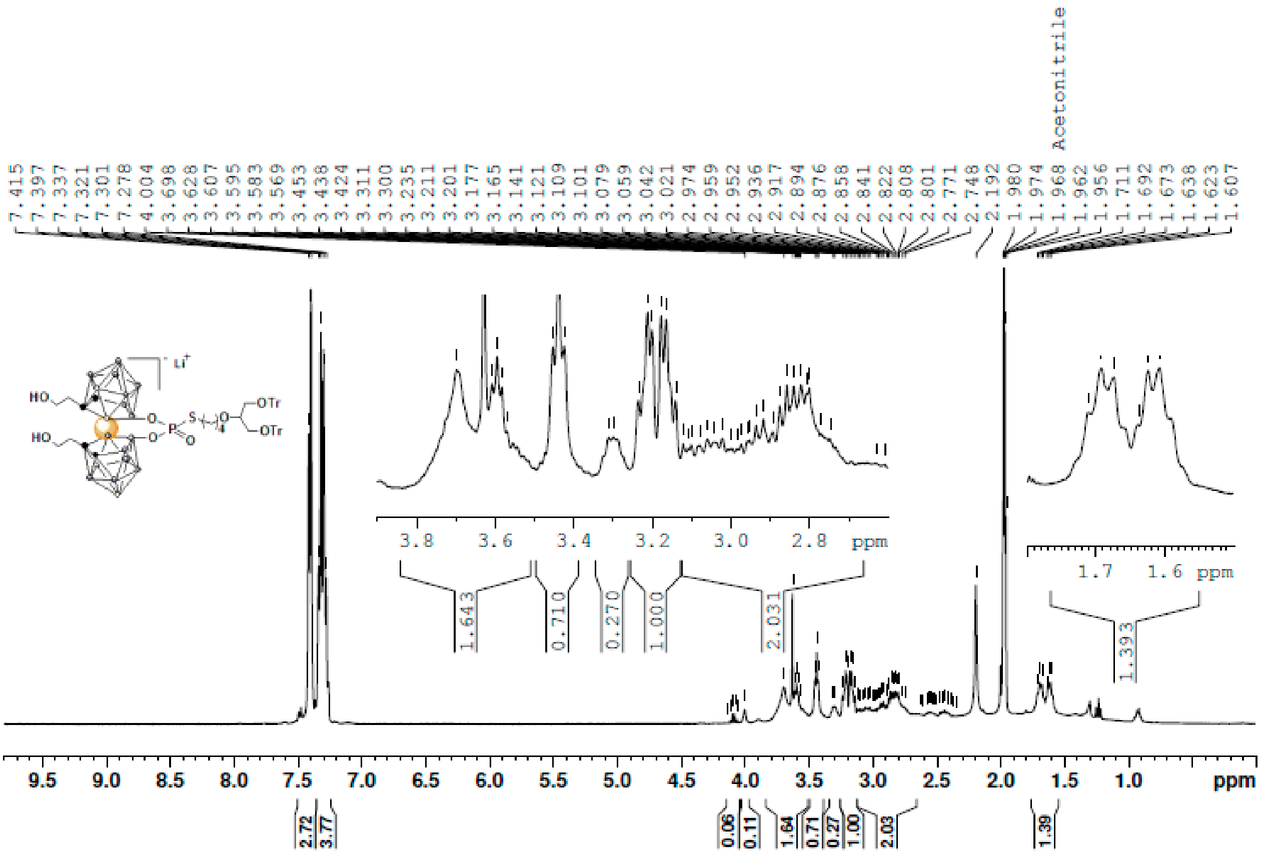

Figure S85. ^{1}H NMR spectrum of {8,8'-$O_2P(O)S[(CH_2)_4OCH(CH_2OCPh_3)_2]$-3,3'-Co[1-$(CH_2)_2OH$-1,2-$C_2B_9H_{10}$)] [1’-$(CH_2)_2OH$-1’,2’-$C_2B_9H_{10}$)]} $HNEt_3$ **(23)**.

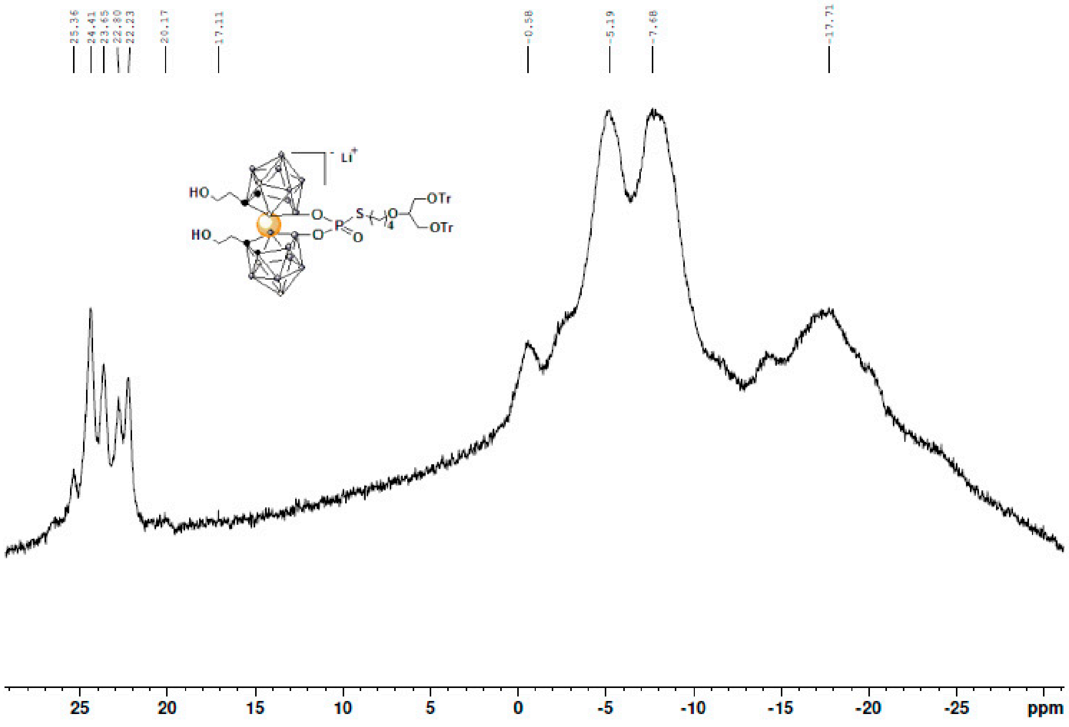

Figure S86. $^{11}B\{^{1}H\}$ NMR spectrum of {8,8'-$O_2P(O)S[(CH_2)_4OCH(CH_2OCPh_3)_2]$-3,3'-Co[1-$(CH_2)_2OH$-1,2-$C_2B_9H_{10}$)] [1’-$(CH_2)_2OH$-1’,2’-$C_2B_9H_{10}$)]} $HNEt_3$ **(23)**.

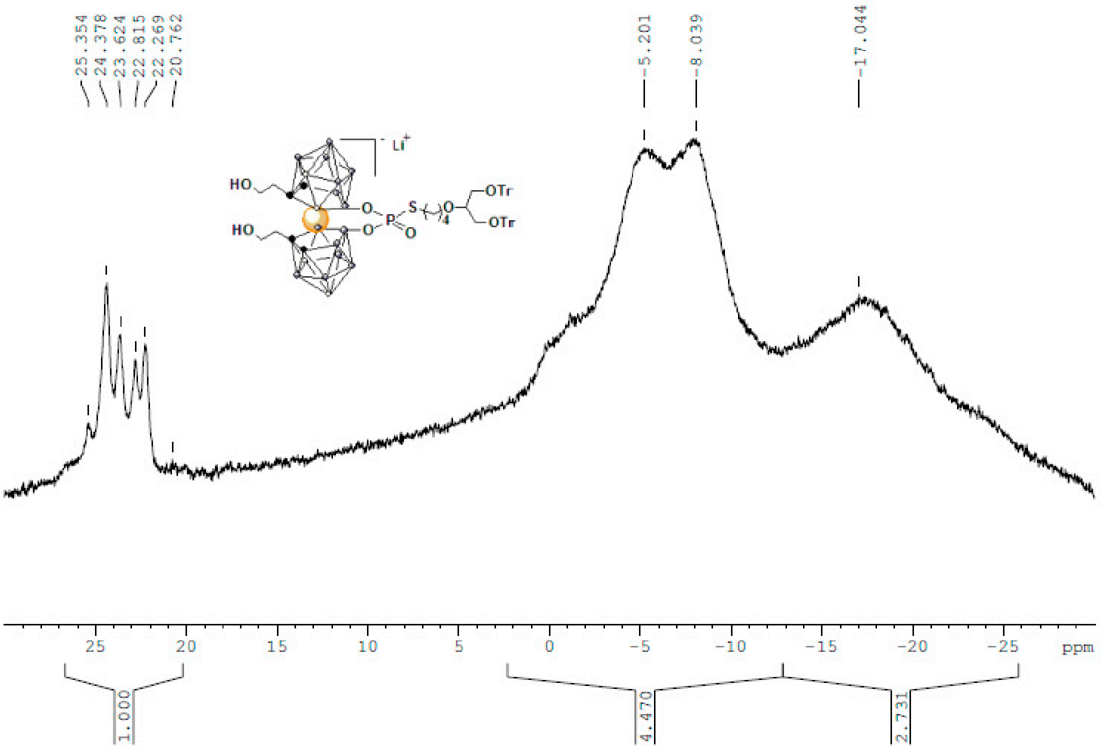

Figure S87. ^{11}B NMR spectrum of {8,8'-$O_2P(O)S[(CH_2)_4OCH(CH_2OCPh_3)_2]$-3,3'-Co[1-$(CH_2)_2OH$-1,2-$C_2B_9H_{10}$)] [1'-$(CH_2)_2OH$-1',2'-$C_2B_9H_{10}$)]} $HNEt_3$ **(23)**.

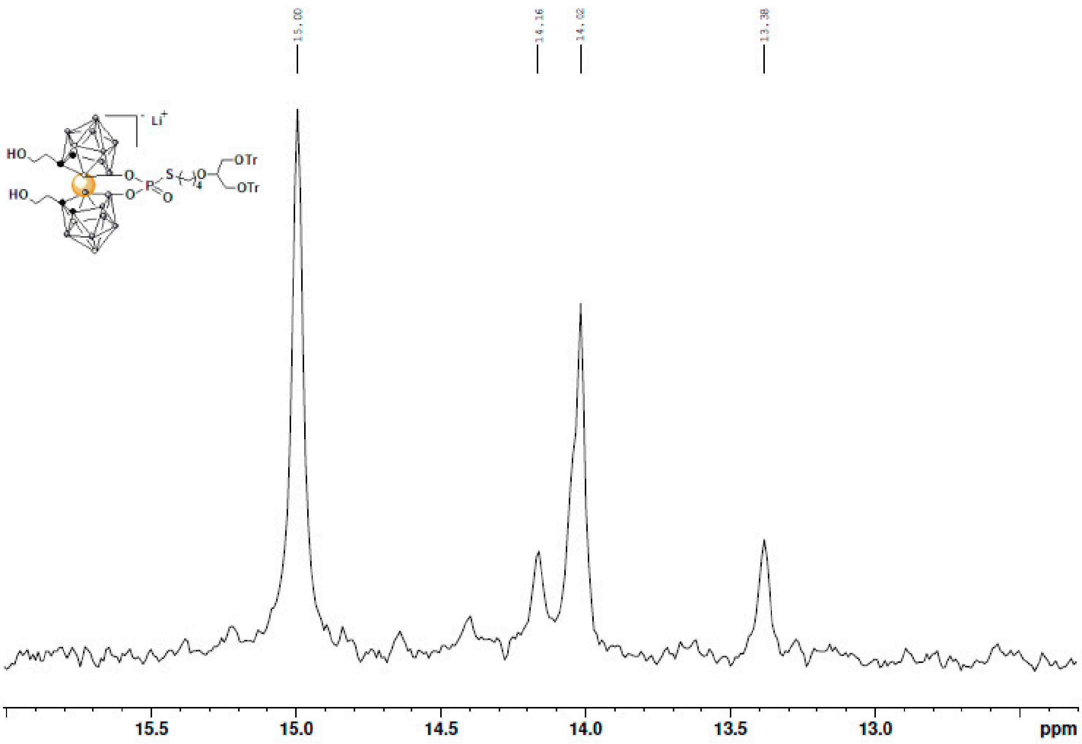

Figure S88. $^{31}P\{^1H\}$ NMR spectrum of {8,8'-$O_2P(O)S[(CH_2)_4OCH(CH_2OCPh_3)_2]$-3,3'-Co[1-$(CH_2)_2OH$-1,2-$C_2B_9H_{10}$)] [1'-$(CH_2)_2OH$-1',2'-$C_2B_9H_{10}$)]} $HNEt_3$ **(23)**.

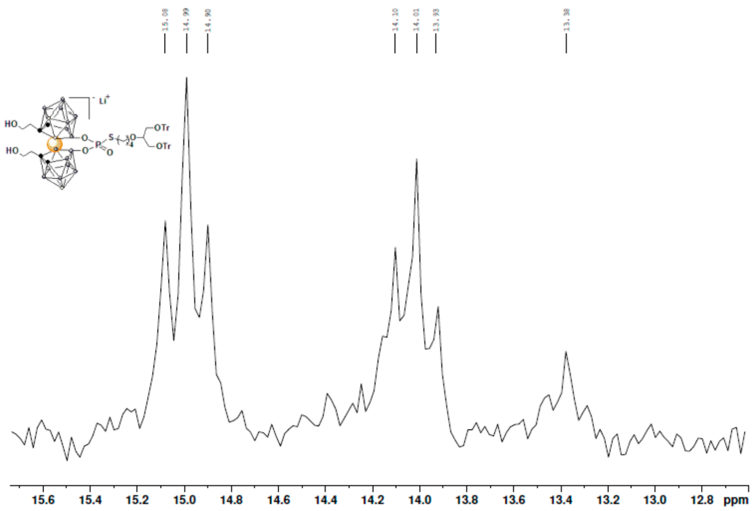

Figure S89. ^{31}P NMR spectrum of {8,8'-$O_2P(O)S[(CH_2)_4OCH(CH_2OCPh_3)_2]$-3,3'-Co[1-$(CH_2)_2OH$-1,2-$C_2B_9H_{10}$)] [1'-$(CH_2)_2OH$-1',2'-$C_2B_9H_{10}$)]} **$HNEt_3$ (23)**

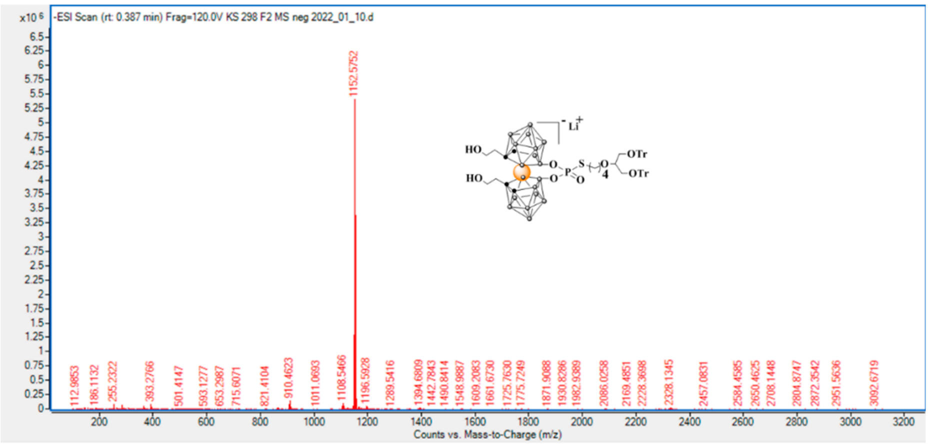

Figure S90. ESI (MS) spectrum of {8,8'-$O_2P(O)S[(CH_2)_4OCH(CH_2OCPh_3)_2]$-3,3'-Co[1-$(CH_2)_2OH$-1,2-$C_2B_9H_{10}$)] [1'-$(CH_2)_2OH$-1',2'-$C_2B_9H_{10}$)]} $HNEt_3$ **(23)**.

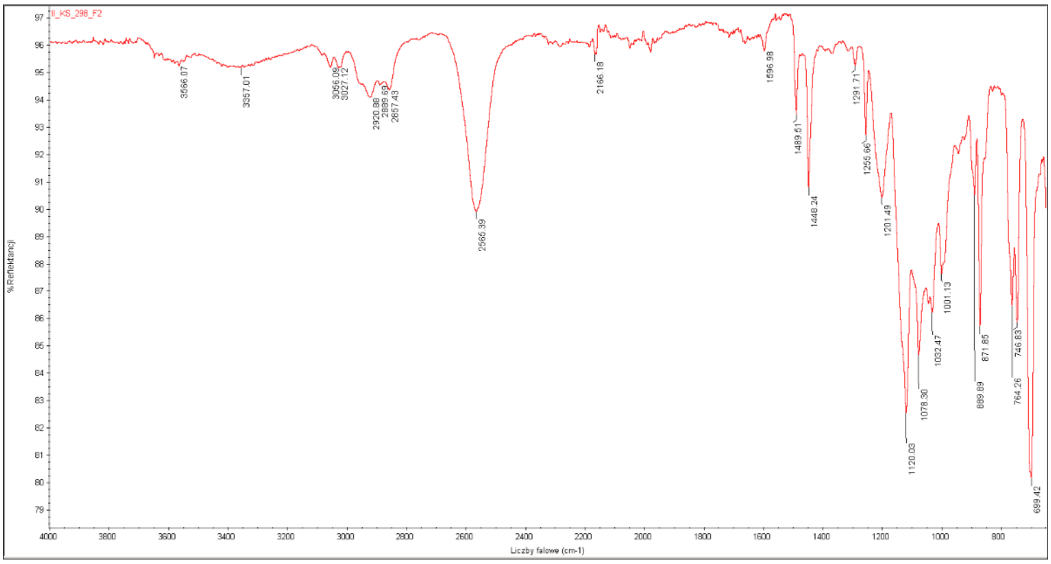

Figure S91. FT-IR spectrum of {8,8'-$O_2P(O)S[(CH_2)_4OCH(CH_2OCPh_3)_2]$-3,3'-Co[1-$(CH_2)_2OH$-1,2-$C_2B_9H_{10}$)] [1'-$(CH_2)_2OH$-1',2'-$C_2B_9H_{10}$)]} $HNEt_3$ **(23)**.
